# Pollutant Burden in Autism Spectrum Disorder: Mechanistic Convergence, Genetic Susceptibility, and Clinical Translation

**DOI:** 10.3390/cimb48090878

**Published:** 2026-08-29

**Authors:** George Ayoub

**Affiliations:** Psychology Department, Santa Barbara City College, Santa Barbara, CA 93109, USA; neuro@sbcc.edu

**Keywords:** autism spectrum disorder, environmental pollutants, micro/nanoplastics, endocrine-disrupting chemicals, oxidative stress, neuroinflammation, folate receptor autoantibody, critical periods of neurodevelopment, gut microbiome, cerebral folate deficiency

## Abstract

**Highlights:**

**What are the main findings?**

- Six major classes of environmental pollutants—micro/nanoplastics, plastic-associated endocrine-disrupting chemicals, ambient/indoor air pollution, tire wear particles, heavy metals and pesticides, and industrial chemicals/POPs—converge on a shared set of mechanisms (oxidative stress, neuroinflammation, endocrine disruption, and impaired microglial synaptic pruning) that plausibly contribute to ASD risk, largely through the oxidative stress/inflammation pathway of our two-path model. Ultra-processed food intake is reviewed alongside them as a parallel, non-pollutant contributor to the same inflammatory pathway, not as a seventh pollutant class.
- Human evidence for micro/nanoplastics has moved beyond simple detection to include placental genotoxicity, direct oxidative stress effects in human placental tissue, and fetal endocrine correlates, complementing an extensive rodent literature that directly links prenatal/early-life exposure to ASD-relevant social and repetitive behavior deficits. Exposure timing relative to specific developmental windows, rather than cumulative dose alone, appears to determine which process is disrupted.

**What are the implications of the main findings?**

- Genetic variation in folate pathway and mitochondrial genes, as well as individual differences in folate/ vitamin B sufficiency, are proposed as a candidate effect modifier rather than an established protective factor to modify susceptibility to pollutant-driven oxidative stress across the six pollutant classes. This remains a testable gene-by-pollutant research priority, not yet a validated finding.
- Because several of these exposures are ubiquitous and largely unregulated, meaningful risk reduction likely requires population-level policy action (e.g., additive restrictions, expanded biomonitoring, air quality standards) alongside individual-level, low-risk strategies such as dietary pattern and household exposure reduction, rather than relying on individual behavior change alone.

**Abstract:**

Autism spectrum disorder (ASD) risk reflects genetic susceptibility and modifiable environmental exposures acting during fetal and early postnatal critical periods. Building on our prior two-path model, in which folate receptor autoantibody-driven cerebral folate deficiency and oxidative stress/neuroinflammation converge on disrupted neurodevelopment, this review provides the first full mechanistic treatment of environmental pollutants within that framework. We synthesize evidence across seven exposure categories: micro/nanoplastics, plastic-associated endocrine-disrupting chemicals, ambient/indoor air pollution, tire wear particles and 6PPD-quinone, heavy metals and pesticides, industrial chemicals and persistent organic pollutants, and ultra-processed food intake as a parallel, non-pollutant contributor to the same inflammatory pathway. Human biomonitoring of micro/nanoplastics has progressed beyond detection in the placenta, brain and breast milk to direct evidence of placental genotoxicity and fetal endocrine disruption, complementing rodent data linking early-life exposure to impaired corticogenesis, disrupted microglial synaptic pruning, and ASD-relevant behavioral deficits. Across categories, oxidative stress, barrier disruption, neuroinflammation, endocrine disruption, and epigenetic modification recur as convergent mechanisms acting on trimester- and age-specific windows of vulnerability. Genetic variation in folate pathway and mitochondrial genes, as well as folate/vitamin B sufficiency, are proposed as candidate effect modifiers rather than established protective factors to modify susceptibility to this pollutant burden. Most evidence is associational or mechanistic rather than trial-based; we grade evidence strength and translate findings into biomarker-guided clinical and population-level policy guidance.

## 1. Introduction

Autism spectrum disorder (ASD) is a neurodevelopmental condition whose rising prevalence cannot be fully explained by changes in diagnostic criteria or ascertainment alone. As with the critical-period framework we previously proposed for folate availability and oxidative stress reduction in ASD risk [1], we suggest that the fetal period and the first several postnatal years constitute a window during which environmental exposures, acting on neurogenesis, synaptogenesis, myelination, and activity-dependent synaptic pruning, can shift developmental trajectories toward or away from ASD-associated phenotypes [2]. This view is consistent with broader syntheses estimating that environmental factors may account for as much as half of ASD risk variance across parental, prenatal, perinatal, and chemical exposure domains [3], and with our own prior work situating genetic susceptibility, maternal immune activation, autoimmune disease, and folate-related metabolic vulnerability within a shared inflammatory and oxidative stress framework [2,4].

The developmental neurotoxicant paradigm itself is not new: Grandjean and Landrigan’s landmark reviews identified lead, methylmercury, polychlorinated biphenyls, arsenic, and toluene as proven human developmental neurotoxicants in 2006 [5], later expanding the list to include manganese, fluoride, chlorpyrifos, DDT, tetrachloroethylene, and polybrominated diphenyl ethers in 2014, and explicitly framing the rise of ASD, ADHD, and dyslexia as a possible “pandemic of developmental neurotoxicity” [6]. What has changed in the past decade is the recognition that microplastics and nanoplastics, along with the endocrine-disrupting chemicals leached from and used to manufacture plastics, constitute a distinct, ubiquitous, and largely unregulated exposure class that plausibly belongs on this list.

**Figure 1:**
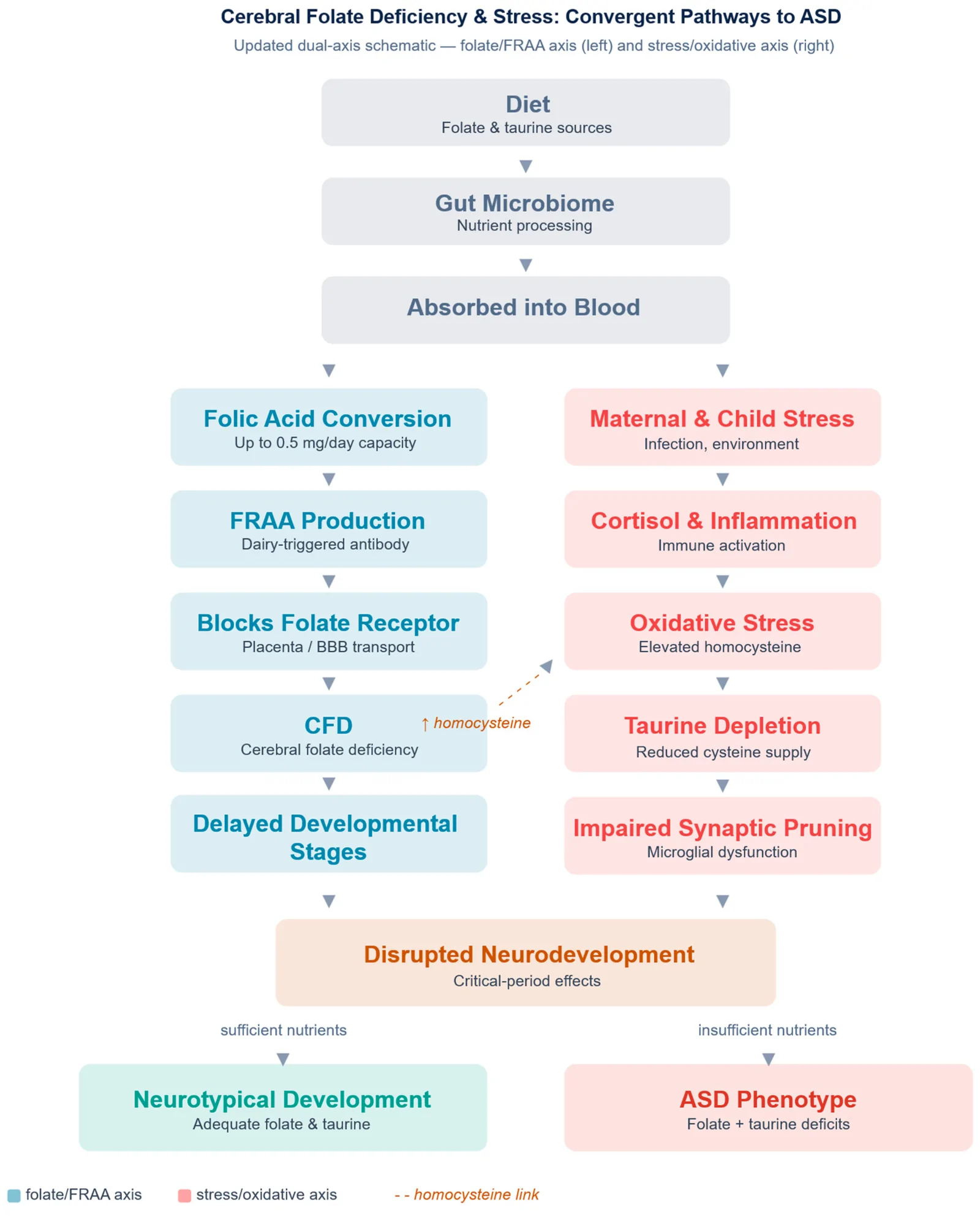
The two-path model of ASD-related neurodevelopmental risk, updated from our prior work [1]. Diet-derived folate and taurine are processed by the gut microbiome and absorbed into the maternal/fetal circulation, then diverge along two mechanistically distinct but convergent routes: a folate/FRAA axis (left), in which folate receptor autoantibodies block placental and blood–brain barrier folate transport, producing cerebral folate deficiency; and a stress/oxidative axis (right), in which maternal or child stress and inflammation elevate cortisol and oxidative stress, deplete taurine, and impair microglial synaptic pruning. The two axes cross-communicate via homocysteine and converge on disrupted critical-period neurodevelopment.

We previously proposed a two-path model of ASD-related neurodevelopmental risk [1]. Path A centers on folate: up to 70% of ASD children carry a folate receptor autoantibody (FRAA) that blocks the placental and blood–brain barrier transport mediated by folate receptor alpha, producing cerebral folate deficiency (CFD); dairy elimination lowers FRAA titers, and supplementation with reduced folate (methylfolate or folinic acid) improves communication outcomes in roughly two-thirds of FRAA-positive children treated at younger ages (most positive outcomes under 6 years, with good effect under 11–12 years), with efficacy dropping sharply thereafter. Path B centers on oxidative stress and inflammation: taurine, synthesized from cysteine, acts as an antioxidant and anti-inflammatory agent that supports microglial-mediated synaptic pruning; when taurine or cysteine availability falls, whether from maternal stress, infection, or inadequate dietary intake, microglial suppression and impaired pruning follow, and N-acetylcysteine supplementation has been shown to reduce hyperactivity in a controlled trial. Figure 1 reproduces this model in updated form.

Our subsequent work broadened Path B considerably, situating maternal immune activation, autoimmune disease, and eleven categories of inflammatory trigger—spanning viral infection, air pollution, maternal asthma, microplastics, malnutrition, emotional stress, pharmaceuticals, maternal metabolic disease, synthetic vitamins, vaccines, and microbiome/metabolic disorders—within a shared gene-by-environment framework converging on oxidative stress and neuroinflammation [2]. That paper’s treatment of microplastics was necessarily brief, with a single subsection surveying a handful of animal studies. This present review takes that subsection and expands it into a full mechanistic treatment, organized by pollutant class rather than folded into a broader inflammation category, while also substantially deepening the mitigation and translation guidance that our prior work could only sketch. This is in no small part thanks to the abundance of recent evidence from large-scale studies evaluating pollutants’ impact on development, which are cited throughout this review, and of which the key findings are detailed by reference in the Supplemental Material accompanying this review.

**Figure 2:**
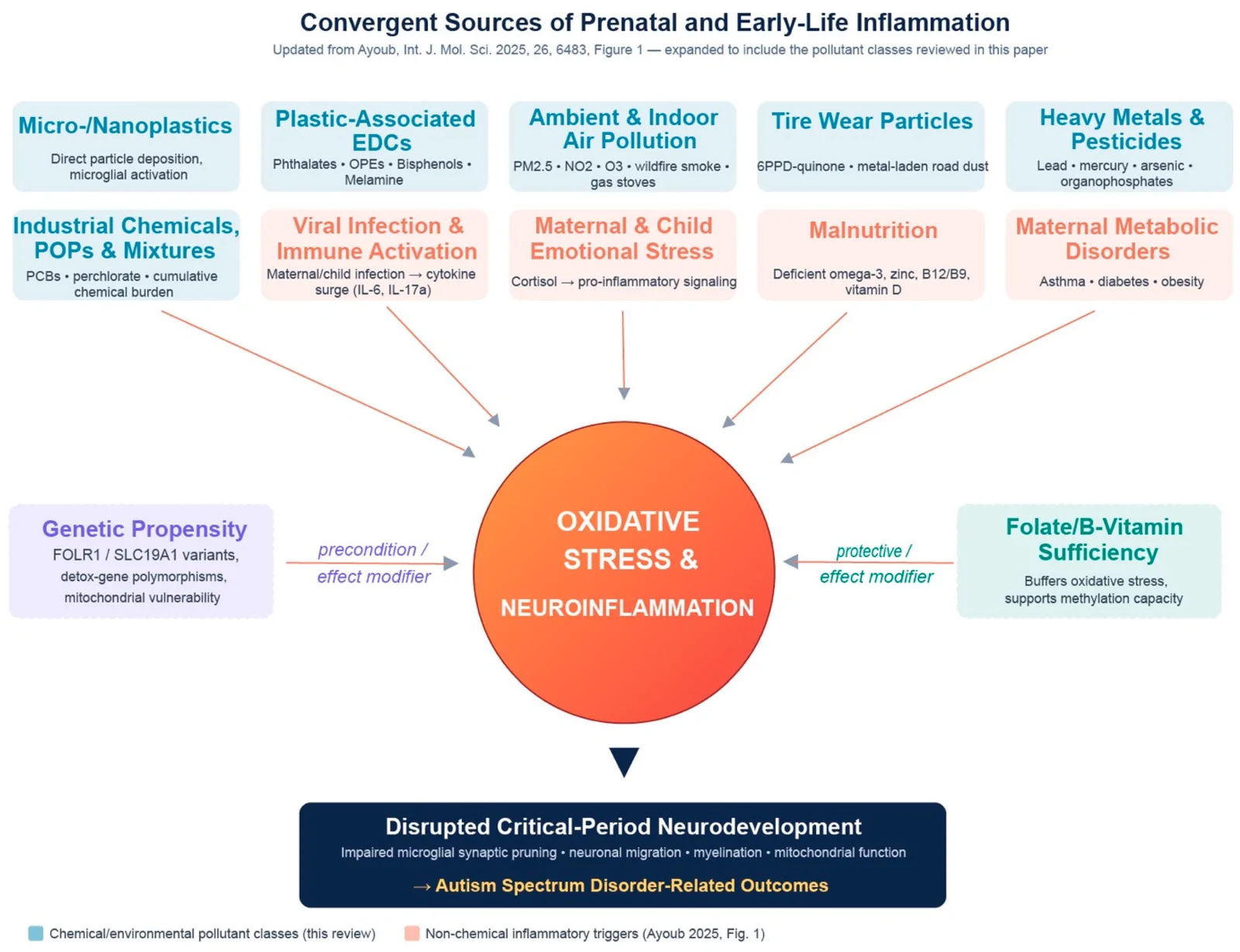
Convergent sources of prenatal and early-life inflammation feeding the stress/oxidative axis in Figure 1, updated from our prior work [2]. The six environmental pollutant classes reviewed in this paper (blue) join the non-chemical inflammatory triggers identified in [2] (coral) in converging on oxidative stress and neuroinflammation, modified by genetic propensity and by folate/vitamin B sufficiency as a proposed, not yet established, buffering factor en route to disrupted critical-period neurodevelopment.

Environmental pollutants matter to this model for two reasons. First, and most directly, several of the pollutant classes reviewed here—ambient and indoor air pollution, tire wear particles, heavy metals and pesticides, and industrial chemicals—act principally through Path B: they provoke maternal or fetal oxidative stress and systemic inflammation through the same cytokine and reactive oxygen species pathways implicated in our prior work (ultra-processed food intake, reviewed in Section 2.7, shows a parallel signal through the same pathway but is a dietary pattern rather than a pollutant, and we discuss it separately for that reason) [1,2]. Second, and more speculatively, pollutant-driven oxidative stress may secondarily compromise Path A: elevated homocysteine from oxidative disruption of one-carbon metabolism is mechanistically continuous with the folate-transport pathway, and whether pollutant burden itself increases FRAA production is, to our knowledge, untested but testable, a hypothesis we flag as a research priority rather than an established mechanism. Figure 2 updates the multi-source inflammation schematic from our prior work to include the pollutant classes reviewed here alongside the non-chemical triggers identified previously.

This review’s scope is organized by pollutant class (Section 2.1, Section 2.2, Section 2.3, Section 2.4, Section 2.5, Section 2.6 and Section 2.7), followed by a synthesis of convergent mechanisms (Section 3), the critical-periods framework governing when these exposures matter most (Section 5), the gut microbiome as a nutrient-processing modifier (Section 6), and nutrient status as a candidate effect modifier across these classes—a research hypothesis rather than an established protective factor (Section 7). Path A is treated only briefly here since it is developed in full in our prior work [1]; this review’s own contribution is concentrated on Path B and on the convergence between the two. As with that prior work, we state plainly that most of the evidence assembled here is associational, epidemiological, or drawn from animal mechanistic studies rather than human randomized trials; we flag study design and evidence strength throughout rather than treating all citations as equivalent.

In this review, we organize the evidence by pollutant class, present the preclinical and epidemiological findings for each, and close each section with speculation on the specific developmental process most plausibly disrupted. We then synthesize these findings into a set of convergent mechanisms and propose, as in our prior work, that nutrient status may act as an effect modifier across the six pollutant categories, mitigating at least in part their neurodevelopmental impact.

### Literature Search Strategy

This is a narrative rather than a systematic review, and we did not follow a PRISMA-style pre-registered protocol; we describe our search approach here so readers can judge its scope and limits. Literature was identified primarily through PubMed/MEDLINE, supplemented by Google Scholar and by forward/backward citation chasing from key reviews and cohort-study reports in each pollutant category, using search terms combining “autism” or “autism spectrum disorder” or “neurodevelopment” with the pollutant, mechanism, or biomarker terms specific to each subsection (for example, “micro/nanoplastics,” “phthalate,” “6PPD-quinone,” “folate receptor autoantibod*,” “cerebral folate deficiency,” “ultra-processed food”). Searches were last conducted in June 2026 and were not restricted by publication date, though we prioritized the most recent large cohort studies (ECHO, CHARGE, MARBLES, and comparable multi-site consortia) and the most recent systematic reviews or meta-analyses in each area where available. We used Claude AI (as disclosed in our Acknowledgments) to help identify and cross-check candidate sources, particularly for articles not indexed in PubMed, in addition to manual database searching; all AI-assisted search results were independently verified against the primary source before citation. We did not apply formal risk-of-bias or quality appraisal instruments to included studies, and inclusion decisions reflect the authors’ judgment of relevance and quality rather than a pre-specified, independently duplicated screening process; we discuss the consequences of this narrative approach further in Section 9.5 (Limitations).

## 2. Environmental Contributors to Neurodevelopmental Risk

### 2.1. Micro/Nanoplastics: Direct Particle Neurotoxicity

Micro- and nanoplastics (MNPs) are now detectable in human blood, placenta, and breast milk, establishing a plausible route from maternal exposure to the fetal brain. Table S1 in the Supplemental Files provides a summary list of each report described in this section, its major findings, and a link to the publication.

The human biomonitoring literature supporting that initial claim has grown substantially since it was first established, and now extends well beyond simple detection. Using pyrolysis gas chromatography–mass spectrometry, one team detected microplastics in all 62 placentas tested in a cohort study, with tissue concentrations ranging from 6.5 to 790 micrograms per gram [7]. The same group subsequently reported microplastics in every one of 24 post-mortem human brain samples examined, at concentrations roughly seven to thirty times higher than in paired liver and kidney tissue from the same donors. Brain samples collected in 2024 contained substantially more plastic by mass than samples collected in 2016, and brains from decedents with a dementia diagnosis contained significantly more plastic than age-matched brains without dementia, a correlational finding the authors are careful to distinguish from a causal one [8]. A newer, more sensitive histology-preserving extraction protocol applied to placental tissue from the ENVIRONAGE birth cohort reported concentrations one to several orders of magnitude higher than earlier estimates, in the thousands to millions of particles per cubic centimeter, and found a positive association between microplastic load and γ-H2AX-labeled DNA damage within the same placental tissue [9], directly linking particle burden to a genotoxic endpoint in human tissue rather than only in animal models, though this finding is currently a preprint and awaits peer review. A separate Canadian cohort using Raman micro-spectroscopy detected both microplastics (polyethylene, polypropylene, polystyrene, and polyvinyl chloride) and co-occurring non-plastic particulates (carbon, graphite, and lead oxide) in every placenta examined, underscoring that microplastic exposure in practice co-occurs with, rather than substitutes for, the metal and combustion-particle exposures discussed elsewhere in this review [10].

Two further findings connect this exposure literature more directly to mechanisms and outcomes already established elsewhere in this paper. Ex vivo work in human placental explants, rather than animal or cell-line models, found that polystyrene microplastic exposure produced time-dependent cytotoxicity, oxidative stress, and metabolic alterations directly in human placental tissue [11], providing a direct human-tissue bridge to the oxidative stress mechanisms elaborated in the animal literature below. Separately, a higher placental microplastic burden was associated with altered fetal hormone levels measured in umbilical cord blood, including reduced glucocorticoid hormones and altered androgenic hormone levels [12]. This finding connects plastic particle burden to both the cortisol-mediated stress axis discussed in Section 4 and the androgen/aromatase-mediated mechanism proposed for bisphenol A in Section 2.2. A small case series examining six fetuses from stillbirth or severe malformation cases found microplastics in every brain, heart, lung, liver, kidney, and intestine sampled, with polyethylene accounting for 98.4% of particles identified and the highest particle counts on the placental maternal surface, followed by the heart and liver [13]. Taken together, this human evidence base, while still dominated by small, single-center detection studies rather than large prospective cohorts, has moved substantially beyond the pilot studies that first confirmed placental crossing several years ago, and now includes a genotoxicity correlate, a direct human-tissue oxidative stress finding, and a fetal endocrine correlate that together support, without yet proving the plausibility of the animal-model mechanisms reviewed next.

The single largest particle detected in that series measured 897 µm and was found in the liver. A pairwise correlation analysis across organs found only one statistically robust association after correction for multiple comparisons: placental maternal-surface and fetal lung particle counts were near-perfectly correlated (r = 0.986, FDR-adjusted P = 0.0084), which the authors attribute to fetal hemodynamics, since the fetal lung receives a large fraction of combined ventricular output despite being non-respiratory in utero, making it an early downstream recipient of particles crossing the placenta into fetal circulation. Notably, microplastic burden and distribution did not correlate with the type or severity of fetal malformation, or with fetal death, across these six cases—a null finding the authors read as evidence that microplastic accumulation likely reflects maternal exposure level and individual biology rather than fetal pathology itself, and that too few cases exist yet to treat this as informative about any causal role.

General neurotoxicity reviews describe blood–brain barrier (BBB) penetration, oxidative stress, mitochondrial dysfunction, protein aggregation, and chronic neuroinflammation as consistent findings across in vitro and in vivo MNP studies [14,15]. More specific to fetal neurodevelopment, polystyrene nanoplastics (PS-NPs) are directly neurotoxic to primary neuronal and glial cultures, inducing apoptosis and oxidative stress [16]. In human iPSC-derived cortical spheroids, a three-dimensional model of the fetal cortex, short-term PS-microplastic exposure paradoxically increases proliferation/progenitor gene expression, while longer exposure reduces viability and downregulates the mature neuronal marker and cortical layer-VI marker TBR1, indicating disrupted corticogenesis [17].

Animal studies extend these findings to the intact, developing brain. Maternally ingested PS-NPs reach the offspring’s brain via breast milk, disrupting neural stem cell function and producing sex-specific (female-predominant) cognitive deficits [18]. Gestational and lactational nanoplastic exposure in rats selectively bioaccumulates in the fetal cerebellum and damages myelin development [18], while a separate rat model demonstrates that maternal PS-NP exposure induces P53-mediated ferritinophagy and ferroptosis in the offspring’s hippocampus, producing a measurable decline in learning and memory [19]. P53-mediated ferritinophagy occurs when the tumor suppressor gene p53 triggers the degradation of ferritin to release iron for stress response [20], while ferroptosis is a programmed cell death involving lipid peroxides and is dependent on iron [21].

A non-mammalian model adds convergent, mechanistically granular support for this DNA-damage pathway. In Caenorhabditis elegans exposed to polystyrene nanoplastics at environmentally relevant concentrations (0.1–10 mg/L) from the L1 larval stage, reproductive toxicity, reduced brood size, fewer fertilized eggs and oocytes, impaired gonad development, and increased germline apoptosis were evident in the exposed parental generation and transmitted to the first unexposed offspring generation (F1), but resolved by F2 and F3. Mechanistically, nanoplastic exposure activated the DNA damage checkpoint kinase ATL-1 and the p53 ortholog CEP-1 (mutants of either gene rescued the reproductive phenotype), while simultaneously suppressing the homologous-recombination repair protein RAD-51, indicating that nanoplastic reproductive toxicity arises from a combined mechanism of increased DNA damage signaling and impaired DNA repair capacity, rather than damage alone. The convergence with the p53-mediated ferritinophagy/ferroptosis mechanism described above, in a wholly different model organism and tissue, strengthens the case that DNA-damage-response activation is a conserved, cross-species route from nanoplastic exposure to germline and neurodevelopmental injury, even though this particular study’s transgenerational effects proved reversible by the third generation rather than permanent [22].

A rat model spanning both gestational and lactational exposure windows adds further support and useful nuance: pregnant and lactating rats exposed to polystyrene nanoplastics produced offspring with reduced cortical thickness, disordered neocortical migration (excess superficial-layer neurons and fewer deep-layer neurons), widened hippocampal synaptic clefts with reduced postsynaptic density, and altered cortical monoamine and hippocampal amino acid neurotransmitter profiles. Behaviorally, offspring showed reduced anxiety-like behavior and spatial memory deficits. The effects of exposure during pregnancy were more severe than those during lactation alone, reinforcing the prenatal-window emphasis elsewhere in this review [23].

Most directly relevant to ASD, three recent mouse studies move beyond general neurotoxicity to ASD-specific behavioral endpoints. Maternal polystyrene microplastic exposure impairs social behavior in offspring mice [24]. Neonatal polystyrene nanoplastic exposure impairs microglia-mediated synaptic pruning during early neurodevelopment, via disrupted microglial autophagy and energy metabolism, producing lasting social behavioral defects in adulthood [25], directly implicating the same pruning process we have previously linked to oxidative stress and taurine sufficiency [1]. Maternal PS-NP exposure via drinking water also produces a sex-specific developmental milestone shift (earlier eye-opening in males) and detectable ex vivo MRI brain structure differences in the cortex, hippocampus, striatum, and corpus callosum [26]. Most strikingly, a mouse model using polyethylene (PE) microplastics, the most common of consumer plastics, explicitly tested and confirmed the microplastic–ASD hypothesis: prenatal PE exposure produced impaired social interaction and repetitive behavior, altered brain glucose metabolism and gene expression, and ASD-associated gut microbiome shifts, assessed via PET imaging, MR spectroscopy, and microbiome analysis [27]. A 2025 systematic review focused specifically on micro/nanoplastic-induced endocrine disruption in rodents makes the ASD connection explicit at the review level, synthesizing the mechanistic case for endocrine-mediated MNP neurodevelopmental risk across the rodent literature [28].

#### Speculative Mechanism of Developmental Action of Micro/Nano Plastics

We speculate that, taken together, this literature suggests at least two distinct but potentially convergent developmental mechanisms:

- impaired corticogenesis during the neurogenic window (evidenced by reduced TBR1 expression [17]), and
- disrupted activity-dependent synaptic pruning during the early postnatal window (evidenced by impaired microglial function [25]).

Both processes are established contributors to ASD pathophysiology independent of plastics exposure, raising the possibility that MNPs act as a novel upstream trigger for previously described downstream neurodevelopmental pathways rather than a wholly distinct mechanism.

A companion mouse study from the same laboratory extends the microglial-pruning finding above from social behavior specifically to a broader ADHD-like phenotype: lifelong polystyrene nanoplastic exposure, beginning in utero, produced hyperactivity, increased risk-taking, and impaired motor learning and executive function in adult offspring, together with a lowered epileptic threshold and accelerated indices of brain aging later in life [29], reinforcing that MNP exposure during gestation can durably reprogram attention- and impulse-relevant circuitry rather than producing a transient postnatal effect. Human evidence directly linking plastics exposure to ASD severity, as opposed to preclinical phenotypes or diagnosis risk alone, remains rare; the CHARGE study addressed this gap by scoring parental occupational chemical exposures during the periconceptional and prenatal periods against validated ASD severity and adaptive-skill measures (ADOS-2, Vineland) in 532 parent–child pairs, finding that paternal and maternal occupational exposure to plastics/polymers specifically was associated with greater irritability, hyperactivity, and poorer receptive and visual-reception language, alongside lower daily living skills, with additional signals for occupational phenol and ethylene oxide exposure [30]. This occupational finding is notable both for using a dose-relevant, real-world human exposure route distinct from the dietary/inhalational routes emphasized elsewhere in this section, and for scoring symptom severity rather than diagnosis, complementing the preclinical behavioral phenotype literature above.

### 2.2. Plastic-Associated Endocrine-Disrupting Chemicals

Beyond the physical particle, plastics are manufactured with and leach a range of endocrine-disrupting chemicals (EDCs) that have a more mature human epidemiological literature than the particles themselves. Table S2a–e in the Supplemental Files provide a summary list of each report described in this section, its major findings, and a link to the publication.

In the TIDES pregnancy cohort, a one-quintile increase in an early-pregnancy phthalate metabolite mixture was associated with a higher Social Responsiveness Scale (SRS-2) total score and worse adaptive skills, with a stronger effect in boys [31]. A combined analysis of the HOME and EARLI cohorts using quantile g-computation found smaller, cohort-specific associations between a six-metabolite phthalate mixture and SRS scores, with the EARLI cohort at high familial risk for ASD showing a different direction of effect than the general-population HOME cohort, thus suggesting that population susceptibility modifies the phthalate–ASD relationship [32]. A systematic review and meta-analysis of seven studies on phthalates and social behavior/ASD found the aggregate evidence to be slight and indeterminate, cautioning that exposure misclassification and mixture effects likely obscure true associations [33]. A 2024 mechanistic review proposes that phthalate exposure and micronutrient status, including folate and vitamin B status, jointly disrupt prenatal one-carbon and lipid metabolism as a shared pathway toward suboptimal neurodevelopment, directly bridging the endocrine disruption and nutrient metabolism literatures [34].

Bisphenol A (BPA) shows a similar pattern of modest, sex-stratified associations. A prospective cohort measuring seven phthalate metabolites and BPA found trimester-specific associations with motor, problem-solving, and social developmental scores at 18 months, with free T4 (thyroid hormone) partially mediating the association between low-molecular-weight phthalates and communication scores [35]. In the EDEN mother–child cohort, prenatal BPA was positively associated with the SDQ relationship-problems subscale at age 3 and with hyperactivity–inattention at age 5 in boys, though this outcome measure captures general behavioral difficulty rather than an ASD diagnosis specifically [36].

A considerably stronger and more directly ASD-specific BPA finding comes from a multimodal study combining the Barwon Infant Study (BIS) human birth cohort (*n* = 1074) with parallel mouse and cell culture experiments. In BIS, higher prenatal maternal BPA was associated with greater autism spectrum problems scores at age 2 and with clinician-confirmed ASD diagnosis by DSM-5 criteria at age 9, but only in boys with a genetic profile conferring low brain aromatase enzyme activity (matched OR 6.24 for diagnosis in this subgroup); the population-attributable fraction among these boys was 12.6%. The same study found that prenatal BPA predicted increased methylation of the brain-specific CYP19A1 (aromatase) gene promoter in cord blood, replicated in an independent cohort, and showed in mice that mid-gestation BPA exposure suppresses brain aromatase, reduces amygdala neuron number and activity, and produces male-specific ASD-like social and repetitive-behavior deficits, mirroring the phenotype of aromatase-knockout mice. Postnatal treatment with an estrogenic compound (10HDA) reversed these deficits. This is one of the few studies in the plastics-EDC literature to combine a genetically stratified human ASD-diagnosis outcome with a fully worked mechanistic pathway (BPA to aromatase suppression to amygdala dysfunction to male-specific ASD-like behavior) and a candidate reversibility intervention, making it a strong anchor point for the convergent-mechanisms discussion in Section 3 [37].

Organophosphate esters (OPEs), used as flame retardants and plasticizers in place of phased-out brominated compounds, show one of the more robust recent signals in this literature. A 15-cohort analysis from the NIH ECHO consortium (n = 4159 mother–child pairs) found that high BBOEP exposure was associated with both a higher autistic trait score and higher odds of an autism diagnosis, with a stronger effect in males. A related metabolite, BCPP, showed a similar dose-dependent association [38]. A separate, non-neurodevelopmental ECHO analysis of the same OPE exposure class (5087 individuals, 14 cohorts) found that gestational DBUP/DIBP was associated with increased childhood obesity risk and BDCPP with a paradoxical inverse association. While the outcome is metabolic rather than neurodevelopmental, it independently corroborates that this OPE panel produces measurable, metabolite-specific, and sometimes divergent developmental consequences at the population scale, consistent with the endocrine-mediated mechanism proposed above [39]. A large cohort-only meta-analysis discussed further in Section 2.5 independently corroborates two specific phthalate metabolites, mono-3-carboxypropyl phthalate and monobutyl phthalate, as significantly associated with ASD across the pooled cohort literature [40].

A separate, larger ECHO consortium analysis spanning 13 cohorts examined prenatal urinary metabolites of 27 phthalates and 6 alternative (non-phthalate) plasticizers against internalizing and externalizing behavior in early childhood general behavioral domains, not an ASD diagnosis or ASD-specific trait measure. It found that two phthalate metabolites, MBzP and MHxP, were associated with higher externalizing scores, that other associations were inconsistent across cohorts, and that the phthalate-replacement plasticizer DINCH showed no association with either behavioral domain, with child sex modifying the direction of several externalizing associations [41]. The null finding for DINCH is notable given its increasing use as a phthalate substitute, suggesting that regrettable substitution, replacing one neurodevelopmental toxicant with a mechanistically similar one, may not apply uniformly across all alternative plasticizers.

A study in the MARBLES cohort (178 mother–child pairs with elevated familial ASD likelihood) adds an important null-leaning data point to this mixed picture: across 14 phthalate metabolites measured in second- and third-trimester maternal urine, overall phthalate biomarker concentrations, both individually and as a mixture, were not associated with Child Behavior Checklist scores at preschool age. In exploratory, non-FDR-surviving analyses, three metabolites (monoisobutyl phthalate, monohydroxy-isobutyl phthalate, and monobenzyl phthalate) were inversely associated with anxious/depressed symptoms or somatic complaints, while two second-trimester-specific metabolites (mono(3-carboxypropyl) phthalate and monocarboxyisononyl phthalate) were positively associated with somatic complaints. All associations became non-significant after false discovery rate correction, and the authors’ stated conclusion was that the study found no clear evidence of an effect. The second-trimester-specific positive signal was nonetheless proposed, cautiously, as a possible sensitive window worth further study [42]. Read alongside the positive TIDES and HOME/EARLI findings above, this MARBLES null result, in a cohort selected specifically for elevated ASD familial risk, where a true effect might be expected to be most detectable, reinforces the broader pattern in this literature: phthalate–behavior associations are inconsistent across cohorts and exposure-assessment strategies, likely reflecting genuine effect heterogeneity, exposure misclassification, or both, rather than a uniformly robust signal.

Several further MARBLES and ECHO analyses extend the OPE and broader EDC picture developed above. A dedicated OPE–ASD analysis in 277 MARBLES mother–child pairs found no clear evidence that gestational OPE exposure was associated with ASD risk overall, though homeownership status may modify the association; a subsequent corrigendum made no change to the paper’s substantive conclusions [43]. A separate, larger ECHO Cohort analysis (2948 mother–child dyads, 12 cohorts, nine OPE biomarkers assessed against CBCL/1½–5 composite scores) examined the same OPE exposure class against general early-childhood behavioral outcomes rather than ASD diagnosis specifically, finding that low bis(butoxyethyl) phosphate exposure was associated with higher T-scores across all three composite scales (internalizing, externalizing, and total problems), that both low and high bis(1-chloro-2-propyl) phosphate exposure were associated with higher externalizing scores specifically, and, in a directionally unexpected result, that detectable dipropyl phosphate was associated with lower externalizing scores; associations for bis(1,3-dichloro-2-propyl) phosphate and high bis(1-chloro-2-propyl) phosphate exposure were stronger in males, and the bis(1-chloro-2-propyl) phosphate finding was further amplified among children from highly socially vulnerable neighborhoods [44]. Extending the exposure window beyond gestation, a MARBLES study measured 32 EDC biomarkers, including phthalates, phenols, and parabens, directly in infant urine at 3 and/or 6 months of age against later ASD, non-typical development, and cognitive outcomes; this is one of the few analyses in this literature to examine a postnatal rather than purely prenatal exposure window [45]. Two additional ECHO analyses probe mechanisms and a related cognitive endpoint: prenatal BDCPP (a TDCPP metabolite) was associated with elevated maternal interleukin-8, an inflammatory marker, pooled across three ECHO sites, offering one candidate immune-mediated pathway from OPE exposure to downstream child outcomes [46]; and in an 831-pair ECHO cohort, prenatal diphenyl phosphate (DPHP, detected in over 96% of samples) was associated with modestly lower child cognitive scores on standardized IQ testing, while several other OPE metabolites were associated with higher cognition scores predominantly in boys (general cognitive/IQ testing, not an ASD diagnosis or ASD-specific trait measure) [47].

#### Speculative Mechanism of Developmental Action of Endocrine Disrupting Chemicals

The EDC literature, in contrast to the direct-particle literature in Section 2.1, points most consistently toward endocrine (particularly thyroid hormone) disruption during the second and third trimesters. This is a period of intense fetal brain thyroid hormone dependence for myelination and neuronal migration. The EDC literature also shows a secondary, mechanistically distinct contribution via one-carbon metabolism interference that would be expected to interact with maternal folate/Vitamin B status [34,35]. Mixture-modeling approaches developed for this literature, such as those applied to prenatal phthalate, paraben, and phenol co-exposure in the CHAMACOS cohort, illustrate the broader methodological point that these compounds rarely occur in isolation; that particular study examined childhood adiposity rather than a neurodevelopmental outcome, but the same weighted-quantile-sum mixture framework has since been applied to ASD-relevant endpoints elsewhere in this review [32,48].

### 2.3. Ambient Air Pollution

Air pollution has the largest and most consistent epidemiological evidence base of any pollutant class reviewed here. Table S3a,b in the Supplemental Files provide a summary list of each report described in this section, its major findings, and links to the publications.

A 2025 meta-analysis of 27 studies (n = 369,460, with 47,973 ASD cases) found that gestational PM2.5 exposure is associated with a 15% increase in overall ASD risk, with the largest effect occurring in the third trimester, and postnatal year-2 exposure carrying the highest risk of any window examined [49]. A separate 2024 meta-analysis of 14 studies found PM2.5 significantly associated with ASD, with a weaker, non-significant signal for NO_2_ [50]. An earlier narrative review identified prenatal PM2.5 as the most consistently replicated air pollution signal for ASD across the literature to that point [51].

A substantial and likely underappreciated fraction of ambient PM2.5, particularly near roadways, originates from tire wear rather than combustion. Section 2.4 addresses this source and its degradation products directly, since the evidence base for tire-derived particles has matured enough to warrant separate treatment from air pollution’s combustion-derived fraction.

Two very large, methodologically rigorous cohort studies published in 2025–2026 refine this picture by moving from PM2.5 mass to PM2.5 chemical composition and gaseous co-pollutants. A 2.18-million-birth Ontario, Canada, cohort found that sulfate and ammonium, specific PM2.5 components, not PM2.5 mass overall, were independently associated with ASD, with the second and third trimesters (gestational weeks 21–36) representing the sensitive exposure window; postnatal ozone exposure during the first year of life was independently associated as well [52]. A 1.44-million-birth South Korean nationwide cohort (linking National Health Insurance Service birth records to insurance claims diagnoses, with follow-up to 13 years) found that each 0.01 ppm increase in prenatal NO_2_ was associated with an 18% higher risk of long-term neurodevelopmental disorders (aOR 1.18, 95% CI 1.15–1.20), and each 0.001 ppm increase in SO_2_ with a 1% increase in risk (aOR 1.01, 95% CI 1.01–1.02). Associations held consistently across intellectual disability, developmental delay, and behavioral/emotional disorder subtypes, and were robust after excluding children who died during follow-up and across three separate follow-up windows (0–4, 5–9, 10–13 years post-birth) [53]. The divergence between which specific pollutant component predominates in each cohort—sulfate/ammonium in Ontario versus NO_2_/SO_2_ in South Korea—suggests either population-specific pollutant source mixtures (e.g., differing contributions from coal combustion, marine shipping fuel, or vehicular traffic) or multiple, independently sufficient mechanistic pathways converging on the same neurodevelopmental outcome. As we have argued in a commentary on the South Korean cohort, these pollutant-level associations are best conceptualized not as single sufficient causes but as risk modifiers that interact with genetic variants, maternal immune and metabolic status, and micronutrient sufficiency, particularly one-carbon metabolism, to shift the probability of a neurodevelopmental outcome rather than determine it outright [54]. A mechanistic network-toxicology analysis modeling long-term PM2.5 exposure against ASD symptom severity, rather than diagnosis risk, independently supports a neuroinflammatory pathway, implicating microglial activation and impaired synaptic plasticity as the proximate route from chronic particulate exposure to symptom exacerbation [55]. A broader review of prenatal PM exposure across the neuropsychiatric spectrum—ASD, ADHD, anxiety, depression, and schizophrenia—situates the ASD-specific findings above within this wider set of outcomes and mechanisms, reinforcing that PM2.5/PM10 neurotoxicity is not ASD-selective but rather reflects a shared vulnerability of the developing brain to oxidative and inflammatory insult [56].

The consistent identification of second/third-trimester sensitivity windows across both the meta-analytic and large-cohort literature points toward late-gestational processes: late neurogenesis, oligodendrocyte proliferation, and the onset of myelination as the most plausible target, with a secondary postnatal year-1 window (ozone) implicating early synaptic pruning and microglial maturation, mechanistically parallel to the microplastic findings in Section 2.1 [25,52].

A third large cohort adds a further layer of pollutant-component specificity. The CHARGE case-control study (1281 children, 751 ASD cases) examined size-resolved PM components down to the ultrafine fraction (PM0.1) for the first time in this literature and found that first-year PM0.1 iron, manganese, black carbon, and sodium were consistently associated with higher ASD odds (e.g., iron OR 1.60, 95% CI 1.21–2.12; sodium OR 1.92, 95% CI 1.24–2.99), with weaker evidence for prenatal-window exposures [57]. That three large, independent cohorts (Ontario, South Korea, and California) each identify a different leading pollutant component—sulfate/ammonium, NO_2_/SO_2_, and iron/manganese/black carbon/sodium, respectively—reinforces the possibility raised above that source-specific mixtures, rather than a single causal pollutant, underlie the broader PM2.5–ASD association and strengthens the case in Section 2.4 that metal-bearing particles from combustion and abrasion sources (including tire and brake wear) deserve component-level scrutiny rather than being treated as an undifferentiated PM2.5 mass.

A fourth large cohort draws on the full ECHO Cohort Consortium rather than a single regional dataset: 8035 mother–child pairs across 44 United States cohorts, with autism assessed both dimensionally (parent-rated Social Responsiveness Scale) and categorically (physician-diagnosed ASD). Prenatal PM2.5, NO_2_, and ozone were each associated with autism-related traits and diagnosis, and these associations were evident even at the comparatively low ambient pollutant levels typical of current US regulatory limits, arguing against any clear no-effect threshold for prenatal air pollution exposure [58]. A related ECHO analysis, examining gestational exposure to wildfire smoke PM2.5 rather than routine ambient PM2.5, found measurable shifts in maternal circulating oxylipins, a class of bioactive, inflammation-related lipid mediators, up to 13 months after a discrete wildfire exposure event, though co-measured flame retardants were unaffected. This adds a plausible peripheral-inflammatory mechanism linking acute, high-intensity smoke exposure of the kind discussed above to the oxidative/inflammatory final-common-pathway framework developed in Section 3 [59]. A related but larger ECHO-wide analysis (20,034 births, 30 sites) found that wildfire-specific PM2.5, distinct from routine ambient PM2.5, was associated with an increased risk of preterm birth. While the outcome is obstetric rather than neurodevelopmental, it complements the wildfire-ASD findings above by establishing that this specific exposure source carries measurable developmental consequences through an independent pathway [60].

A source-specific analysis of a different kind comes from a 2026 California study of 8.6 million births, the largest wildfire smoke–ASD analysis to date. Averaged across the whole cohort, wildfire-related PM2.5 exposure showed weak or null associations with ASD in single-pollutant models, but this masked striking effect modification by background air pollution: among children in the lowest quartile of background (non-wildfire) PM2.5, the highest wildfire-derived PM2.5 exposure category (>90th percentile) was associated with a 50% increase in ASD odds (OR 1.50, 95% CI 1.44–1.57), an association that weakened progressively across background-pollution quartiles and reversed to null or negative in the highest-background-pollution quartile. The authors attributed this pattern partly to possible live-birth bias in chronically high-pollution areas. Associations were also markedly stronger in non-metropolitan areas (OR 1.71 at the >90th percentile) than in metropolitan areas, and stronger in births after 2014, coinciding with DSM-5 diagnostic criteria adoption. A wildland–urban interface (WUI) source tracer, capturing smoke from structure and infrastructure fires rather than pure vegetation burning, showed a dose-dependent association at higher exposure percentiles that held even in metro areas and across all racial/ethnic and socioeconomic groups, suggesting a uniquely toxic combustion signature [61]. This suggests that wildfire smoke, an increasingly prevalent and climate-sensitive PM2.5 source, contributes a distinct, intensity- and context-dependent risk on top of routine ambient air pollution, rather than being interchangeable with it.

A companion multi-site cohort study, using data from 8370 mother–child pairs across 28 ECHO Cohort sites, examined general behavioral outcomes (Child Behavior Checklist internalizing/externalizing scores) rather than ASD diagnosis directly, but adds valuable trimester-specific granularity: first-trimester PM2.5 was associated with externalizing behavior, second-trimester PM2.5 with internalizing behavior, and third-trimester NO_2_ with both, while O_3_ showed no overall association [62]. Associations for NO_2_ were strongest among children in the lowest-opportunity neighborhoods, echoing the socioeconomic effect-modification pattern seen in the Ontario cohort above [52]. As with the Philippat BPA findings in Section 2.2, this study’s outcome measure is general behavioral difficulty rather than an ASD diagnosis, so it should be read as supportive rather than direct evidence. The cohort-only meta-analysis discussed in Section 2.5 independently corroborates the gaseous pollutant signal at a pooled level, finding significant associations for NO_2_ (RR 1.20) and, at the subgroup level, for carbon monoxide (RR 1.57) and nitrogen oxides generally (RR 1.09) [40]. A methodologically distinct New York City cohort (470 mother–child pairs) used a distributed-lag model to identify week-by-week windows of susceptibility rather than trimester-level exposure, finding that first-trimester NO_2_ (approximately gestational weeks 29–30) and second-trimester PM2.5 (weeks 17–18) were each independently, inversely associated with cognitive scores at ages 1 and 3 (a general cognitive development measure, not an ASD-specific outcome), with no association at age 2, a pattern the authors attribute to the coarser resolution of trimester-based exposure windows obscuring narrower, week-specific sensitive periods [63]. This finer-grained approach converges with, and adds temporal precision to, the second/third-trimester and postnatal year-1 windows identified by the larger cohorts above.

This section has so far focused on outdoor ambient air pollution; a large ECHO Cohort analysis extends the scope to common indoor household exposures, which can independently contribute to indoor air pollution and damp conditions. Across 11,483 mother-infant dyads from 31 ECHO sites, the self-reported presence of a gas stove, mold/mildew, or water damage in the home during pregnancy (53%, 13%, and 15% prevalence, respectively) measured gestational duration and fetal growth, obstetric rather than neurodevelopmental outcomes, and was not consistently associated with gestational duration or fetal growth overall. Effect modification was detected at the subgroup level: mold/mildew was associated with somewhat higher odds of early-term or preterm birth in newer homes and among mothers with higher educational attainment, while water damage was inversely associated with preterm birth specifically among non-Hispanic white participants. As with several other null or mixed findings in this review, we read this as informative rather than as evidence of no effect: indoor combustion and dampness exposures are plausible contributors to the same oxidative/inflammatory pathways implicated throughout this section, but this study measured only presence/absence of each condition rather than exposure intensity, ventilation, or duration, and measured birth outcomes rather than neurodevelopment specifically, so a true effect on ASD risk specifically cannot be ruled out by this design [64].

**Figure 3:**
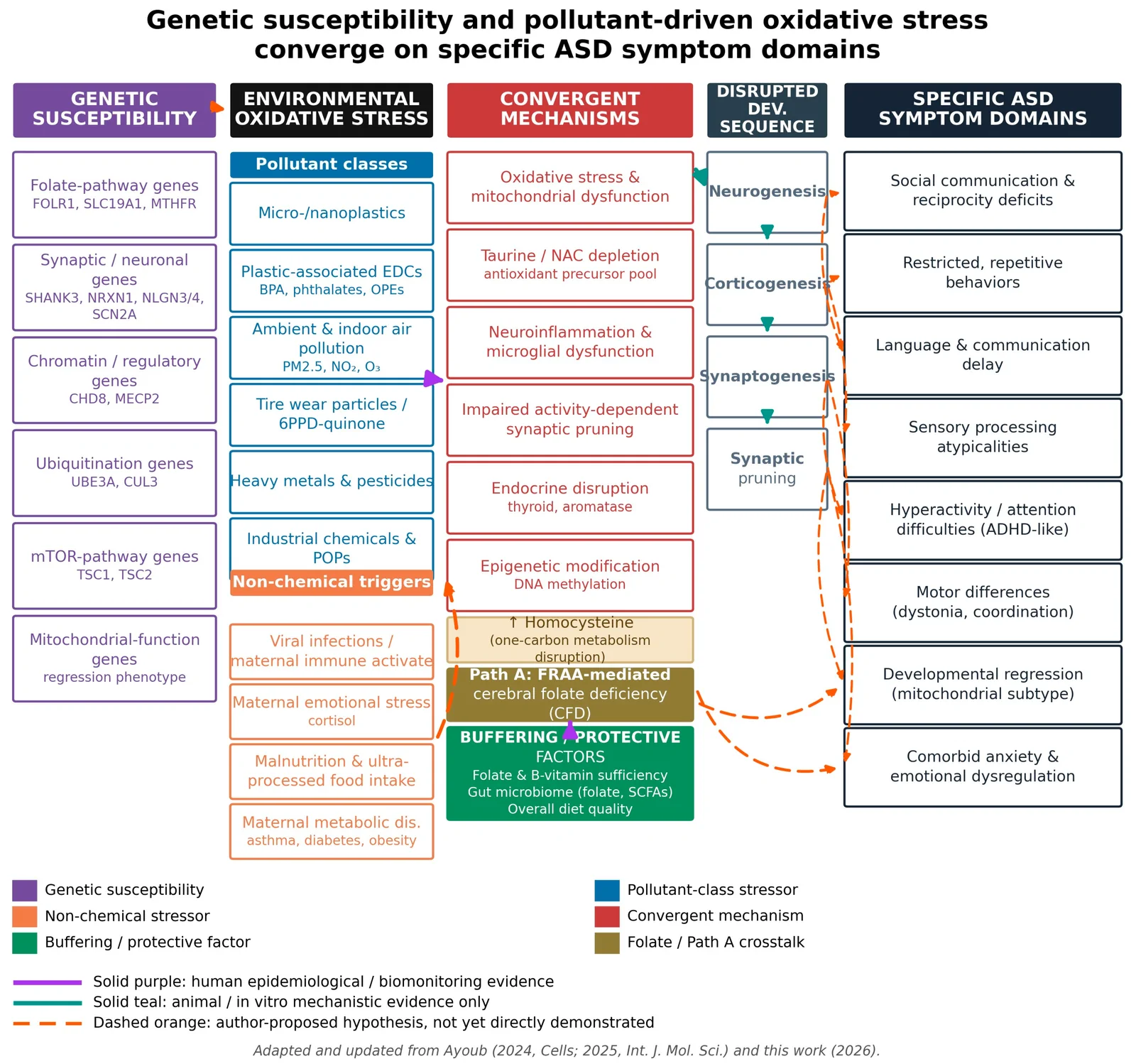
Genetic susceptibility and pollutant-driven oxidative stress converge on specific ASD symptom domains. This schematic illustrates our revised, proposed multifactorial model, integrating genetic, environmental, and metabolic contributors to autism spectrum disorder (ASD) risk. Environmental oxidative stressors (pollutant and non-chemical) deplete taurine/NAC and elevate homocysteine, cross-communicating with the folate/FRAA pathway. Genetic susceptibility and folate/microbiome buffering are proposed, though not yet established, to jointly shape which developmental process, and which resulting domain, is disrupted. Genetic susceptibility factors (purple), including variants in the folate pathway, synaptic, chromatin-regulatory, ubiquitination, mTOR pathway, and mitochondrial function genes, act as a predisposing layer that both lowers the threshold for environmentally triggered convergent mechanisms (dashed arrow) and directly influences which symptom domain ultimately predominates (dashed arrow, bottom). Environmental oxidative stressors are grouped into pollutant classes (blue), including micro-/nanoplastics, plastic-associated endocrine-disrupting chemicals (BPA, phthalates, organophosphate esters), ambient and indoor air pollution, tire-wear particles/6PPD-quinone, heavy metals, pesticides, and industrial chemicals/persistent organic pollutants, as well as non-chemical triggers (coral), including maternal immune activation, maternal stress, malnutrition/ultra-processed food intake, and maternal metabolic disease. Both stressor categories converge on a shared set of mechanisms (red), namely oxidative stress and mitochondrial dysfunction, depletion of the taurine/N-acetylcysteine (NAC) antioxidant precursor pool, neuroinflammation and microglial dysfunction, impaired activity-dependent synaptic pruning, endocrine disruption, and epigenetic (DNA methylation) changes. Oxidative stress-associated elevation of homocysteine links this convergent mechanism set to folate receptor-α autoantibody (FRAA)-mediated cerebral folate deficiency (CFD, olive), reflecting the one-carbon metabolism crosstalk between pollutant-driven oxidative stress and folate transport across the blood–cerebrospinal fluid barrier. Buffering/protective factors (green), including folate and vitamin B sufficiency, a folate- and short-chain fatty acid-producing gut microbiome, and overall diet quality, are proposed to offset this convergent mechanism burden—a hypothesis this review develops but does not consider established, as indicated by the blunt-ended (inhibitory-style) arrow. These convergent mechanisms disrupt the normal sequence of neurodevelopment (neurogenesis → corticogenesis → synaptogenesis → synaptic pruning, grey), which in turn gives rise to the specific, clinically recognized ASD symptom domains shown at right (dark grey), including deficits in social communication and reciprocity, restricted/repetitive behaviors, language and communication delays, sensory processing atypicalities, hyperactivity/attention difficulties, motor differences, developmental regression (associated with the mitochondrial-dysfunction subtype), and comorbid anxiety/emotional dysregulation. Solid black arrows denote associations supported by human epidemiological or biomonitoring data; solid gray arrows denote mechanistic links demonstrated in animal or in vitro models without a corresponding human ASD-outcome study; dashed arrows denote modulatory, susceptibility-conferring, or cross-pathway relationships that remain, at this stage, author-proposed hypotheses rather than demonstrated pathways. This figure depicts a conceptual integration of convergent mechanisms, not a validated sequential causal chain from any single exposure to ASD diagnosis. The biological responses shown here (oxidative stress, neuroinflammation, mitochondrial dysfunction, endocrine disruption, epigenetic change) are nonspecific and implicated in many neurological and systemic conditions, and their recurrence across exposures does not by itself establish ASD-specific mechanistic convergence. The figure artwork itself has been drawn to use this three-category line convention (solid black, solid gray, dashed) rather than a single undifferentiated arrow style [1,2].

A newly published prospective cohort study provides the first direct human evidence that ambient air pollution is associated with maternal folate pathway biomarkers, rather than folate status alone [65]. In the Maternal-Infant Research on Environmental Chemicals (MIREC) cohort (n = 838 with complete third-trimester data), a Bayesian weighted quantile sum regression mixture of NO_2_, O_3_, PM2.5, and SO_2_ was positively associated with third-trimester plasma total folate and with the proportion of unmetabolized folic acid (UMFA), and negatively associated with the proportion of the biologically active vitamer 5-methyltetrahydrofolate (5MTHF); no association was observed in the first trimester. O_3_ and PM2.5 were the dominant drivers of the mixture effect, and associations were stronger among women carrying female fetuses. This is, to our knowledge, the first study to demonstrate that ambient air pollution measurably shifts maternal folate metabolism toward the unmetabolized, less bioactive form, which is directly relevant to the folate pathway crosstalk hypothesis developed in Section 7 of this review: it provides human exposure/biomonitoring evidence for a pollutant–folate interaction, not evidence of an ASD outcome, and we grade it accordingly. The authors note that the mechanism is not yet established and propose several candidates, including pollutant-driven changes in folate-metabolizing enzyme activity or hepatic conversion capacity; they also note that all participants remained above the folate threshold associated with maximal neural-tube defect risk reduction, so these findings speak to subclinical shifts in folate handling rather than deficiency. We flag this study as an important, though still single-cohort and exploratory, empirical anchor for the folate-pollutant crosstalk arrow in Figure 3, and recommend replication before treating the effect as established.

#### Speculative Mechanism of Developmental Action of Air Pollution

We speculate that the convergent evidence for gaseous co-pollutants (NO_2_, SO_2_, ozone) and metal-bearing PM components (iron, manganese, black carbon) across independent cohorts points to at least two overlapping pathways rather than a single PM2.5 mechanism. The first is direct fetal oxidative stress: redox-active transition metals adsorbed onto ultrafine particles cross the placenta and blood–brain barrier and can catalyze reactive oxygen species generation directly in fetal neural tissue, consistent with the PM0.1 metal-component findings above [57]. The second is a maternal systemic inflammatory route, in which gaseous pollutants and PM2.5 mass provoke maternal airway and systemic inflammation, with downstream cytokine and oxylipin signaling crossing the placenta to disrupt fetal neurogenesis and myelination without requiring direct fetal particle deposition, consistent with the wildfire-associated oxylipin shifts discussed above [59]. The consistent second/third-trimester and postnatal year-1 sensitivity windows identified across cohorts in this section map onto late neurogenesis and oligodendrocyte proliferation, and early synaptic pruning, respectively, mechanistically parallel to the microplastic-driven pruning disruption proposed in Section 2.1 [25]. We further speculate that the population-specific divergence in which pollutant component predominates—sulfate/ammonium, NO_2_/SO_2_, or iron/manganese/black carbon/sodium—across the cohorts reviewed above reflects genuinely distinct, source-specific mixtures converging on a shared oxidative/inflammatory final common pathway (elaborated in Section 3) rather than a single universally causal component. This distinction matters for prevention: it implies that reducing any one dominant local source, whether traffic-related tire/brake wear, coal combustion, or marine shipping fuel, could meaningfully reduce risk within that specific population, even without a single, universal pollutant-component ranking applicable everywhere.

### 2.4. Tire Wear Particles: A Convergent Micro/Nanoplastic and Air Pollution Source

Tire wear particles (TWPs, also called tire and road wear particles) form through mechanical abrasion between tire and pavement and are now recognized as one of the dominant sources of environmental microplastics, sitting mechanistically at the intersection of Section 2.1 and Section 2.3. Table S4 in the Supplemental Files provides a summary list of each report described in this section, its major findings, and a link to the publication.

Tires are manufactured from a complex matrix that generally includes styrene-butadiene rubber, natural rubber, carbon black, silica, and sulfur/zinc-based vulcanization agents. A 2023 comprehensive review of tire wear particle formation, measurement, and physicochemical properties confirms that TWPs commonly carry embedded heavy metals and mineral residue contributed by road-surface abrasion [66]. Beyond the particle itself, tires are formulated with the antioxidant 6PPD (N-(1,3-dimethylbutyl)-N′-phenyl-p-phenylenediamine), which reacts with atmospheric ozone to form 6PPD-quinone (6PPD-Q), a transformation product responsible for acute mass die-offs in coho salmon and increasingly implicated in mammalian developmental toxicity [67].

The strongest tire-specific evidence for a fetal pathway centers on 6PPD-Q’s ability to cross the placenta. In pregnant mice dosed across the period of major organogenesis, 6PPD-Q reached concentrations 1.5–8 times higher than its parent compound 6PPD in the placenta, embryo body, and, critically, embryo brain, and was shown to activate the retinoic acid receptor α (RARα) and retinoid X receptor α (RXRα)—nuclear receptor pathways with established roles in neurodevelopmental gene regulation [67]. This is, to our knowledge, the most direct demonstration in this entire review that a tire-derived chemical reaches the fetal brain during gestation.

Reproductive and endocrine toxicity findings reinforce this placental-transfer signal. Chronic 6PPD-Q exposure in male mice reduced testosterone, impaired sperm quality and in vitro fertilization outcomes, and produced transcriptomic/metabolomic changes enriched in spermatogenesis, apoptosis, arginine biosynthesis, and sphingolipid metabolism in testicular tissue [68]. In a human trophoblast–endometrial coculture model using BeWo spheroids and Ishikawa cells as surrogates for the blastocyst and endometrial epithelium, both 6PPD and 6PPD-Q reduced trophoblast attachment and invasion and downregulated endometrial receptivity markers. This is the first direct evidence that these compounds may impair embryo implantation itself, upstream of any subsequent fetal exposure [69]. Cross-species convergence strengthens the case further: pulsed embryonic 6PPD-Q exposure in coho salmon produced dose-dependent mortality (as low as 21% survival to hatching at the highest tested concentration) with transcriptional disruption of vascular permeability pathways [70], indicating that 6PPD-Q’s developmental toxicity is not confined to a single taxon or organ system.

Because TWPs are classified as a major environmental microplastic source, the broader prenatal microplastics literature reviewed in Section 2.1 is directly relevant here as well. A 2025 comprehensive review concludes that MPs can cross the placental barrier, impair placental function, and alter fetal growth, and specifically documents reduced offspring levels of dopamine, serotonin, acetylcholine, GABA, and oxytocin, accompanied by anxiety-like behavior, social deficits, and cognitive impairment [71]. A companion scoping review on child health confirms the detection of MPs in the placenta, amniotic fluid, umbilical cord, fetal membranes, and umbilical vein blood, with continued postnatal exposure via breast milk [72]. A further review focused on placental penetration documents MP-induced cytotoxicity and genotoxicity in placental cells, as well as altered gestation time, offspring weight, and litter number in mouse models [73]. Additionally, the p53-mediated ferroptosis mechanism, already described for generic PS-nanoplastics in Section 2.1 [19], provides one plausible molecular route by which tire-derived nanoplastic fragments, and not only their 6PPD-Q additive, could independently reach and damage the fetal brain.

#### Speculative Mechanism of Developmental Action of Tire Particles

We propose that tire wear particles constitute a dual-pathway exposure, contributing both a particulate microplastic burden (mechanistically continuous with Section 2.1) and a distinct, chemically active leachate (6PPD-Q) that independently crosses the placenta and acts through nuclear receptor signaling. We further speculate that the well-established prenatal PM2.5–ASD association reviewed in Section 2.3 may be partially confounded by, or mechanistically overlapping with, roadway-proximity tire-particle exposure, since both share vehicular traffic as a common source and both peak in the same urban, high traffic density settings identified as higher risk in the large air pollution cohorts mentioned above [52]. This hypothesis is testable: cohorts with source-apportioned PM2.5 data or direct 6PPD-Q biomonitoring could determine whether the sulfate/ammonium and NO_2_/SO_2_ signals identified in Section 2.3 are independent of, or partially explained by, tire-derived contributions.

### 2.5. Heavy Metals and Pesticides

The literature on metals/pesticides is older and more heterogeneous than the pollutant classes discussed above, reflecting decades of accumulated case-control and cohort work. A comprehensive narrative review of environmental chemical exposures, including metals, pesticides, air pollutants, and solvents, across the growing epidemiological literature found consistent, if individually modest, associations with ASD risk [19]. A systematic review found that 92% of 37 reviewed studies detected an association between estimated environmental toxicant exposure (metals, pesticides, air pollutants, PCBs) and ASD risk, and also reviewed toxicant-biomarker and gene–environment interaction studies implicating detoxification-gene polymorphisms (PON1, GSTM1/GSTP1) as individual susceptibility modifiers [74]. Table S5 in the Supplemental Files provides a summary list of each report described in this section, its major findings, and a link to the publication.

Mercury has the most targeted recent evidence within this class: a 2023 PRISMA-2020 systematic review and meta-analysis of mercury body-burden biomarkers across hair, whole blood, plasma, red blood cells, and urine found that children with ASD show a reduced capacity to eliminate mercury compared to neurotypical children, supporting mercury as a candidate environmental contributor alongside the plastics- and metals-related exposures reviewed elsewhere in this report [75]. Much of this biomarker literature rests on infrastructure built by population-based case-control studies, such as the Early Markers for Autism (EMA) study, which archived prenatal and neonatal biospecimens from California births specifically to enable this kind of retrospective toxicant-biomarker analysis; its cohort profile documents the biologic plausibility rationale that underlies the metals/pesticides evidence base as a whole [76].

The largest quantitative synthesis in this literature to date is a 2024 systematic review and meta-analysis restricted exclusively to cohort-design studies (27 studies, 1289,183 participants, 129 distinct pollutants). This is a methodological strength, since it avoids blending cohort, case-control, and cross-sectional designs the way several earlier reviews did. It found significant individual associations between ASD and nitrogen dioxide (RR 1.20, 95% CI 1.03–1.38), copper (RR 1.08, 95% CI 1.03–1.13), mono-3-carboxypropyl phthalate (β = 0.45, 95% CI 0.20–0.70), monobutyl phthalate (β = 0.43, 95% CI 0.13–0.73), and PCB-138 (RR 1.84, 95% CI 1.14–2.96), as well as subgroup-level associations for carbon monoxide (RR 1.57, 95% CI 1.25–1.97), nitrogen oxides (RR 1.09, 95% CI 1.04–1.15), and a metals subgroup driven by iron and molybdenum (RR 1.13, 95% CI 1.01–1.27). It also found an inverse subgroup association for organophosphates/carbamates (β = -0.49), and, notably, the authors rate overall evidence certainty as low to very low across every analysis once between-study heterogeneity and publication-bias risk are accounted for. Indeed, we carry their caution into our own synthesis in Section 3. Because its significant findings span metals (copper, the iron/molybdenum subgroup), phthalates (MCPP, MBP), and gaseous/particulate air pollutants (NO_2_, CO, NOx) in a single coherent analysis, this study is cited here as well as cross-referenced from Section 2.2 and Section 2.3 [40].

Two recent CHARGE-study analyses add direct, ASD-specific pesticide evidence to this literature. Professionally applied household insecticides during pregnancy and early life were associated with roughly double the odds of ASD, with more frequent application associated with higher odds across most application types [77]. In the same cohort, flea and tick control products, applied indoors, outdoors, or directly on pets, were associated with lower cognitive and adaptive behavior developmental quotient scores among children with ASD or developmental delay [78], extending the pesticide-exposure evidence beyond agricultural and occupational routes to a common household consumer product. A separate, not-yet-peer-reviewed multicenter analysis takes a different approach entirely: rather than testing a single exposure class, it uses machine learning analysis of elemental biodynamics in hair, including the toxic metals lead, manganese, and arsenic discussed above, as an ASD rule-out diagnostic, reporting an AUC of 0.79 and 96% sensitivity. We flag this as an unreviewed preprint, methodologically distinct from the etiological studies mentioned above, and note it here as a biomarker-based application of the same metals panel rather than as confirmatory etiological evidence [79].

Herbicide exposure adds a further dimension to this evidence base. A 2026 analysis from the NYU Children’s Health and Environmental Study (n = 1450) found that maternal urinary glyphosate concentrations measured at 18–25 weeks gestation, but not earlier in pregnancy, were associated with increased odds of preterm birth, an effect concentrated in the spontaneous rather than the medically indicated subtype. As with the wildfire-specific PM2.5 finding above, the outcome here is obstetric rather than neurodevelopmental, but the study is consistent with two smaller prior cohorts, the Puerto Rico PROTECT study and the multi-state TIDES study, in identifying mid-to-late gestation as a sensitive window for glyphosate’s reproductive effects. It extends the metals/pesticides literature reviewed above beyond insecticides to a mechanistically distinct herbicide class with proposed endocrine-disrupting and oxidative stress pathways [80].

#### Speculative Mechanism of Developmental Action of Heavy Metals

The detoxification gene–environment interaction findings [74] suggest that, unlike the plastics and air pollution classes discussed above, where dose and timing appear to be the dominant variables, individual genetic capacity for xenobiotic clearance may be the primary determinant of vulnerability to metals/pesticide exposure. This is mechanistically analogous to the MTHFR-polymorphism-mediated variability in folate metabolism we described previously [1], and raises the possibility of a shared genetic susceptibility architecture across pollutant classes.

### 2.6. Industrial Chemicals: The Foundational Framework

The developmental neurotoxicant paradigm underlying this review originates from Grandjean and Landrigan’s 2006 identification of lead, methylmercury, PCBs, arsenic, and toluene as the first proven human developmental neurotoxicants [5]. The list was updated in 2014 to include manganese, fluoride, chlorpyrifos, DDT, tetrachloroethylene, and PBDEs. They explicitly frame the concurrent rise of ASD, ADHD, and dyslexia within a “pandemic of developmental neurotoxicity” [6]. We propose micro/nanoplastics, tire-derived 6PPD-Q, and their associated endocrine-disrupting chemicals (Section 2.1, Section 2.2 and Section 2.4) as a contemporary, 2020s-era extension of this same list, sharing the core feature that defined the original: ubiquitous, difficult-to-avoid exposure during a developmentally sensitive prenatal and early postnatal critical period window. Table S6a–c in the Supplemental Files provide a summary list of each report described in this section, its major findings, and a link to the publication.

Melamine and its structurally related aromatic amines represent a further, less-studied addition to this industrial–chemical framework. An ECHO Cohort analysis of gestational urinary melamine, melamine analogues, and aromatic amines (n = 1231) found detectable exposure to be near-universal and identified associations with gestational duration and fetal growth, though the study did not assess neurodevelopmental outcomes directly [81]. Aromatic amines are also a structural degradation product of tire-derived 6PPD (Section 2.4), so this exposure class is mechanistically and chemically continuous with, rather than independent of, the tire wear particle pathway discussed above, and may represent an underappreciated shared route of prenatal aromatic amine exposure worth tracking alongside 6PPD-quinone in future biomonitoring work.

Persistent organic pollutants (POPs) belong squarely within this legacy industrial-chemical framework, and a recent CHARGE-study analysis offers an unusually direct fetal-exposure biomarker for them: PCBs, organochlorine pesticides, and PBDEs measured directly in deciduous tooth dentine layers, which record trimester-specific fetal and early-life exposure the way tree rings record annual growth. Third-trimester POPs mixture exposure was positively associated with autism (median OR 1.54), an association that reached significance in a male-only subgroup (median OR 1.75) but not in females, with no significant association for second-trimester or postnatal exposure windows, pointing to the third trimester as a candidate high-risk window for this pollutant class [82]. A separate MARBLES analysis takes a mixtures-first approach across chemical classes rather than treating POPs, phthalates, and OPEs as independent exposures: hierarchical clustering of 30 biomarkers across five chemical classes found that every resulting exposure cluster was associated with increased ASD and/or non-typical development risk, and cumulative exposure across all clusters showed the strongest association of all, reinforcing the cumulative-burden framing developed throughout this section [83]. Extending the industrial–chemical net still further, perchlorate, thiocyanate, and nitrate—all environmental anti-thyroid agents rather than classic industrial chemicals per se—were associated with altered maternal thyroid function (lower FT4 with thiocyanate; higher TT4 and lower TSH with nitrate) in the MARBLES cohort, with associations stronger among women with lower urinary iodine, offering a plausible thyroid-mediated mechanism relevant to the endocrine-disruption themes developed in Section 2.2 [84].

#### Speculative Mechanism of Developmental Action of Organic Chemicals

This category is mechanistically the most heterogeneous of the seven reviewed here, spanning legacy neurotoxicants (lead, methylmercury, PCBs), industrial byproducts (melamine and aromatic amines), lipophilic persistent pollutants (POPs), and anti-thyroid agents (perchlorate, thiocyanate, nitrate). We suspect this heterogeneity is itself instructive rather than merely a limitation of how this review is organized. We speculate that this diverse chemical class converges on neurodevelopment through at least three distinguishable, non-exclusive routes: (a) direct neurotoxicity via disruption of ion channels, mitochondrial function, or myelination, the mechanism underlying the original developmental-neurotoxicant framework [5,6]; (b) thyroid-axis disruption, most clearly demonstrated here for perchlorate, thiocyanate, and nitrate, and mechanistically continuous with the thyroid-hormone-dependent myelination pathway proposed for endocrine-disrupting chemicals in Section 2.2 [84]; and (c) cumulative mixture burden, in which no single chemical class is individually sufficient, but the aggregate xenobiotic load across POPs, phthalates, and organophosphate esters together exceeds a threshold for developmental disruption, as suggested directly by the finding that cumulative multi-class exposure carried the strongest association of any single exposure cluster [83]. The male-specific POPs association identified in the third trimester above [82] parallels the male-predominant vulnerability reported for several other pollutant classes in this review (Section 2.1 and Section 2.2), and we speculate this reflects a shared, sex-differentiated susceptibility, plausibly related to slower male fetal neurodevelopmental trajectories or sex-differential xenobiotic metabolism, rather than a mechanism specific to POPs. Given that much of this evidence derives from single cohorts (MARBLES, CHARGE) rather than the large multi-cohort consortia available for air pollution (Section 2.3), independent replication remains a priority before the mixture and thyroid-axis pathways proposed here can be considered established rather than provisional.

### 2.7. Ultra-Processed Food Intake: A Non-Pollutant Contributor to the Same Inflammatory Pathway

The six pollutant classes reviewed above are chemical or particulate exposures. Ultra-processed food (UPF) intake is not a pollutant in that sense; it is a dietary pattern, defined by the NOVA classification system as industrially formulated products typically containing additives, emulsifiers, and ingredients rarely used in home cooking. We nonetheless include it here because a growing evidence base suggests that UPF intake may drive the same downstream oxidative stress and systemic inflammation implicated throughout this review, making it a plausible additive contributor to cumulative developmental risk rather than a pollutant class in its own right. We treat it as a factor that may compound the pollutant-driven pathway this paper otherwise focuses on, not as a seventh member of that pathway. Table S7 in the Supplemental Files provides a summary list of each report described in this section, its major findings, and a link to the publication.

The strongest causal evidence comes from a small but rigorously controlled NIH inpatient trial. Twenty weight-stable adults received ad libitum ultra-processed and unprocessed diets, each for 14 days, matched for presented calories, sugar, fat, fiber, and macronutrients. Despite this matching, participants consumed roughly 500 kcal/day more on the ultra-processed diet and gained a corresponding amount of body weight, demonstrating that ultra-processing itself, independent of matched macronutrient content, can drive excess energy intake. This trial did not measure inflammatory biomarkers directly, but excess adiposity is itself a well-established driver of downstream systemic inflammation, so the trial establishes a plausible causal mechanism rather than a directly measured inflammatory endpoint [85].

Observational evidence ties UPF intake more directly to inflammatory and oxidative stress biomarkers, though these studies are cross-sectional and cannot establish causation. In 2018, the Melbourne Collaborative Cohort Study showed that every 100 g/day increase in UPF intake was associated with a 4.0% increase in high-sensitivity C-reactive protein levels in adults (hsCRP; 95% CI 2.1–5.9%), an association that held up only partly after adjusting for BMI, suggesting that UPF intake may drive inflammation through pathways beyond adiposity alone [86]. In a study of 139 Iranian adolescents, a higher UPF intake tertile was associated with significantly higher urinary 8-hydroxy-2′-deoxyguanosine (8-OHdG), a standard biomarker of oxidative DNA damage, independent of overall diet quality and lifestyle confounders [87]. Among 92 older adults with metabolic syndrome, high UPF consumers showed a broader pattern of altered oxidative and inflammatory status across plasma, neutrophil, and urinary measures, alongside poorer Mediterranean-diet adherence and lower fiber/polyphenol intake, though this cross-sectional comparison cannot separate the contribution of UPF intake itself from the broader dietary pattern it tends to displace [88].

Mechanistic reviews propose several overlapping pathways linking UPF intake to this shared inflammatory/oxidative endpoint. One review traces a cascade from emulsifier- and sweetener-driven gut barrier disruption to metabolic endotoxemia (bacterial lipopolysaccharide translocation into circulation), systemic inflammation (elevated CRP, IL-6, TNF-α), oxidative stress and mitochondrial dysfunction (ROS generation, glutathione depletion), and disrupted nutrient-sensing signaling (mTOR/AMPK/SIRT1) [89]. A related review proposes that UPF-driven mitochondrial dysfunction and oxidative stress, arising particularly from high simple-sugar intake, create a chronic pro-oxidative state that reconfigures immune cell function and lowers immune tolerance, partly via reduced NRF2 antioxidant-response activity, the same transcription factor implicated in the oxidative stress defense pathways discussed throughout this review [90]. A broader review of UPF’s role in cardiovascular disease and cancer similarly concludes that chronic UPF intake produces systemic inflammation, gut dysbiosis, endothelial dysfunction, and oxidative stress, implicating specific additives such as artificial sweeteners and sodium nitrites in genotoxicity and inflammatory microenvironments [91].

As with several pollutant classes reviewed above, the strength of evidence here varies considerably by study design. The Hall et al. trial is the only randomized controlled trial in this literature and remains the strongest causal evidence, though its primary endpoints were caloric intake and weight rather than inflammatory markers directly. Every other study cited in this section is cross-sectional/observational or a narrative/mechanistic review, which can establish association and biological plausibility but not causation. Critically, none of the studies reviewed here examined pregnancy, prenatal exposure, or neurodevelopmental/ASD outcomes directly; all evidence for a UPF–inflammation link comes from general adult or adolescent populations. Whether maternal UPF intake specifically contributes to the fetal oxidative stress/neuroinflammation pathway that this review otherwise focuses on is, at this point, an extrapolation from adjacent evidence rather than a directly tested hypothesis. We flag it here as a priority for future prenatal-cohort research rather than as an established risk factor on par with the six pollutant classes above.

#### Speculative Mechanism of Developmental Action

We speculate that maternal UPF intake could plausibly feed into the same convergent oxidative stress/neuroinflammation pathway proposed throughout this review through at least two non-exclusive routes. First, directly: emulsifier/sweetener-driven gut barrier disruption and consequent metabolic endotoxemia could produce maternal systemic inflammation (elevated CRP, IL-6, TNF-α) that plausibly crosses the placenta through the same route proposed for the maternal-stress/cortisol pathway in Section 5 below. Second, indirectly: UPF intake may displace nutrient-dense foods rich in folate, vitamin B, omega-3 fatty acids, and polyphenols, the same protective nutrients this review identifies as effect modifiers across all six pollutant classes (Section 7). Thus, high UPF intake could simultaneously increase oxidative burden and reduce the nutritional buffering capacity needed to counter it. Both routes remain speculative in the specific context of prenatal exposure and ASD risk, since, as noted above, no study to date has tested either pathway directly in a pregnant or ASD-relevant cohort.

### 2.8. Comparative Summary of Pollutant Classes

#### Reading the Comparative Table: Evidence Strength Reflects Cohort Infrastructure

Table 1 below summarizes the seven categories reviewed in this section side by side, organized by primary suspected mechanism, the developmental window in which timing appears to matter most, and the relative strength of the human versus animal evidence base. The most consistent pattern across rows is that evidence strength tracks cohort infrastructure rather than biological plausibility: air pollution and heavy metals/pesticides have the deepest human evidence not necessarily because their mechanisms are better established than, say, micro/nanoplastics, but because large multi-site cohorts (ECHO, CHARGE) happened to measure them first. This should temper any reading of the table as a ranked list of which exposures matter most. We assign the “Evidence Strength” column in Table 1 as follows: Strong denotes multiple independent human epidemiological or biomonitoring studies, typically from large multi-site cohorts, reporting associations with ASD or ASD-relevant outcomes, converging with supportive animal/mechanistic data; Moderate denotes a smaller number of human studies, or human studies with mixed or exposure-only (not ASD-outcome) findings, again alongside supportive mechanistic data; and Emerging/Weak denotes categories where human ASD-outcome data are sparse or absent and the evidence base is predominantly animal or in vitro. We apply this categorization qualitatively across the heterogeneous study designs summarized here rather than through a formal quantitative synthesis; a category’s placement reflects our judgment of the overall evidence base at the time of writing, not a systematic-review-level pooled effect estimate, and should be read alongside the individual citations in Section 2, not as a substitute for them.

## 3. Convergent Mechanisms and Speculative Developmental Correlates

Across the six pollutant classes reviewed above, a small number of mechanisms recur: oxidative stress and mitochondrial dysfunction (all classes); blood–brain and placental barrier disruption (micro/nanoplastics, tire-derived 6PPD-Q, metals); neuroinflammation and microglial dysfunction, including impaired synaptic pruning (micro/nanoplastics, air pollution); endocrine disruption, particularly thyroid hormone, and nuclear receptor (RARα/RXRα) disruption (plastic-associated EDCs, tire-derived 6PPD-Q); epigenetic modification, including DNA methylation of ASD-related genes (air pollution, and by mechanistic analogy, folate pathway disruption); and gut microbiome/gut–brain axis disturbance (micro/nanoplastics, air pollution). We speculate that these mechanisms are not independent alternative explanations for the pollutant–ASD association but rather represent multiple converging entry points into a shared final common pathway: disruption of the tightly time-constrained sequence of neurogenesis → corticogenesis → synaptogenesis → activity-dependent synaptic pruning that we have previously argued is centrally vulnerable in ASD [1]. Under this framework, the specific pollutant class, timing, and dose determine which point in this sequence is disrupted, while the downstream behavioral phenotype of impaired social communication and restricted/repetitive behavior remains convergent because it reflects failure of the same underlying developmental program regardless of the specific upstream trigger.

The glyphosate–preterm birth mechanism proposed in Section 2.5, aromatase inhibition and placental oxidative stress, maps onto the same endocrine disruption and oxidative stress themes recurring across the pollutant classes above, suggesting that even exposures whose most direct evidence is obstetric rather than neurodevelopmental may act through these same convergent pathways, with preterm birth itself serving as one upstream route into the disrupted developmental sequence.

## 4. Genetic Susceptibility Modifiers

The pollutant classes reviewed above do not act on a uniform substrate. Genetic variation, particularly in folate pathway and mitochondrial genes, modifies individual susceptibility to the oxidative stress and inflammatory mechanisms discussed throughout this review, and understanding this variation provides necessary context for why the same exposure produces ASD in one child and not another.

### 4.1. Folate Pathway Genetic Variants

Folate transport into the central nervous system is mediated primarily by folate receptor alpha (FRα) and the reduced folate carrier (RFC). Mutations in the FOLR1 gene impair FRα function directly, while polymorphisms such as the SLC19A1 variant rs1051266 reduce RFC efficiency; both routes can produce cerebral folate deficiency (CFD) independent of dietary folate intake and are associated with decreased social communication and decreased emotional recognition (note: these two routes differ substantially in both rarity and effect size, a distinction we make explicit below rather than presenting them as functionally equivalent) [2]. This genetic vulnerability compounds the folate receptor autoantibody (FRAA) mechanism detailed in our prior work [1]: approximately 70% of ASD children are FRAA-positive, compared with 5–10% of the general population, and FRAA-mediated folate transport blockade is itself believed to arise partly from environmental exposure [92], further linking the genetic and environmental threads of this review [2]. MTHFR polymorphisms, which reduce the enzymatic conversion of folate to its active 5-methyltetrahydrofolate form, represent a third, partially overlapping route to the same functional endpoint: reduced folate availability for the one-carbon metabolism pathway detailed in Section 7 below. These three routes are not equivalent in rarity or effect size, and we do not intend to imply they are. Biallelic FOLR1 loss-of-function mutations are rare and cause a severe, well-characterized monogenic CFD syndrome with early-onset neurological features; heterozygous carriers and most other FOLR1 variants have a much less certain and likely far smaller individual effect. SLC19A1 rs1051266 and the common MTHFR variants (principally C677T and A1298C) are, by contrast, frequent polymorphisms present in a large fraction of the general population, each associated with modest, population-level shifts in folate handling rather than a CFD syndrome on their own; their relevance here is as one of several small-effect susceptibility factors that may combine with pollutant exposure and FRAA status, not as equivalents to a rare pathogenic FOLR1 mutation. We flag this distinction because the genetics literature in this area is easy to over-read if rare and common variants are described together without qualification, as they were in an earlier draft of this section.

### 4.2. Gene × Pollutant Interaction: A Testable Hypothesis

Whether genetic folate pathway vulnerability amplifies pollutant-driven oxidative damage is, to our knowledge, untested directly but mechanistically plausible and worth stating as an explicit hypothesis. A child carrying FOLR1 or SLC19A1 variants has reduced baseline capacity to buffer oxidative stress via the one-carbon metabolism pathway (Section 7); the same pollutant-driven oxidative burden reviewed in Section 2.1, Section 2.2, Section 2.3, Section 2.4, Section 2.5, Section 2.6, Section 2.7 and Section 2.8 might therefore be expected to produce a larger neurodevelopmental effect in genetically vulnerable children than in children with unimpaired folate pathway function. This mirrors logic already established for synthetic folic acid and infection exposure: exposure to synthetic folic acid or infections in a child with FOLR1 or RFC polymorphisms may result in greater ASD risk due to insufficient natural folate reaching the brain or the developing fetus [2]. Testing this hypothesis for pollutant exposure specifically, rather than only for folic acid and infection, would require genotyping alongside the pollutant biomarker panels already used in cohorts such as MARBLES and ECHO, and represents, in our view, one of the more tractable near-term research priorities to emerge from this review.

### 4.3. Mitochondrial Dysfunction and the Regression Phenotype

A related but mechanistically distinct genetic vulnerability involves mitochondrial function rather than folate transport specifically. Growing evidence implicates mitochondrial dysfunction in the subset of ASD cases marked by developmental regression, in which a child shows initially strong learning, language, and memory capabilities before regressing later in childhood [2]. One study reports a difference in the observed sequence of dysfunction by regression status, with mitochondrial changes preceding immune changes in regressed children and the reverse order in non-regressed children; we present this as a single-study observation in a clinically heterogeneous population, not as a generally established developmental sequence, since regression itself is diagnosed retrospectively from parent report, the timing of biomarker sampling relative to symptom onset varies across children, and we are not aware of independent replication of this specific temporal ordering [2]. This raises the possibility that mitochondrial genetic vulnerability represents a distinct, and possibly earlier-acting, susceptibility factor than the folate pathway and inflammatory mechanisms emphasized elsewhere in this review, though the two are not mutually exclusive; oxidative stress from any of the pollutant classes reviewed above could plausibly stress mitochondrial function directly, independent of any effect on folate transport. Cord blood metabolomics offers one emerging, non-invasive window onto this pathway: machine-learning analysis of cord blood metabolic profiles was more predictive of later ASD diagnosis than maternal mid-gestational blood for both male and female offspring, and elevated cord blood acylcarnitine specifically has been shown to predict both ASD and ADHD outcomes [2]. Neither finding has yet been tested against the pollutant exposures reviewed in this paper, but both represent plausible biomarker candidates for future gene-by-environment studies.

## 5. Critical Periods and Windows of Vulnerability

A recurring finding across pollutant classes is trimester- or age-specific sensitivity: second/third-trimester sensitivity for both PM2.5 sulfate/ammonium [52] and phthalates [31]; embryonic day placental/brain crossing for 6PPD-Q during the period of major organogenesis [67]; a postnatal year-1 window for ozone [52] and for microplastic-induced synaptic pruning disruption [25]; and early-pregnancy sensitivity for the TIDES phthalate mixture [31]. As in the visual-system critical-period paradigm and our prior folate/taurine framework [1,93], we propose that ASD risk from environmental pollutants is not simply a function of cumulative lifetime dose but of the coincidence between exposure and a narrow developmental window during which the affected process (corticogenesis, thyroid-hormone-dependent myelination, or synaptic pruning) is uniquely sensitive to disruption.

A non-chemical exposure offers a useful parallel to this critical-period framework, since it is mechanistically a stress hormone rather than an industrial pollutant, but converges on the same fetal programming logic. A large Danish nationwide cohort study (over 1 million offspring) compared children prenatally exposed to systemic glucocorticoids, either for risk of preterm delivery or for maternal autoimmune/inflammatory disease, with unexposed children born to mothers with the same underlying condition, a design that reduces confounding by indication relative to a general-population comparator. Exposed offspring had a higher adjusted 15-year risk of ASD in both the preterm-delivery-risk group (6.6% vs. 4.3%; RR 1.5, 95% CI 1.2–1.9) and the autoimmune/inflammatory-disease group (4.8% vs. 3.8%; RR 1.3, 95% CI 1.1–1.5), alongside an elevated risk of ADHD and mood/anxiety/stress-related disorders; findings persisted in a sibling-matched sensitivity analysis. Mechanistically, the authors point to fetal HPA-axis programming and altered CNS structural development following excess glucocorticoid (synthetic cortisol analog) exposure during specific gestational windows, the same stress-hormone pathway invoked in critical-period frameworks of neurodevelopment generally. This supports treating maternal stress-axis dysregulation, whether from exogenous glucocorticoid treatment or, plausibly, from chronic maternal psychological stress acting through endogenous cortisol, as a further coincident-timing risk factor alongside the chemical exposures reviewed above, rather than a wholly separate causal pathway [94].

## 6. The Gut Microbiome as a Modifier of Folate/Vitamin B Availability and Neuroinflammation

A body of work independent from the pollutant literature reviewed above establishes that the gut microbiome is not a passive bystander in folate/vitamin B status but an active, bidirectional participant. We focus this section specifically on where that independent literature intersects with the pollutant-driven mechanisms reviewed in Section 2: the fecal microplastic composition finding below (citation [95]) and the shared LPS/TLR4/synaptic pruning pathway are the two most direct pollutant-microbiome links; the remaining findings establish the folate/vitamin B and neuroinflammation background needed to interpret them. Bacterial folate-synthesis genes are ubiquitous across the human gastrointestinal microbiome: in a survey of 512 gastrointestinal reference genomes spanning six major bacterial phyla, 13% contained the complete gene set for de novo folate synthesis and a further 39% could synthesize folate given dietary or cross-fed para-aminobenzoic acid (pABA), with Bifidobacterium and Lactobacillus strains identified as particularly active folate producers. Strain-level differences were substantial, with some Lactobacillus reuteri variants capable of full folate production and others genetically incapable of it. The same study reported that microbially derived folate can modulate the expression of host folate receptors, a finding with direct relevance to our own FRAα-autoantibody-mediated model of folate transport blockade in CFD: it raises the possibility that gut microbial composition, independent of autoantibody status, could itself shift folate receptor availability at the maternal–fetal or blood–brain barrier interface [96]. Table S8a–c in the Supplemental Files provide a summary list of each report described in this section, its major findings, and a link to the publication.

This relationship runs in both directions. A comprehensive 2026 review of diet, the gut-brain axis, and micronutrients reports that in humans, lower dietary consumption of vitamins B9 and B12 is associated with reduced gut microbial richness and a decline in SCFA-producing, anti-inflammatory taxa. Conversely, polyphenol-rich dietary patterns (exemplified by the Mediterranean diet) increase the relative abundance of Bifidobacterium and Lactobacillus, the same folate/B6/B12-producing genera identified above, creating the structural basis for either a virtuous or a vicious cycle depending on baseline diet quality [97]. The same review traces a mechanistic pathway directly relevant to this manuscript’s broader argument: Western diet-related dysbiosis increases intestinal permeability and bacterial lipopolysaccharide (LPS) translocation, which activates toll-like receptor 4 (TLR4) on both intestinal immune cells and microglia, driving NF-κB-mediated pro-inflammatory cytokine production, blood-brain barrier disruption, and downstream neuroinflammation with documented microglial morphological changes and altered synaptic pruning. This is the same pruning process we identify as a convergent mechanism for micro/nanoplastic exposure in Section 2.1 [25,97]. The same source notes that gut-derived short-chain fatty acids, particularly propionate, have been directly linked to ASD-relevant phenotypes: propionate administration produces social-behavior and cognitive deficits with neuroinflammation in rodent models, and autistic children show elevated fecal SCFA concentrations relative to neurotypical controls [97].

A human exploratory study adds a third, more clinically direct data point. Fecal microplastic burden in adults undergoing evaluation for metabolic dysfunction-associated steatotic liver disease (MASLD) correlated with altered gut bacterial composition, specifically increased fecal Bifidobacterium (one of the principal folate-producing genera identified above) alongside decreased Lachnospiraceae. They also showed altered hepatic immune cell populations and differential expression of 19 hepatic genes related to immune signaling and apoptosis [95]. Notably, MP burden correlated with an increase, not a decrease, in this folate-producing genus, so microplastic–microbiome interactions do not follow a single uniform direction. This is nonetheless the only study in this chapter measuring gut microbial shifts and MP burden together in living humans rather than rodents or in vitro systems, directly anchoring the pollutant-microbiome link this section is centrally concerned with.

A direct prospective test of this section’s core premise—that early gut microbial composition predicts later neurodevelopmental outcome—comes from the MARBLES cohort. Fecal samples collected between 0 and 7 months of age were analyzed via 16S rRNA sequencing and compared against gold-standard clinical classification (ASD, non-TD, or TD) around 36 months. The study found no significant differences in diversity or individual taxa between infants who later developed ASD, non-TD, or TD, though the authors describe some early compositional differences that fall short of the primary null finding. As with the MARBLES phthalate–behavior null result in Section 2.2, a well-powered, prospectively designed test in an ASD-enriched cohort did not find the hypothesized signal, which tempers, without foreclosing, the mechanistic case above; the sampling window or taxonomic resolution may simply be insufficient to detect an effect that begins earlier, in utero or via the maternal microbiome [98].

A 2026 pilot double-blind randomized controlled trial tested a multi-strain Bifidobacterium/Lactobacillus probiotic against placebo in young children (2–5 years) with confirmed ASD over four months. Overall microbial community structure did not differ significantly between groups, though fecal Bifidobacteriaceae abundance increased significantly in the probiotic group only. No significant change was detected in short-chain fatty acids or parent-reported behavioral measures, and the trial was substantially underpowered (23 of a planned 40 children completed paired sampling). We read this cautiously and, per the reviewer comment prompting this revision, treat it as explicitly hypothesis-generating rather than as support for a recommended intervention: it shows that probiotic supplementation can shift the intended bacterial family, but not that doing so changes folate status, inflammation, or behavior [99]. The authors note that this pattern, significant compositional change without corresponding behavioral change, is consistent with other small probiotic RCTs in ASD using different strains, and that individual-level variability in baseline microbiome composition appears to be a major, currently unaddressed source of heterogeneity across this trial literature. We read this cautiously: it demonstrates that Bifidobacterium-targeted probiotic supplementation is feasible and can shift the intended bacterial family in autistic children, but it does not yet demonstrate a downstream folate, inflammatory, or behavioral benefit. Larger, adequately powered trials with folate/inflammatory biomarkers as explicit secondary outcomes would be needed to test the mechanistic hypothesis proposed here.

### Speculative Developmental Correlate

We propose that the gut microbiome functions as an upstream determinant of the very folate/vitamin B sufficiency that Section 7 (below) treats as a protective effect modifier. In this way, maternal nutrient status is not solely a function of dietary intake but of microbial capacity to synthesize, cross-feed, and potentially modulate host receptor availability for these nutrients. This raises a specific, testable hypothesis beyond what either source establishes directly: if micro/nanoplastic or other pollutant exposure independently disrupts gut microbial composition (as already suggested by the ASD-associated gut microbiome shifts reported in the polyethylene-exposure mouse model in Section 2.1 [27]), this could constitute a double hit, simultaneously reducing microbial folate/vitamin B production and degrading the SCFA-mediated anti-inflammatory buffering capacity needed to counter pollutant-induced oxidative stress, at precisely the developmental window when both are most needed. Under this framework, interventions that support a folate-producing, SCFA-generating microbiome, dietary fiber/prebiotic intake, polyphenol-rich diet patterns, or targeted Bifidobacterium/Lactobacillus probiotic supplementation, as piloted above [99], remain a hypothesis to be tested, not a recommended intervention: as the probiotic pilot trial above illustrates, a compositional shift toward folate-producing taxa is achievable, but a corresponding folate, inflammatory, or behavioral benefit has not been demonstrated. Any such approach would need to be evaluated in an adequately powered trial with folate and inflammatory biomarkers as pre-specified outcomes before it could complement, let alone substitute for, direct maternal folate/Vitamin B supplementation.

## 7. Nutrient–Pollutant Interactions

We have previously proposed that cerebral folate deficiency and taurine/cysteine depletion, whether arising from folate receptor autoantibody (FRAA)-mediated transport blockade, maternal stress, and/or specific genetic alleles, disrupt the same neurogenesis and synaptic pruning windows implicated throughout this review [1]. The phthalate–micronutrient mechanistic review [34] makes this interaction explicit for plastics-associated EDCs, proposing that phthalate exposure and micronutrient status jointly disrupt prenatal one-carbon metabolism. Direct empirical support for a diet–exposure link, if not yet a diet–outcome link, comes from a 2026 ECHO Cohort analysis of nearly 1500 pregnant participants: closer adherence to US Dietary Guidelines during pregnancy was associated with lower gestational exposure to several of the priority chemical classes reviewed in this manuscript, including phthalates and certain pesticides, though not to chemicals such as PFAS that enter the body through non-dietary routes [100]. A companion ECHO analysis of gestational exposure to the same ten chemical classes across 5318 mother–child pairs found several classes independently associated with birth weight and gestational age at birth, reinforcing that this broad exposure panel, of which micro/nanoplastic-associated and industrial chemicals discussed throughout this review form a subset, carries measurable consequences for early developmental trajectories even before any neurodevelopmental endpoint is assessed [101]. Neither study measured neurodevelopmental or ASD-specific outcomes directly, so both should be read as establishing the exposure and general-outcome plausibility of the nutrient–pollutant interaction rather than testing it. Two further ECHO Cohort analyses approach the same diet–autism relationship from the outcome side rather than the exposure side and are the closest evidence in this literature to a direct test of the nutrient–pollutant framework proposed here. Across up to 6084 mother–child pairs in 14 cohorts, a higher (healthier) prenatal Healthy Eating Index score was associated with lower, i.e., more favorable, autism trait scores (SRS) in adjusted models, framing overall prenatal diet quality as a protective factor [102]. A companion ECHO analysis using a dietary mixtures approach, examining multiple nutrients jointly rather than a single composite index, similarly found prenatal dietary factor mixtures associated with child autism diagnosis and autism-related traits [103]. Neither study measured pollutant co-exposure directly, so it is together that they establish the protective, diet quality side of the interaction this section proposes, complementing the exposure-reduction evidence from Pacyga et al. above [100]; a study jointly modeling diet quality, pollutant exposure, and ASD outcome in a single cohort would be a natural next step for this literature. A closely related and more direct empirical anchor for this section’s folate-pollutant crosstalk hypothesis is discussed in Section 2.3: a 2026 MIREC cohort analysis found that an air pollution mixture (dominated by O_3_ and PM2.5) was associated with a shift toward unmetabolized folic acid and away from the bioactive 5-methyltetrahydrofolate vitamer in the third trimester [65]. This is, to date, the closest human evidence for a pollutant-driven disruption of folate handling generally, though it does not itself measure ASD outcomes and should not be read as such.

## 8. What Can Be Done: Risk Reduction and Clinical Management

### 8.1. Framing Principles

This section translates the mechanistic synthesis of Section 2, Section 3, Section 4, Section 5, Section 6 and Section 7 into actionable guidance, organized across three tiers that should not be conflated: (a) individual clinical care guided by biomarker testing, (b) household- and behavior-level exposure reduction, and (c) policy and public health action, addressed separately in Section 9. Recommendations throughout are evidence-graded using a three-tier system: **Pilot-RCT** (randomized controlled trial evidence exists, but to date only from a single small pilot-scale trial; this label describes the existence of a randomized study design and does not, by itself, indicate a certain, precise, or generalizable effect estimate, nor established clinical efficacy), **preliminary/plausible** (observational, mechanistic, or open-label evidence supports the recommendation, but controlled trial data are lacking or limited), and **hypothesis-generating/more data needed** (biologically plausible given the mechanisms reviewed in this paper, but not yet directly tested). We adopt this author-defined three-tier system, rather than a formal framework such as GRADE, because it maps directly onto the evidence types synthesized throughout this review; it is not intended to capture risk of bias, imprecision, indirectness, or applicability in the way a formal framework would, and readers seeking that level of methodological rigor should consult the primary sources cited. We label each recommendation accordingly rather than presenting all guidance as equally established. None of the tiers below, including Pilot-RCT, should be read as clinical practice guidance; they summarize the current research base and, where noted, candidate directions for individualized discussion between a patient and their treating clinician, not a standard of care. Because most of the nutrient-based interventions discussed below are food-based, low-cost, and carry minimal physiological risk even absent definitive proof of efficacy, we apply a correspondingly lower evidentiary bar to recommending them than we would to a pharmacological intervention; this asymmetry is stated explicitly rather than left implicit. This chapter is written primarily for physicians and biomedical scientists and assumes familiarity with the terminology introduced earlier in this paper; we anticipate that informed patients, family members and patient advocates may also find it informative.

### 8.2. Screening and Diagnosis

#### 8.2.1. FRAA/CFD Testing

A pilot randomized study screened 210 women planning pregnancy for folate receptor alpha autoantibodies (FRAA) and found 36 (17.1%) seropositive; 29 of these were subsequently randomized after confirming pregnancy, and children were assessed at 30 months. Among completers, the folinic acid-supplemented arm showed a substantially lower ASD diagnosis rate (1 of 10, 10%) than the standard folic acid arm (5 of the 8 completers, 62%) [104]. The study’s authors characterize this as pilot-scale and thus hypothesis-generating, given the small sample, which is not yet sufficient to change standard prenatal care or routine screening, and we adopt that same caution here. Their subsequent publication assessed the majority of these children at 4.5 years of age, finding that the ASD and non-ASD assessments were unchanged, which gives further credence to their work [104]. Given the 17.1% FRAA-positivity rate observed in this single cohort, universal preconception FRAA screening is, in principle, a research question worth pursuing, but we stop short of recommending it: evidence that folinic acid supplementation may benefit an already identified, biomarker-positive population is not equivalent to evidence that population-level screening itself improves outcomes, which would additionally require data on test sensitivity and specificity in a broader population, reproducibility, the prevalence-dependent rate of false positives and negatives and their downstream harms, and a dedicated trial showing that screening changes management and outcomes rather than merely identifying a biomarker. None of this has yet been established, and universal screening is not yet practically achievable given current testing infrastructure, which remains limited and inconsistent; faster, more scalable CFD screening approaches have been proposed but are not yet validated or in routine use [105]. We therefore do not recommend universal, or even broadly offered, preconception or early-pregnancy FRAA/CFD screening as a standard-of-care practice at this time. What we suggest instead is that FRAA/CFD testing be framed as a research-informed option for individualized discussion between an interested patient and their clinician, particularly where there is a first-degree family history of a CFD-related presentation (for example, a previous ASD child, unexplained developmental delay, or a diagnosed CFD syndrome), pending a dedicated prospective screening-outcomes trial that has not yet been conducted. We deliberately do not name specific testing laboratories or panels here, since commercial test availability changes faster than a review of this kind can track; clinicians should confirm current local and commercial testing options directly and interpret any result within this research context rather than as a validated screening test.

Evidence grade: Pilot-RCT (single small trial, not confirmatory) for the leucovorin versus folic acid outcome difference in FRAA-positive pregnancies; hypothesis-generating, extrapolated from a prevalence finding rather than from a screening-outcomes trial, for the specific screening-threshold recommendations above, which extrapolate from this trial’s prevalence finding rather than from a screening-outcomes trial directly.

#### 8.2.2. Broader Oxidative Stress Biomarker Panels

Beyond FRAA/CFD testing specifically, several biomarkers relevant to oxidative stress and the taurine/cysteine pathway (Section 3) have established assays, though their use as a routine clinical panel in this specific context remains exploratory rather than standard of care. These include plasma homocysteine (elevated levels indicate oxidative disruption of one-carbon metabolism), plasma cysteine and taurine (low levels indicate depletion of the antioxidant precursor pool), and glutathione status (the pathway’s endpoint antioxidant). We recommend measuring these only when there is clinical suspicion of elevated inflammation or oxidative stress, rather than as universal screening, both because pregnancy- and pediatric-specific reference ranges for this purpose are not well established, and because, as detailed in Section 8.3.1, supplementation decisions should be guided by evidence of depletion rather than applied prophylactically. Vitamin C, vitamin D, vitamin A, and vitamin B12 status should be tested and corrected if deficient, using standard laboratory reference ranges for pregnancy or pediatric care as applicable; we do not propose ASD-specific numeric thresholds for these, since the evidence base supports correcting deficiency generally rather than targeting a specific level for ASD risk reduction.

Evidence grade: Preliminary/plausible.

#### 8.2.3. Emerging Screening Tools

Several additional screening approaches are under active development but are not yet ready for clinical use: retinal imaging as a non-invasive proxy for cerebral folate status, machine-learning classification of neurodevelopmental risk from behavioral or imaging data, and cord blood metabolomic panels, including the acylcarnitine signal discussed in Section 4.3. We flag these as research directions rather than recommendations.

Evidence grade: Hypothesis-generating/more data needed.

### 8.3. Reducing Oxidative Stress and Supporting Nutrient Status (Path B)

#### 8.3.1. Antioxidant Nutrient Supplementation

Taurine and N-acetylcysteine (NAC) supplementation should be reserved for cases with biochemical evidence of elevated inflammation or oxidative stress, rather than applied universally, because these compounds are specifically depleted under conditions of elevated inflammatory or oxidative burden (Section 3). Supplementing in the absence of depletion has no clear rationale and is not advised. Where such evidence exists, for example, low plasma taurine, low plasma cysteine, or low glutathione, doses of roughly 1 to 3 g of taurine per day and 0.5 to 1 g of NAC per day have been used in other clinical contexts unrelated to ASD. We cite these only as descriptive reference points for a treating physician’s individualized judgment, not as a validated ASD-specific dosing recommendation, since no trial has tested this dosing against ASD-relevant outcomes in this population. Any use should be physician-supervised, and duration, if used, should track the resolution of the underlying biochemical depletion rather than follow a fixed protocol. In the absence of demonstrated depletion or elevated oxidative stress, we advise against supplementing either compound. Vitamin C, D, A, and B12 should likewise be tested and corrected only if deficient (Section 8.2.2). Omega-3 fatty acids and zinc, discussed in this review’s treatment of malnutrition as an inflammatory contributor (Section 2), follow similar logic: correcting documented insufficiency is reasonable, while supplementing in the absence of evidence of insufficiency has a weaker rationale. Whether the postnatal critical-period window described for folate intervention (most positive outcomes under age 6, with good effects through ages 11–12, and efficacy dropping sharply thereafter; Section 8.4) also applies to taurine, NAC, or antioxidant supplementation has not been directly tested. We consider it a reasonable working assumption given the shared critical-period logic developed in Section 5, but we are not aware of trial data establishing this window for Path B interventions specifically. We flag this explicitly as an evidence gap rather than assume it transfers automatically from the folate literature.

**Evidence grade:** *Preliminary/plausible for supplementation under documented evidence of depletion; hypothesis-generating for the postnatal age-window assumption.*

#### 8.3.2. Anti-Inflammatory Dietary Patterns

A Mediterranean-style dietary pattern, already discussed in our prior work as protective along the folate pathway specifically [1], plausibly extends similar benefits to the oxidative stress pathway given its established general anti-inflammatory profile and its overlap with several of the protective nutrient classes discussed above. Reducing ultra-processed food intake (Section 2.7) is a related, evidence-consistent recommendation, though, as detailed in that section, direct evidence in pregnancy or in ASD-relevant populations specifically is not yet available. A further, ASD-specific consideration deserves mention: restrictive or repetitive eating patterns are common in autistic children and can compound nutrient deficiency risk directly, creating a feedback loop in which the child’s existing feeding pattern makes correction of the very deficiencies discussed in this chapter more difficult. Clinicians working with autistic children on nutrient repletion should anticipate and plan around this rather than treating dietary recommendations as equally easy to implement across the population.

**Evidence grade:** *Preliminary/plausible.*

#### 8.3.3. Maternal Stress Reduction

The cortisol-mediated pathway linking maternal stress to reduced vitamin D and impaired folate transport, detailed in our prior work [1] and in Section 3 of this review, provides mechanistic rationale for maternal stress reduction as a component of risk-reduction guidance, independent of its well-established general benefits for maternal and fetal health. We do not propose a specific stress-reduction protocol here; established, evidence-based approaches (for example, structured mindfulness-based programs, counseling referrals where indicated, adequate sleep, and social support) are reasonable defaults and should be individualized rather than prescribed uniformly.

**Evidence grade:** *Preliminary/plausible for the stress-oxidative pathway generally; the benefit of any specific stress-reduction intervention for ASD risk specifically has not been directly tested.*

#### 8.3.4. Managing Acute Inflammatory Events (Infection, Fever)

Infection represents a third route to oxidative stress and inflammation, alongside the environmental pollutant and psychological-stress routes that are this paper’s primary focus, and we include it here specifically to flag its risk rather than to propose a validated intervention. We are not aware of trial evidence supporting a specific NAC or taurine repletion protocol following acute maternal or pediatric infection, and we do not recommend one; this is, in our assessment, a priority area for future research (Section 8.7) rather than a current clinical recommendation. What we do recommend is prompt medical attention for infection or significant fever during pregnancy or early childhood, both for the infection’s own sake and because, per the mechanisms reviewed throughout this paper, an acute inflammatory event occurring on top of an existing pollutant or chemical exposure burden may plausibly produce a larger combined oxidative stress effect than either exposure alone. In that circumstance, careful clinical monitoring is warranted, and where inflammation is significant or prolonged, laboratory evaluation for the depletion markers discussed in Section 8.2.2, followed by targeted repletion of any documented deficiency, is a logical extension of the biomarker-guided approach recommended throughout this paper. However, we note that this specific sequence—acute infection, biomarker testing, then targeted repletion—has not itself been validated in a trial.

**Evidence grade:** *Hypothesis-generating/more data needed*.

### 8.4. Addressing the Folate Path (Path A): Concise Recap

Path A is developed in full in our prior work [1] and is only briefly recapped here to avoid duplication. FRAA testing and reduced-folate, rather than folic acid, supplementation form the core of this pathway’s management, consistent with Section 8.2.1 above. In the pilot trial discussed there, FRAA-positive pregnancies supplemented with 15 mg per day of folinic acid (leucovorin) showed a markedly lower ASD diagnosis rate at 30 months and 4.5 years of follow-up than FRAA-positive pregnancies supplemented with standard folic acid [104]. Where levoleucovorin is used in place of racemic folinic acid, a dose of approximately 7.5 mg per day is the pharmacologically equivalent target, reflecting levoleucovorin’s higher bioavailable isomer content. We report these figures as the doses studied in a single pilot trial, not as an independently validated clinical dosing guideline; confirmatory trials with larger samples and longer follow-up are needed before this can be treated as standard practice, and any use should occur under a treating physician’s guidance. Dairy elimination, which lowers FRAA titers by removing the dietary trigger implicated in FRAA production, and the postnatal critical-period window (most positive outcomes under age 6, with good effects through ages 11–12, and efficacy dropping sharply thereafter), are both detailed in full in our prior work [1] and apply unchanged here.

**Evidence grade:** *Pilot-RCT (single small trial; not confirmatory) for the leucovorin dosing used in that trial, reported here as the dose studied rather than as a validated clinical recommendation; see [1] for full evidence grading of the remaining Path A components.*

### 8.5. Reducing Pollutant Exposure: Practical Guidance

#### 8.5.1. Microplastics and Plastics

General harm-reduction guidance, rather than elimination, is the appropriate framework here (Section 8.5.4). Reasonable, low-burden steps include reducing overall plastic use where practical; replacing food and beverage storage containers when visibly worn, scratched, or degraded, since physical degradation increases particle shedding (Section 2.1); avoiding heating food in plastic containers or packaging; and filtering drinking water, given the water-related exposure route discussed in Section 2.1.

**Evidence grade:** *Preliminary/plausible, extrapolated from the exposure-reduction logic of* *Section 2.1* *rather than from a trial testing these specific behaviors against a neurodevelopmental outcome.*

#### 8.5.2. Air Pollution

For pregnant individuals residing in areas with elevated ambient PM2.5 or in high-traffic corridors, use of a HEPA (high-efficiency particulate air) indoor air filter is a reasonable, low-burden household-level intervention, consistent with the indoor/outdoor air quality distinction discussed in Section 2.3 and the policy-level air quality integration proposed in Section 9.3. We are not aware of trial evidence testing HEPA filtration specifically against ASD-related outcomes; this recommendation is extrapolated from the broader PM2.5-exposure evidence reviewed in Section 2.3.

**Evidence grade:** *Preliminary/plausible*.

#### 8.5.3. Other Endocrine-Disrupting Chemicals (Pesticides, PFAS)

General reduction strategies apply similarly here: washing produce to reduce pesticide residue, water filtration (which also reduces PFAS and some metal exposure), and choosing cookware that avoids non-stick coatings associated with PFAS exposure where practical alternatives exist.

**Evidence grade:** *Preliminary/plausible, extrapolated from exposure-reduction principles rather than outcome trials.*

#### 8.5.4. Honest Limitations

We state plainly, consistent with our prior work [2], that total avoidance of the pollutant classes reviewed in this paper is not realistic given their ubiquity in food, water, air, and consumer products; none of the guidance above should be read as implying that a family who cannot fully implement it has failed to protect their child. This is harm reduction, not elimination, and the practical steps above are offered as reasonable, lower-burden actions within that framework rather than as a completeness standard.

### 8.6. Research-Informed Considerations Table (Not a Clinical Protocol)

Table 2 synthesizes Section 8.2, Section 8.3, Section 8.4 and Section 8.5 into a single reference organized by developmental stage, extending the format of our prior work’s summary-of-recommendations table [1] with an added pollutant-reduction row. We reiterate that this table is a compressed research summary, not a validated clinical protocol or standard of care; several rows summarize hypothesis-generating or single-pilot-trial evidence rather than confirmed clinical benefit. Evidence grades for each cell follow the corresponding subsection referenced in parentheses; this table is a navigational aid and should not be used without reading the underlying subsections, which contain the caveats and evidence grading omitted here for space.

### 8.7. Research Gaps and Priorities

Several priorities emerge directly from the evidence grading applied throughout this chapter. Larger and longer folinic acid trials, extending observation past the follow-up windows used to date, are needed to confirm and extend the pilot-scale RCT findings discussed in Section 8.2.1 and Section 8.4. Human biomonitoring studies directly linking microplastic or other pollutant burdens to the oxidative stress biomarkers discussed in Section 8.2.2, and ultimately to ASD outcomes directly, rather than to proxy endpoints, remain largely absent, as detailed in Section 2’s human-evidence discussion. Gene-by-pollutant interaction studies testing the hypothesis proposed in Section 4.2 have not, to our knowledge, been conducted. A standardized oxidative stress biomarker panel for clinical use, validated specifically for pregnancy and early-childhood reference ranges, does not yet exist and would materially strengthen the biomarker-guided approach this chapter recommends. Faster, more scalable CFD screening methods, referenced in Section 8.2.1, require validation before universal preconception screening becomes practically achievable. Finally, the acute-infection management pathway proposed in Section 8.3.4 is, by our own assessment, hypothesis-generating rather than established, and represents a concrete, tractable trial design: biomarker-guided repletion following acute maternal infection, tested against a monitoring-only control.

We speculate that maternal folate/Vitamin B sufficiency may function as a general effect modifier across all six pollutant classes reviewed here. They buffer oxidative stress, support the methylation capacity needed to counter pollutant-induced epigenetic disruption, and maintain the one-carbon metabolic flux required for rapid fetal neurogenesis. Thus, the same pollutant dose may produce meaningfully different neurodevelopmental outcomes depending on maternal nutrient status at the time of exposure. This hypothesis is, to our knowledge, untested directly for micro/nanoplastics or tire-derived 6PPD-Q and represents a priority direction for future work.

## 9. Public Health and Policy Implications

The individual-level clinical and behavioral guidance implied throughout this review is necessarily limited by what a single family can control. Several of the exposures reviewed in this paper—ambient air quality, plastic packaging chemistry, and industrial chemical use among them—are governed far more effectively at the population level than through individual avoidance behavior, and we close this review by considering that level explicitly.

### 9.1. The Cancer Risk-Reduction Parallel

We have drawn a parallel to cancer risk reduction throughout this review and in our prior work [1,2], and it is worth making this explicit here, with an important caveat stated up front: we invoke cancer prevention only as a structural analogy for how population-level exposure reduction can be organized (regulation, screening infrastructure, public education), not as a claim that autism is equivalent to a disease to be eliminated. Autism is a neurodevelopmental difference, not a uniformly harmful disease state, and many autistic individuals and self-advocates reasonably object to a prevention framing applied to a core neurotype rather than to a modifiable health outcome. Our focus throughout this review, and in the “risk reduction” language that follows, is narrower and more defensible: reducing exposure to substances independently linked to oxidative stress, neuroinflammation, and other adverse outcomes for pregnant people and children generally, of which a subset of the population that goes on to be diagnosed with ASD is one affected group, not the sole or primary justification. Cancer risk arises from an interplay of genetic predisposition and environmental exposure, and population-level cancer prevention has succeeded not by asking individuals to avoid all carcinogens through willpower alone, but through a combination of regulation (tobacco taxation and advertising restrictions, occupational exposure limits, food-additive bans), screening infrastructure (mammography, colonoscopy), and public education. We propose that reducing gestational and early-life exposure to the pollutant classes reviewed in this paper is amenable to a similar three-part structure: regulation of those pollutant classes, screening infrastructure for the folate pathway and oxidative stress biomarkers discussed elsewhere in this review, and public education proportionate to the actual, current evidence strength for each exposure class, as summarized in Section 2.8’s comparative table, rather than treating every exposure class as equally proven. We frame this as exposure and biomarker-risk reduction generally, not as an ASD-prevention program specifically.

### 9.2. Packaging and Plastics Regulation

Micro/nanoplastics and their associated endocrine-disrupting chemical additives (Section 2.1 and Section 2.2) are the pollutant classes in this review most directly amenable to packaging-level regulation because a meaningful share of human exposure occurs through a small number of well-characterized consumer contact points: food packaging, food storage containers, and bottled water. Plastic food containers and films tested for chemical content were found to contain thousands of distinct chemicals, many of them endocrine disruptors, including an estrogen receptor alpha activator and an androgen receptor inhibitor. Critically, packaging samples manufactured with fewer total chemicals also leached fewer toxic chemicals into food, indicating that formulation simplification is a viable, already-demonstrated regulatory lever rather than merely an aspirational one [2]. This is consistent with regulatory approaches already in place for some plastic-associated chemicals, such as bisphenol A restrictions in children’s products in some jurisdictions, and argues for extending similar formulation-disclosure or formulation-simplification requirements more broadly across food-contact plastics.

### 9.3. Air Quality Policy and Prenatal Care Integration

Ambient air pollution (Section 2.3) has the deepest human evidence base of any pollutant class reviewed in this paper and is also the class most thoroughly addressed by existing regulatory infrastructure, such as the US EPA’s National Ambient Air Quality Standards, which nonetheless permits substantial variation in exposure by neighborhood and does not currently account for the pregnancy-specific critical windows this review has emphasized, particularly the third-trimester and preconception windows. We suggest two policy-adjacent, clinically achievable steps that do not require new regulation to implement: first, integrating ambient air quality index information into routine prenatal care counseling, alongside existing guidance on nutrition and substance avoidance; and second, prioritizing indoor air quality, given this review’s gas stove and mold/water damage evidence in Section 2.3, as a household-level lever that operates independently of outdoor regulatory air quality standards and is more directly under an individual family’s control than ambient PM2.5 levels are.

### 9.4. A Cost-Benefit Framing for Low-Risk Interventions

Many of the interventions implied by this review—dietary pattern changes, nutrient sufficiency, reduced ultra-processed food intake, and household pollutant exposure reduction among them—are low-cost and carry minimal downside risk even in the absence of definitive causal proof; this asymmetry matters for public health decision-making under uncertainty. The evidence-strength gradient summarized in Section 2.8 means that not every recommendation carries equal confidence, but a food-based or behavioral recommendation with plausible but unproven benefit and negligible harm meets a substantially lower justificatory bar than a pharmacological intervention would. Set against the lifetime cost of ASD care and support, which substantially exceeds the cost of prenatal nutritional counseling or air quality-conscious prenatal care at the population level, we suggest that the cost-benefit calculus favors proactive, low-risk intervention even while the research base referenced throughout this review continues to mature.

### 9.5. Limitations

This is a narrative, author-selected review rather than a systematic review conducted to PRISMA or comparable protocol standards; we did not pre-register a review protocol, apply a priori inclusion/exclusion criteria, or independently duplicate screening and data extraction, and we discuss our search approach and its limits in Section Literature Search Strategy. Several further limitations warrant explicit statement. First, the human studies synthesized in Section 2 vary substantially in design (case-control, prospective cohort, cross-sectional), exposure assessment method (single-spot biomarker versus repeated measures, self-report versus biomonitoring), outcome definition (clinical ASD diagnosis versus continuous trait scores versus parent-reported screening instruments), and population (geography, socioeconomic status, recruitment strategy); we have not attempted a quantitative meta-analytic synthesis across these heterogeneous designs, and the qualitative evidence-strength categorization in Section 2.8 should be read with that heterogeneity in mind. Second, essentially all of the observational human evidence discussed here is subject to residual and unmeasured confounding, most importantly by shared socioeconomic, dietary, and healthcare-access factors that can independently influence both pollutant exposure and the likelihood of ASD ascertainment; few of the cohorts cited fully adjust for the same confounder set, which limits direct comparability across studies. Third, given the breadth of biomarkers, behavioral outcomes, and exposure windows examined across the underlying literature, we cannot rule out endpoint selection and multiple-comparison effects contributing to some of the individual associations reviewed, particularly for categories graded Moderate or Emerging/Weak in Table 1. Fourth, this review, like the field generally, draws disproportionately on a small number of large North American and Western European cohorts (ECHO, CHARGE, MARBLES); the generalizability of findings to other populations and exposure contexts is correspondingly limited. Fifth, we have relied in places on our own prior publications and, in a small number of instances flagged explicitly in the text, on in-press or otherwise not-yet-peer-reviewed sources; we have tried to flag the latter wherever they appear rather than presenting them with the same evidentiary weight as peer-reviewed literature, but readers should weigh those specific claims accordingly. Finally, publication bias toward positive findings is a structural limitation of the underlying evidence base that we did not attempt to formally quantify (for example, via funnel plot or trim-and-fill analysis), and it likely affects the literature summarized here to an unknown degree.

## 10. Conclusions

Fetal and early-childhood exposure to micro/nanoplastics, plastic-associated endocrine-disrupting chemicals, ambient and indoor air pollution, tire wear particles/6PPD-quinone, heavy metals, pesticides, industrial chemicals, POPs, and mixtures each show plausible, and in several cases directly tested, associations with ASD risk or ASD-relevant behavioral phenotypes. Ultra-processed food intake, while not itself a pollutant, shows a parallel signal for the same downstream inflammatory pathway and is included in this review for that reason (Section 2.7). The micro/nanoplastics literature has moved meaningfully beyond preclinical work over the course of this review: human placental, brain, and placental-explant studies now sit alongside the rodent behavioral literature, including a direct genotoxicity correlate and a fetal endocrine correlate (Section 2.1). The tire-derived 6PPD-quinone literature, though not yet tested against an ASD behavioral endpoint directly, provides some of the most mechanistically concrete evidence in this review that a ubiquitous, traffic-associated chemical reaches the fetal brain during gestation.

We situate this evidence within the two-path model of ASD-related neurodevelopmental risk introduced in our prior work (Section 1): a folate/FRAA-driven path (Path A) and an oxidative stress/inflammation-driven path (Path B). This review’s central contribution is a deepened, pollutant-focused treatment of Path B: across all seven categories reviewed in Section 2, oxidative stress, barrier disruption, neuroinflammation, endocrine disruption, and epigenetic modification recur as converging mechanisms acting on a shared developmental sequence, with trimester- and age-specific sensitivity windows analogous to the critical periods previously described for folate and taurine (Section 3 and Section 5). Genetic variation in folate pathway and mitochondrial genes further modifies this picture (Section 4), and we have proposed, as an explicit and testable hypothesis rather than an established finding, that pollutant-driven oxidative stress may secondarily compromise Path A via homocysteine and FRAA-related mechanisms (Section 4.2), supporting a unified rather than siloed mechanistic picture of ASD-related risk.

Figure 3 is a schematic representation of the genetic susceptibilities and pollutant-driven oxidative stressors on specific ASD symptom domains. It is adapted and updated from our previous schematics [1,2].

We propose, as a candidate effect modifier and research hypothesis rather than an established protective factor, that maternal nutrient status, particularly folate and vitamin B sufficiency, may modify individual vulnerability across the pollutant classes reviewed here (Section 7); this hypothesis remains directly untested for several exposure classes, as noted there. This review has attempted, in Section 8, to translate that mechanistic picture into a set of biomarker-guided, research-informed considerations—not a clinical practice guideline—organized by the same evidence-grading logic we have applied to the underlying science: Pilot-RCT, where trial evidence exists; preliminary/plausible, where mechanism and observational data align but trials are lacking; and hypothesis-generating, where we are, by our own assessment, ahead of the evidence. The interventions this translation yields—nutrient sufficiency, dietary pattern, and household-level pollutant exposure reduction among them—are consistent with the tone of our prior work: low-cost, low-risk, and reasonable to discuss with a treating clinician even before every mechanistic question in this review is fully resolved, though we reiterate that none of this constitutes an established, guideline-level clinical recommendation.

Several of the exposures reviewed here are more effectively addressed at the population level than through individual behavior alone (Section 9), a view consistent with a 2026 multidisciplinary consensus statement from exposure scientists, epidemiologists, and clinicians, which independently concluded that both toxic chemical additives to plastics and the microplastic particles themselves constitute a significant public health concern for children’s brain development and called for policy action, including bans on high-concern additive classes and expanded biomonitoring, rather than placing the exposure-reduction burden solely on individual families [106].

The research priorities identified throughout this review, including larger folinic acid trials, human biomonitoring linking pollutant burden directly to oxidative stress biomarkers and ASD outcomes, gene-by-pollutant interaction studies, and standardized biomarker panels chief among them (Section 8.7), are tractable rather than aspirational, and we hope this review’s organization by pollutant class, evidence strength, and actionable intervention tier will make them easier to pursue systematically.

## Figures and Tables

**Table 1:** Pollutant categories and suspected mechanisms of action in development.

Pollutant Class	Primary Suspected Mechanism	Critical Period	Evidence Strength (Human vs. Animal)	The Human Exposure/Biomonitoring, Human ASD-Outcome, and Experimental Mechanistic Evidence Strength
Micro-/nanoplastics (Section 2.1)	Direct particle deposition; oxidative stress; mitochondrial dysfunction; microglial priming and pruning disruption; endocrine disruption	Prenatal (placental crossing) + early postnatal	Strong, extensive animal behavioral evidence; rapidly growing human detection and mechanistic (explant) evidence; no direct human ASD-outcome study yet	Human exposure/biomonitoring: Strong (placenta, blood, breast milk). Human ASD-outcome: None to date. Experimental mechanistic: Strong (animal/in vitro)
Plastic-associated EDCs—phthalates, OPEs, bisphenols (Section 2.2)	Endocrine disruption (estrogen/androgen receptor activity); thyroid disruption; BPA-aromatase suppression; often sex-specific (male-predominant)	Prenatal, with some evidence of second-trimester sensitivity	Moderate to strong human cohort evidence (MARBLES, HOME, TIDES, ECHO); direct human + animal mechanistic data for BPA/aromatase	Human exposure/biomonitoring: Strong (urinary metabolites, large cohorts). Human ASD-outcome: Moderate (multiple cohorts assessing ASD/autistic traits directly). Experimental mechanistic: Moderate to strong (BPA-aromatase and receptor mechanisms well characterized).
Ambient and indoor air pollution (Section 2.3)	Oxidative stress and systemic inflammation; microglial activation via metal-laden PM components; possible endotoxemia (indoor sources)	2nd/3rd trimester and postnatal year 1; preconception window (3 months prior) for growth outcomes	Strong—the largest, most multi-cohort human evidence base of any category reviewed here (ECHO 28-site, CHARGE, Ontario, South Korea)	Human exposure/biomonitoring: Strong (satellite/land-use exposure models plus a growing folate-biomarker literature, Section 2.3). Human ASD-outcome: Strong (largest multi-cohort human evidence base of any category here). Experimental mechanistic: Moderate (metal-laden PM microglial-activation mechanisms less fully characterized than the epidemiology).
Tire wear particles/6PPD-quinone (Section 2.4)	Combined particulate + chemical toxicity; embedded heavy metals; 6PPD-quinone-specific toxicity	Overlaps air pollution and microplastic windows mentioned above	Weak to moderate; largely inferred from general tire-related air pollution and aquatic toxicology; direct human 6PPD–ASD data lacking	Human exposure/biomonitoring: Weak (limited direct human 6PPD-quinone biomonitoring). Human ASD-outcome: None to date. Experimental mechanistic: Weak to moderate (largely aquatic toxicology and general tire-related PM data, not 6PPD-quinone-specific developmental neurotoxicology).
Heavy metals and pesticides (Section 2.5)	Direct neurotoxicity; oxidative stress; detoxification-gene-modified susceptibility (PON1, GST)	Prenatal and early childhood	Strong human case-control and cohort evidence (CHARGE, multiple meta-analyses)	Human exposure/biomonitoring: Strong (blood/urine metal panels, widely measured). Human ASD-outcome: Strong (case-control and cohort studies with meta-analytic support). Experimental mechanistic: Strong (direct neurotoxicity and detoxification-gene-modified susceptibility well characterized).
Industrial chemicals, POPs, and mixtures (Section 2.6)	Thyroid-axis disruption; direct neurotoxicity; cumulative multi-class mixture burden	Third trimester for POPs specifically (male-specific signal observed)	Moderate; largely single-cohort human evidence (MARBLES, CHARGE); independent replication needed	Human exposure/biomonitoring: Moderate (teeth-dentine and serum POP measurement, single-cohort). Human ASD-outcome: Moderate (single-cohort associations, not yet independently replicated). Experimental mechanistic: Moderate (thyroid-axis disruption mechanisms established generally, less ASD-specific evidence).
Ultra-processed food intake (Section 2.7)	Gut barrier disruption → metabolic endotoxemia → systemic inflammation and oxidative stress; nutrient displacement	Untested in pregnancy specifically	Weakest of the seven categories: one RCT exists but for caloric intake only, not inflammation; no prenatal or ASD-specific data of any kind	Human exposure/biomonitoring: Not applicable in the pollutant-biomonitoring sense (dietary pattern, not a measured chemical body-burden). Human ASD-outcome: None; untested in pregnancy specifically. Experimental mechanistic: Weak (extrapolated from general adult/non-pregnant inflammation literature, not directly tested in a prenatal or ASD-relevant model).

**Table 2:** Research-informed considerations on candidate risk-reduction and risk-management approaches by evidence tier (not a clinical protocol).

	Preconception	Pregnancy	Early Childhood (0–3 yrs)
Screening	Not a recommended universal screen; FRAA testing may be discussed as a research-informed option, particularly with first-degree family history of a CFD-related presentation (Section 8.2.1)	FRAA/CFD testing remains a research-informed, patient-clinician discussion, not a routine screen, if not previously done; monitor for infection/fever	FRAA/CFD testing if symptoms emerge and not previously tested; consider broader oxidative stress biomarker panel if clinical suspicion (Section 8.2.2)
Nutrient Support	Test and correct vitamin C/D/A/B12 if deficient; if FRAA-positive, leucovorin 15 mg/day (or levoleucovorin 7.5 mg/day) in place of standard folic acid is a pilot-trial-studied, physician-supervised option, not an established standard (Section 8.4)	If already on leucovorin/levoleucovorin for FRAA-positivity, continuation is a physician-supervised decision, not an automatic default; test/correct vitamin C/D/A/B12; taurine (roughly 1–3 g/day) and NAC (roughly 0.5–1 g/day) are descriptive reference doses from other clinical contexts, not validated ASD-specific dosing, and apply only with biomarker evidence of oxidative stress/depletion, under physician guidance (Section 8.3.1)	If already on leucovorin/levoleucovorin for FRAA-positivity, physician-supervised continuation may be considered; the pilot-trial critical-period data suggest the largest effect under age 6, with benefit through roughly age 11–12 (Section 8.4), but this window has not been independently confirmed; taurine/NAC only with documented depletion; correct vitamin deficiencies
Pollutant Reduction *(these steps are supported by evidence that they reduce measured exposure; direct evidence that they improve neurodevelopmental outcomes specifically is not yet available for most items below)*	Begin harm-reduction steps: reduce plastic use, filter water, HEPA filtration if high-traffic/high-PM2.5 area (Section 8.5)	Continue harm-reduction steps; prenatal counseling on ambient air quality index alongside standard nutrition/substance counseling (Section 8.5.2 and Section 9.3)	Continue household-level harm reduction; HEPA filtration; minimize plastic food-contact exposure for infant/toddler feeding
Referral Triggers	FRAA-positive result; first-degree family history of ASD or CFD-related diagnosis	Acute infection or significant fever (→ prompt care; consider biomarker testing per 8.3.4); maternal asthma/diabetes/obesity (Section 2.3); new FRAA-positive result	Developmental delay or regression (Section 4.1); documented nutrient depletion; relevant family history

## Data Availability

No new data were created or analyzed in this study. Data sharing is not applicable to this article.

## Supplementary Materials

The following supporting information can be downloaded at https://www.mdpi.com/article/10.3390/cimb48090878/s1.

## Abbreviations

The following abbreviations are used in this manuscript:

10HDA	10-hydroxy-2-decenoic acid
6PPD	N-(1,3-dimethylbutyl)-N’-phenyl-p-phenylenediamine
8-OHdG	8-hydroxy-2′-deoxyguanosine
ADHD	attention-deficit/hyperactivity disorder
ADOS(-2)	Autism Diagnostic Observation Schedule
AMPK	AMP-activated protein kinase
aOR	adjusted odds ratio
ASD	autism spectrum disorder
AUC	area under the curve
BBB	blood–brain barrier
BBOEP	bis(2-butoxyethyl) phosphate
BCPP	bis(1-chloro-2-propyl) phosphate
BDCPP	bis(1,3-dichloro-2-propyl) phosphate
BIS	Barwon Infant Study
BMI	body mass index
BPA	bisphenol A
BPF	bisphenol F
BPS	bisphenol S
CBS	cystathionine beta-synthase
CDO	cysteine dioxygenase
CFD	cerebral folate deficiency
CFU	colony-forming units
CHAMACOS	Center for the Health Assessment of Mothers and Children of Salinas (cohort)
CHARGE	Childhood Autism Risks from Genetics and the Environment (study)
CI	confidence interval
CNS	central nervous system
CO	carbon monoxide
COVID-19	Coronavirus Disease 2019
CRP	C-reactive protein
CSAD	cysteine sulfinic acid decarboxylase
CYP19A1	cytochrome P450 family 19 subfamily A member 1 (aromatase gene)
DBUP	di-n-butyl phosphate
DDT	dichlorodiphenyltrichloroethane
DIBP	diisobutyl phosphate
DINCH	1,2-cyclohexane dicarboxylic acid diisononyl ester (di-isononyl cyclohexane-1,2-dicarboxylate)
DNA	deoxyribonucleic acid
DPHP	diphenyl phosphate
DSM-5	Diagnostic and Statistical Manual of Mental Disorders, 5th Edition
EARLI	Early Autism Risk Longitudinal Investigation (cohort)
ECHO	Environmental influences on Child Health Outcomes (cohort/consortium)
EDC(s)	endocrine-disrupting chemical(s)
EDEN	Etude des Determinants pre et postnatals du developpement et de la sante de l’ENfant (French mother–child cohort)
EMA	Early Markers for Autism (study)
FDR	false discovery rate
FOLR1	folate receptor alpha gene
FRα	folate receptor alpha
FRAA	folate receptor autoantibody
FT4	free thyroxine
GABA	gamma-aminobutyric acid
GSH	glutathione
GST	glutathione S-transferase
GSTM1	glutathione S-transferase Mu 1
GSTP1	glutathione S-transferase Pi 1
HEPA	high-efficiency particulate air
HOME	Health Outcomes and Measures of the Environment (cohort)
HPA	hypothalamic–pituitary–adrenal (axis)
hsCRP	high-sensitivity C-reactive protein
IL-6/IL-8/IL-17a	interleukin-6/interleukin-8/interleukin-17a
iPSC	induced pluripotent stem cell
IQ	intelligence quotient
LPS	lipopolysaccharide
MARBLES	Markers of Autism Risk in Babies—Learning Early Signs (study)
MASLD	metabolic dysfunction-associated steatotic liver disease
MBP	monobutyl phthalate
MBzP	monobenzyl phthalate
MCNP	monocarboxyisononyl phthalate
MCPP	mono(3-carboxypropyl) phthalate
MHiBP	monohydroxy-isobutyl phthalate
MHxP	monohexyl phthalate
MiBP	monoisobutyl phthalate
MNP(s)	micro/nanoplastic(s)
MP(s)	microplastic(s)
MR	magnetic resonance (spectroscopy)
MRI	magnetic resonance imaging
MTHFR	methylenetetrahydrofolate reductase
mTOR	mechanistic target of rapamycin
NAC	N-acetyl cysteine
NO_2_	nitrogen dioxide
NOVA	food classification system by degree of processing (Monteiro et al. [107]; not an acronym)
NOx	oxides of nitrogen
NP(s)	nanoplastic(s)
NRF2	nuclear factor erythroid 2-related factor 2
O_3_	ozone
OPE(s)	organophosphate ester(s)
OR	odds ratio
pABA	para-aminobenzoic acid
PBDE(s)	polybrominated diphenyl ether(s)
PCB(s)	polychlorinated biphenyl(s)
PE	polyethylene
PET	positron emission tomography
PFAS	per- and polyfluoroalkyl substances
PM	particulate matter
PM0.1	ultrafine particulate matter (aerodynamic diameter < 0.1 µm)
PM10	particulate matter (aerodynamic diameter < 10 µm)
PM2.5	fine particulate matter (aerodynamic diameter < 2.5 µm)
PON1	paraoxonase 1
POPs	persistent organic pollutants
PRISMA	Preferred Reporting Items for Systematic Reviews and Meta-Analyses
PS	polystyrene
RARα/RXRα	retinoic acid receptor alpha/retinoid X receptor alpha
RCT(s)	randomized controlled trial(s)
RFC	reduced folate carrier
ROS	reactive oxygen species
RR	risk ratio (relative risk)
rRNA	ribosomal ribonucleic acid
SAH	S-adenosylhomocysteine
SAM	S-adenosylmethionine
SCFA	short-chain fatty acid
SDQ	Strengths and Difficulties Questionnaire
SLC19A1	solute carrier family 19, member 1
SIRT1	sirtuin 1
SO_2_	sulfur dioxide
SRS(-2)	Social Responsiveness Scale (2nd Edition)
TBR1	T-box brain transcription factor 1
TD	typical development/typically developing
TDCPP	tris(1,3-dichloro-2-propyl) phosphate
THF	tetrahydrofolate
TIDES	The Infant Development and the Environment Study
TLR4	toll-like receptor 4
TNF-α	tumor necrosis factor alpha
TSH	thyroid-stimulating hormone
TT4	total thyroxine
TWP(s)	tire wear particle(s)
UPF	ultra-processed food
US	United States
VI	layer VI (cortical layer six)
WUI	wildland–urban interface
