# Supplementary material for: Pollutant Burden in Autism Spectrum Disorder: Mechanistic Convergence, Genetic Susceptibility, and Clinical Translation
Source: Curr Issues Mol Biol. 2026 Aug 29;48(9):878. doi: 10.3390/cimb48090878 (PMC13605344; doi:10.3390/cimb48090878)
Supplement: Supplementary file 1 [file cimb-48-00878-s001.zip › cimb-4503202-supplementary.pdf]

Supplementary Data – ASD Pollutant Literature by Pollutant/Factor Group

Table S1 – Micro/Nanoplastics (Direct Particle)

**Effect / Outcome Category**	**Major Findings**	**Year**	**Ref**
Background / review / framework	General neurotoxicity reviews describe blood--brain barrier (BBB) penetration, oxidative stress, mitochondrial dysfunction, protein aggregation, and chronic neuroinflammation as consistent findings across in vitro and in vivo MNP studies.	2025	[1]
Background / review / framework	General neurotoxicity reviews describe blood--brain barrier (BBB) penetration, oxidative stress, mitochondrial dysfunction, protein aggregation, and chronic neuroinflammation as consistent findings across in vitro and in vivo MNP studies.	2025	[2]
Background / review / framework	Gestational and lactational nanoplastic exposure in rats selectively bioaccumulates in the fetal cerebellum and damages myelin development [6], while a separate rat model demonstrates that maternal PS-NP exposure induces P53-mediated ferritinophagy and ferroptosis in the offspring hippocampus, producing measurable learning and memory decline.	2024	[3]
ASD / autism-related outcome	A 2025 systematic review focused specifically on micro/nanoplastic-induced endocrine disruption in rodents makes the ASD connection explicit at the review level, synthesizing the mechanistic case for endocrine-mediated MNP neurodevelopmental risk across the rodent literature.	2025	[4]
Background / review / framework	Maternal PS-NP exposure via drinking water also produces a sex-specific developmental milestone shift (earlier eye-opening in males) and detectable ex vivo MRI brain-structure differences in cortex, hippocampus, striatum, and corpus callosum.	2023	[5]
Neurodevelopmental / behavioral outcome	More specific to fetal neurodevelopment, polystyrene nanoplastics (PS-NPs) are directly neurotoxic to primary neuronal and glial cultures, inducing apoptosis and oxidative stress [3], and in human iPSC-derived cortical spheroids, a three-dimensional model of the fetal cortex, short-term PS-microplastic exposure paradoxically increases proliferation/progenitor gene expression while longer exposure.	2022	[6]
Neurodevelopmental / behavioral outcome	Maternally ingested PS-NPs reach the offspring brain via breast milk, disrupting neural stem cell function and producing sex-specific (female-predominant) cognitive deficits.	2022	[7]
Neurodevelopmental / behavioral outcome	More specific to fetal neurodevelopment, polystyrene nanoplastics (PS-NPs) are directly neurotoxic to primary neuronal and glial cultures, inducing apoptosis and oxidative stress [3], and in human iPSC-derived cortical spheroids, a three-dimensional model of the fetal cortex, short-term PS-microplastic exposure paradoxically increases proliferation/progenitor gene expression while longer exposure.	2020	[8]
Neurodevelopment: narrative review	Synthesizes evidence that plastics-derived chemicals and microplastic/nanoplastic particles may harm the developing brain; calls for regulatory action. Directly frames the microplastics-neurodevelopment link.	2026	[9]

Effect / Outcome Category	Major Findings	Year	Ref
ASD / autism-related outcome	Human evidence directly linking plastics exposure to ASD severity, as opposed to preclinical phenotypes or diagnosis risk alone, remains rare; the CHARGE study addressed this gap by scoring parental occupational chemical exposures during the periconceptional and prenatal periods against validated ASD severity and adaptive-skill measures (ADOS-2, Vineland) in 532 parent--child pairs, finding that.	2025	[10]
Background / review / framework	Fecal microplastic burden in adults undergoing evaluation for metabolic dysfunction-associated steatotic liver disease (MASLD) correlated with altered gut bacterial composition --- specifically increased fecal Bifidobacterium (one of the principal folate-producing genera identified above) alongside decreased Lachnospiraceae --- as well as altered hepatic immune cell populations and differential.	2024	[11]
Neurodevelopmental / behavioral outcome	Maternal polystyrene-microplastic exposure impairs social behavior in offspring mice.	2023	[12]
Background / review / framework	Gestational and lactational nanoplastic exposure in rats selectively bioaccumulates in the fetal cerebellum and damages myelin development [6], while a separate rat model demonstrates that maternal PS-NP exposure induces P53-mediated ferritinophagy and ferroptosis in the offspring hippocampus, producing measurable learning and memory decline.	2024	[13]
Neurodevelopmental / behavioral outcome	A companion mouse study from the same laboratory extends the microglial-pruning finding above from social behavior specifically to a broader ADHD-like phenotype: lifelong polystyrene-nanoplastic exposure, beginning in utero, produced hyperactivity, increased risk-taking, and impaired motor learning and executive function in adult offspring, together with a lowered epileptic threshold and.	2025	[14]
ASD / autism-related outcome	Most strikingly, a mouse model using polyethylene (PE) microplastics, the most common of consumer plastics, explicitly tested and confirmed the microplastic--ASD hypothesis: prenatal PE exposure produced impaired social interaction and repetitive behavior, altered brain glucose metabolism and gene expression, and ASD-associated gut microbiome shifts, assessed via PET imaging, MR spectroscopy, and.	2022	[15]
Neurodevelopmental / behavioral outcome	Neonatal polystyrene-nanoplastic exposure impairs microglia-mediated synaptic pruning during early neurodevelopment, via disrupted microglial autophagy and energy metabolism, and producing lasting social behavioral defects in adulthood [9], directly implicating the same pruning process we have previously linked to oxidative stress and taurine sufficiency.	2024	[16]

Table S2a
Plastic-Associated EDCs — Bisphenols

Effect / Outcome Category	Major Findings	Year	Ref
ASD / autism-related outcome	In the EDEN mother--child cohort, prenatal BPA was positively associated with the SDQ relationship-problems subscale at age 3 and with hyperactivity-inattention at age 5 in boys, though this outcome measure captures general behavioral difficulty rather than an ASD diagnosis specifically.	2017	[17]
ASD / autism-related outcome	Higher prenatal maternal BPA associated with greater ASD symptom scores at age 2 and clinician-confirmed ASD diagnosis at age 9, but only in boys with a genetic profile conferring low brain aromatase enzyme activity (matched OR 6.24 for diagnosis, 95% CI 1.02-38.26; population-attributable fraction 12.6% in this subgroup). BPA predicted CYP19A1 (aromatase) brain-promoter hypermethylation in cord blood, replicated in an independent cohort (CCCEH-MN). Mouse studies: mid-gestation BPA suppresses brain aromatase, reduces amygdala neuron number/activity, and produces male-specific ASD-like social and repetitive-behavior deficits, mirroring aromatase-knockout mice; postnatal 10HDA (an estrogenic compound) reversed the behavioral and neuronal deficits. Combines a genetically-stratified human ASD-diagnosis outcome with a full mechanistic pathway and a candidate reversibility intervention.	2024	[18]

Table S2b
Plastic-Associated EDCs — General

Effect / Outcome Category	Major Findings	Year	Ref
ASD / autism-related outcome	A systematic review and meta-analysis of seven studies of phthalates and social behavior/ASD found the aggregate evidence to be slight and indeterminate, cautioning that exposure misclassification and mixture effects likely obscure true associations.	2020	[19]

Table S2c
Plastic-Associated EDCs — Melamine/Aromatic Amines

Effect / Outcome Category	Major Findings	Year	Ref
Gestational duration & fetal growth — not autism	Examines melamine (used in plastic dinnerware/food-contact materials) and aromatic amine exposure in relation to birth outcomes; not an autism outcome.	2025	[20]

Table S2d
Plastic-Associated EDCs — Organophosphate Esters (OPE)

Effect / Outcome Category	Major Findings	Year	Ref
ASD diagnosis & autism traits (SRS) — original EHP DOI	Duplicate/earlier-DOI registration of the same study as row above (journal transitioned publishers) — retain only one citation in final manuscript.	2025	[21]

Effect / Outcome Category	Major Findings	Year	Ref
ASD relative risk (null)	NO clear evidence that gestational OPE exposure was associated with ASD risk (null finding); possible effect modification by homeownership.	2024	[22]
Child cognition (IQ) — neurodevelopmental outcome, not autism diagnosis specifically	[PARTIAL SOURCE — abstract/highlights/intro only, no full methods/results/discussion available] Diphenyl phosphate (DPHP, detected in 96.4% of samples) was inversely associated with cognition scores (β per doubling = -0.46, 95% CI -0.90, -0.02). Bis(butoxyethyl) phosphate (BBOEP), bis(1-chloro-2-propyl) phosphate (BCPP), and bis(2-methylphenyl) phosphate (BMPP) above the detection limit were associated with higher cognition scores mainly in boys (sex interaction significant except for BMPP). Authors conclude DPHP exposure was associated with modestly lower cognitive functioning; given widespread exposure, population impact could be meaningful despite small effect size.	2026	[23]
Early-childhood behavioral outcomes	Examines OPE exposure in relation to early-childhood behavioral outcomes (broader than ASD diagnosis alone). [DOI CORRECTED: source table listed .109647, which belongs to a different paper (see Phthalates row / ms ref 56). Corrected to .109649 per PubMed.]	2025	[24]
Maternal immune/inflammatory markers during pregnancy — not an autism outcome directly (mechanistic pathway paper)	[PARTIAL SOURCE — abstract/highlights/intro only, no full methods/results/discussion available] Prenatal BDCPP (a TDCPP metabolite) associated with elevated maternal IL-8 in the pooled sample (β=0.06 per doubling, 95% CI 0.02-0.11) and in both contributing sites. Among participants of race/ethnicity other than non-Hispanic White, diphenyl phosphate (DPHP) and bis(2-chloroethyl) phosphate (BCETP) showed stronger associations with higher TNF-α. Sex-dimorphic associations observed for IL-6, direction varying by OPE metabolite. Other OPE-immune marker associations not consistent across sites. Authors frame this as a possible intrauterine-environment pathway warranting further study of downstream child health effects (not a direct autism finding).	2026	[25]
Childhood obesity — not autism	Mixed associations between gestational OPE exposure and childhood obesity risk; no consistent direction across age groups.	2024	[26]

Table S2e
Plastic-Associated EDCs — Phthalates

Effect / Outcome Category	Major Findings	Year	Ref
ASD / autism-related outcome	Mixture-modeling approaches developed for this literature, such as those applied to prenatal phthalate, paraben, and phenol co-exposure in the CHAMACOS cohort, illustrate the broader methodological point that these compounds rarely occur in isolation; that particular study examined childhood adiposity	2021	[27]

Effect / Outcome Category	Major Findings	Year	Ref
	rather than a neurodevelopmental outcome, but the same weighted-quantile-sum mixture framework.		
Background / review / framework	In the TIDES pregnancy cohort, a one-quintile increase in an early-pregnancy phthalate metabolite mixture was associated with a higher Social Responsiveness Scale (SRS-2) total score and worse adaptive skills, with a stronger effect in boys.	2021	[28]
Preschool behavioral problems (autism-enriched cohort)	Overall phthalate-behavior associations not significant, BUT mid-pregnancy phthalate biomarker concentrations specifically associated with MORE behavior problems; no association for phthalate mixture as a whole.	2025	[29]
ASD / non-TD classification	Each chemical cluster (via hierarchical clustering + LDA) associated with increased ASD and/or non-TD risk; cumulative exposure across all clusters showed increased risk.	2025	[30]
ASD / autism-related outcome	A large cohort-only meta-analysis discussed further in Section 2.5 independently corroborates two specific phthalate metabolites, mono-3-carboxypropyl phthalate and monobutyl phthalate, as significantly associated with ASD across the pooled cohort literature.	2024	[31]
Internalizing/externalizing behavior (CBCL, ages 1.5-5y)	Several phthalates (not the alternative plasticizer DINCH) associated with a small-to-modest INCREASE in externalizing behavior; sex modified some associations; no robust internalizing-behavior associations.	2025	[32]
Neurodevelopmental / behavioral outcome	A 2024 mechanistic review proposes that phthalate exposure and micronutrient status, including folate and B-vitamin status, jointly disrupt prenatal one-carbon and lipid metabolism as a shared pathway toward suboptimal neurodevelopment, directly bridging the endocrine-disruption and nutrient-metabolism literatures.	2024	[33]
ASD / non-typical development / cognitive development	Rare POSTNATAL (infancy, not just prenatal) exposure window study — 32 EDC biomarkers quantified in infant urine and examined against later ASD/Non-TD/cognitive outcomes.	2024	[34]
ASD / autism-related outcome	A combined analysis of the HOME and EARLI cohorts using quantile g-computation found smaller, cohort-specific associations between a six-metabolite phthalate mixture and SRS scores, with the high-familial-ASD-risk EARLI cohort showing a different direction of effect than the general-population HOME cohort, thus suggesting that population susceptibility modifies the phthalate--ASD relationship.	2024	[35]
Background / review / framework	A prospective cohort measuring seven phthalate metabolites and BPA found trimester-specific associations with motor, problem-solving, and social developmental scores at 18 months, with free-T4 (thyroid hormone) partially mediating the association between low-molecular-weight phthalates and communication scores.	2025	[36]

Table S3a
Ambient Air Pollution (PM2.5, NO2, O3)

Effect / Outcome Category	Major Findings	Year	Ref
ASD / autism-related outcome	A separate 2024 meta-analysis of 14 studies found PM2.5 significantly associated with ASD, with a weaker, non-significant signal for NO2.	2024	[37]
Neurodevelopmental / behavioral outcome	As we have argued in a commentary on the South Korean cohort, these pollutant-level associations are best conceptualized not as single sufficient causes but as risk modifiers that interact with genetic variants, maternal immune and metabolic status, and micronutrient sufficiency, particularly one-carbon metabolism, to shift the probability of a neurodevelopmental outcome rather than determine it.	2026	[38]
ASD / autism-related outcome	A broader review of prenatal PM exposure across the neuropsychiatric spectrum, ASD, ADHD, anxiety, depression, and schizophrenia, situates the ASD-specific findings above within this wider set of outcomes and mechanisms, reinforcing that PM2.5/PM10 neurotoxicity is not ASD-selective but rather reflects a shared vulnerability of the developing brain to oxidative and inflammatory insult.	2025	[39]
ASD / autism-related outcome	A 2.18-million-birth Ontario, Canada cohort found that sulfate and ammonium, specific PM2.5 components, not PM2.5 mass overall, were independently associated with ASD, with the second and third trimesters (gestational weeks 21--36) representing the sensitive exposure window; postnatal ozone exposure during the first year of life was independently associated as well.	2025	[40]
ASD / autism-related outcome	A mechanistic network-toxicology analysis modeling long-term PM2.5 exposure against ASD symptom severity, rather than diagnosis risk, independently supports a neuroinflammatory pathway, implicating microglial activation and impaired synaptic plasticity as the proximate route from chronic particulate exposure to symptom exacerbation.	2025	[41]
ASD / autism-related outcome	An earlier narrative review identified prenatal PM2.5 as the most consistently replicated air-pollution signal for ASD across the literature to that point.	2016	[42]
ASD diagnosis / autism traits (SRS)	Higher prenatal PM2.5, NO2, and 8-hr max ozone associated with increased autism-related traits (SRS, quantile regression) and higher ASD diagnosis risk; effects present even at low pollution levels.	2025	[43]
ASD diagnosis	First study of PM0.1 chemical components and ASD; first-year iron/copper/sodium/manganese in PM0.1 linked to higher ASD odds; pregnancy PM0.1-2.5 nitrate/sulfate associated with increased ASD risk.	2026	[44]
ASD / autism-related outcome	A 2025 meta-analysis of 27 studies (n = 369,460, with 47,973 ASD cases) found gestational PM2.5 exposure associated with a 15% increase in overall ASD risk, with the largest effect in the third trimester, and postnatal year-2 exposure carrying the highest risk of any window examined.	2025	[45]
ASD / autism-related outcome	A companion multi-site cohort study, using data from 8,370 mother--child pairs across 28 ECHO Cohort sites, examined general behavioral outcomes (Child Behavior Checklist internalizing/externalizing scores) rather than ASD diagnosis directly, but adds valuable trimester-	2026	[46]

Effect / Outcome Category	Major Findings	Year	Ref
	specific granularity: first-trimester PM2.5 was associated with externalizing behavior, second-trimester PM2.5 with.		
Gestational duration & fetal growth — not autism	Examines indoor combustion (gas stove NO2) and damp/mold housing exposure against birth outcomes; not an autism outcome but relevant indoor-exposure context.	2026	[47]
Neurodevelopmental / behavioral outcome	A 1.44-million-birth South Korean nationwide cohort (linking National Health Insurance Service birth records to insurance-claims diagnoses, with follow-up to 13 years) found that each 0.01 ppm increase in prenatal NO2 was associated with an 18% higher risk of long-term neurodevelopmental disorders (aOR 1.18, 95% CI 1.15--1.20), and each 0.001 ppm increase in SO2 with a 1% increase in risk (aOR.	2026	[48]
Neurodevelopmental / behavioral outcome	A methodologically distinct New York City cohort (470 mother--child pairs) used a distributed-lag model to identify week-by-week windows of susceptibility rather than trimester-level exposure, finding that first-trimester NO2 (approximately gestational weeks 29--30) and second-trimester PM2.5 (weeks 17--18) were each independently, inversely associated with cognitive scores at ages 1 and 3, with.	2024	[49]

Table S3b
Ambient Air Pollution — Wildfire-Specific PM2.5

Effect / Outcome Category	Major Findings	Year	Ref
ASD diagnosis	Intense prenatal wildfire smoke exposure associated with increased likelihood of ASD diagnosis.	2026	[50]
Serum PAHs & oxylipins (immune biomarker) — not autism outcome directly	Gestational wildfire PM2.5 exposure associated with higher serum benzo[k]fluoranthene (a PAH) and altered lipoxygenase (LOX)-derived oxylipins (lower free-pool, higher esterified-pool levels) measured 7-13 months post-exposure, suggesting long-lasting effects on immune/inflammatory lipid signaling; no significant associations with PBDEs, PCBs, or other PAHs. Findings did not survive FDR correction (preliminary, underpowered). Not an autism outcome, but a mechanistic/biomarker study of gestational wildfire PM2.5 relevant to the wildfire-ASD evidence base (complements the Sherris et al. and O'Sharkey et al. wildfire rows above).	2025	[51]
Preterm birth (not autism-specific)	Wildfire-specific PM2.5 exposure associated with increased risk of preterm birth; complements wildfire-ASD findings above.	2025	[52]

Table S4
Tire Wear Particles / 6PPD-Quinone

Effect / Outcome Category	Major Findings	Year	Ref
ASD / autism-related outcome	Across these classes, we identify convergent final-common-pathway mechanisms --- oxidative stress, blood--brain and placental barrier disruption, neuroinflammation and microglial dysfunction, epigenetic	2024	[53]

Effect / Outcome Category	Major Findings	Year	Ref
	modification, and gut--brain axis disturbance --- that plausibly intersect with the critical periods of fetal neurogenesis, synaptogenesis, and postnatal synaptic pruning previously described in.		
Mechanistic / pathway evidence	Cross-species convergence strengthens the case further: pulsed embryonic 6PPD-Q exposure in coho salmon produced dose-dependent mortality (as low as 21% survival to hatching at the highest tested concentration) with transcriptional disruption of vascular permeability pathways [36], indicating that 6PPD-Q\'s developmental toxicity is not confined to a single taxon or organ system.	2023	[54]
Background / review / framework	In a human trophoblast--endometrial coculture model using BeWo spheroids and Ishikawa cells as surrogates for the blastocyst and endometrial epithelium, both 6PPD and 6PPD-Q reduced trophoblast attachment and invasion and downregulated endometrial receptivity markers --- the first direct evidence that these compounds may impair embryo implantation itself, upstream of any subsequent fetal exposure.	2025	[55]
Mechanistic / pathway evidence	A further placental-penetration-focused review documents MP-induced cytotoxicity and genotoxicity in placental cells and altered gestation time, offspring weight, and litter number in mouse models [40], while the p53-mediated ferroptosis mechanism already described for generic PS-nanoplastics in Section 2.1 [7] provides one plausible molecular route by which tire-derived nanoplastic fragments.	2025	[56]
Background / review / framework	A companion scoping review on child health confirms MP detection in placenta, amniotic fluid, umbilical cord, fetal membranes, and umbilical vein blood, with continued postnatal exposure via breast milk.	2025	[57]
Background / review / framework	Chronic 6PPD-Q exposure in male mice reduced testosterone, impaired sperm quality and in vitro fertilization outcomes, and produced transcriptomic/metabolomic changes enriched in spermatogenesis, apoptosis, arginine biosynthesis, and sphingolipid metabolism in testicular tissue.	2024	[58]
Neurodevelopmental / behavioral outcome	A 2025 comprehensive review concludes that MPs can cross the placental barrier, impair placental function, and alter fetal growth, and specifically documents reduced offspring dopamine, serotonin, acetylcholine, GABA, and oxytocin accompanied by anxiety-like behavior, social deficits, and cognitive impairment.	2026	[59]
Background / review / framework	Tires are manufactured from a complex matrix that generally includes styrene-butadiene rubber, natural rubber, carbon black, silica, and sulfur/zinc-based vulcanization agents; a 2023 comprehensive review of tire wear particle formation, measurement, and physicochemical properties confirms that TWPs commonly carry embedded heavy metals and mineral residue contributed by road-surface abrasion.	2023	[60]
Mechanistic / pathway evidence	Beyond the particle itself, tires are formulated with the antioxidant 6PPD (N-(1,3-dimethylbutyl)-N′-phenyl-p-phenylenediamine), which reacts with atmospheric ozone to form 6PPD-quinone (6PPD-Q) --- a transformation product responsible for acute mass die-offs in coho salmon and increasingly implicated in mammalian developmental toxicity.	2023	[61]

Table S5
Heavy Metals & Pesticides

Effect / Outcome Category	Major Findings	Year	Ref
ASD & developmental delay diagnosis	Professional indoor pesticide application associated with 2-FOLD increased odds of ASD; more frequent exposure associated with higher odds of ASD for most application types.	2025	[62]
Cognitive & adaptive behavior (MSEL, VABS) in ASD/DD/TD	Flea/tick insecticide exposure (indoor, outdoor, and on pets) associated with lower cognitive and adaptive-behavior developmental-quotient scores.	2025	[63]
ASD / autism-related outcome	The developmental neurotoxicant paradigm itself is not new: Grandjean and Landrigan\'s landmark reviews identified lead, methylmercury, polychlorinated biphenyls, arsenic, and toluene as proven human developmental neurotoxicants in 2006 [28], later expanding the list to include manganese, fluoride, chlorpyrifos, DDT, tetrachloroethylene, and polybrominated diphenyl ethers in 2014 --- explicitly.	2014	[64]
ASD / autism-related outcome	The developmental neurotoxicant paradigm itself is not new: Grandjean and Landrigan\'s landmark reviews identified lead, methylmercury, polychlorinated biphenyls, arsenic, and toluene as proven human developmental neurotoxicants in 2006 [28], later expanding the list to include manganese, fluoride, chlorpyrifos, DDT, tetrachloroethylene, and polybrominated diphenyl ethers in 2014 --- explicitly.	2006	[65]
ASD / autism-related outcome	A comprehensive narrative review of environmental chemical exposures, including metals, pesticides, air pollutants, and solvents, across the growing epidemiological literature found consistent, if individually modest, associations with ASD risk.	2014	[66]
ASD / autism-related outcome	Much of this biomarker literature rests on infrastructure built by population-based case-control studies such as the Early Markers for Autism (EMA) study, which archived prenatal and neonatal biospecimens from California births specifically to enable this kind of retrospective toxicant-biomarker analysis; its cohort profile documents the biologic-plausibility rationale that underlies the.	2021	[67]
ASD diagnostic classification (biomarker/rule-out test)	Machine-learning model using hair elemental biodynamics (including toxic metals Pb, Mn, As) as an ASD rule-out diagnostic; AUC 0.79, 97% negative predictive value, 96% sensitivity.	2025	[68]
ASD / autism-related outcome	A systematic review found that 92% of 37 reviewed studies detected an association between estimated environmental toxicant exposure (metals, pesticides, air pollutants, PCBs) and ASD risk, and additionally reviewed toxicant-biomarker and gene--environment interaction studies implicating detoxification-gene polymorphisms (PON1, GSTM1/GSTP1) as individual susceptibility modifiers.	2014	[69]
ASD / autism-related outcome	Mercury has the most targeted recent evidence within this class: a 2023 PRISMA-2020 systematic review and meta-analysis of mercury body-burden biomarkers across hair, whole blood, plasma, red blood cells, and urine found that ASD children show a reduced capacity to eliminate mercury relative to neurotypical children, supporting mercury as a candidate environmental contributor alongside the.	2023	[70]

Table S6a
Perchlorate, Thiocyanate, Nitrate (Thyroid-Disrupting Agents)

Effect / Outcome Category	Major Findings	Year	Ref
Maternal thyroid function (mechanistic pathway)	Higher thiocyanate associated with lower FT4; higher nitrate associated with higher TT4/lower TSH; associations stronger when urinary iodine <150 ug/L.	2026	[71]

Table S6b
Persistent Organic Pollutants (POPs)

Effect / Outcome Category	Major Findings	Year	Ref
ASD diagnosis	[PARTIAL SOURCE — abstract/highlights/intro only, no full methods/results/discussion available] Quantified 7 PCBs, 2 OCPs, and 4 BDEs in deciduous teeth dentine layers. Third-trimester POPs mixture positively associated with autism (median OR=1.54, 95% CI 0.99-2.83); in sex-stratified analysis this association was significant in males (median OR=1.75, 95% CI 1.13-3.2) but not females (OR=1.16, 95% CI 0.33-3.5). No significant associations for 2nd-trimester or postnatal POPs mixtures. Authors conclude third trimester is a potential high-risk window for POPs exposure and autism risk, particularly in males.	2026	[72]

Table S6c
Chemical Mixtures (Multi-Class)

Effect / Outcome Category	Major Findings	Year	Ref
Autism diagnosis & autism-related traits	Examines dietary-nutrient mixtures (not pollutants) as predictors of autism diagnosis/traits — useful contrast/protective-factor framing.	2025	[73]
Birth outcomes — not autism	Companion paper to row above; examines the same 10-chemical-class exposure panel against birth weight/gestational age outcomes.	2026	[74]
Prenatal exposure characterization — not outcome	Better adherence to dietary guidelines during pregnancy associated with lower exposure to some chemical classes (e.g., some phenols/parabens) but not others (e.g., PFAS/metals sometimes higher via healthy foods like fish).	2026	[75]

Table S7
Published Research: Ultra-Processed Foods, Inflammation, and Oxidative Stress

Effect / Outcome Category	Major Findings	Year	DOI
Caloric intake & weight gain — not	First RCT on UPFs (Hall et al., NIH). Inpatient, calorie-matched, ad libitum feeding trial found ultra-processed diets caused ~500 kcal/day higher intake and significant weight gain vs. unprocessed diets. Did not directly	2019	[76]

Effect / Outcome Category	Major Findings	Year	DOI
inflammation directly	measure inflammatory biomarkers, but established a causal mechanism (excess energy intake/adiposity) driving downstream inflammation.		
Inflammatory biomarker (hsCRP)	In 2,018 adults, every 100 g/day increase in UPF intake was associated with a 4.0% increase in hsCRP (95% CI: 2.1–5.9%), partly independent of BMI.	2022	[77]
Oxidative DNA damage biomarker (8-OHdG)	In 139 Iranian adolescents, higher UPF intake tertile was linked to significantly higher urinary 8-OHdG (a biomarker of oxidative DNA damage), independent of diet quality and lifestyle confounders.	2021	[78]
Oxidative stress & inflammatory biomarkers	In 92 older adults with metabolic syndrome, high UPF consumers (vs. low) showed poorer Mediterranean diet adherence, lower fiber/polyphenol intake, and altered oxidative/inflammatory status across plasma, neutrophils, and urine.	2023	[79]
Background / review / framework	Narrative review concluding UPFs drive dyslipidemia, hypertension, and obesity, and that chronic intake produces systemic inflammation, gut dysbiosis, endothelial dysfunction, and oxidative stress; additives (artificial sweeteners, sodium nitrites) implicated in genotoxicity and inflammatory microenvironments linked to cancer.	2025	[80]
Mechanistic / pathway evidence	Review proposing UPF-driven mitochondrial dysfunction and oxidative stress (from high simple-sugar diets) create a chronic pro-oxidative state that reconfigures immune cell function, lowers immune tolerance, and raises infection risk (partly via reduced NRF2 activity).	2021	[81]
Mechanistic / pathway evidence	Mechanistic review tracing a cascade from emulsifier/sweetener-driven gut barrier disruption to metabolic endotoxemia (LPS translocation), systemic inflammation (↑CRP, IL-6, TNF-α), oxidative stress and mitochondrial dysfunction (ROS, glutathione depletion), and disrupted nutrient-sensing (mTOR/AMPK/SIRT1) and insulin resistance.	2025	[82]

Note on evidence strength: Most UPF entries above are cross-sectional/observational studies or narrative/mechanistic reviews, which show association and plausible biological pathways rather than proven causation. The Hall et al. (2019) study is the sole randomized controlled trial and remains the strongest causal evidence in the field, though its primary endpoints were caloric intake and weight rather than inflammatory markers directly.

Table S8a
Nutrient-Pollutant Interactions / Diet

Effect / Outcome Category	Major Findings	Year	Ref
ASD / autism-related outcome	This view is consistent with broader syntheses estimating that environmental factors may account for as much as half of ASD risk variance across parental, prenatal, perinatal, and chemical-exposure domains [51], and with our own prior work situating genetic susceptibility, maternal immune activation, autoimmune disease, and folate-related metabolic vulnerability within a shared inflammatory and.	2025	[83]

Effect / Outcome Category	Major Findings	Year	Ref
ASD / autism-related outcome	This view is consistent with broader syntheses estimating that environmental factors may account for as much as half of ASD risk variance across parental, prenatal, perinatal, and chemical-exposure domains [51], and with our own prior work situating genetic susceptibility, maternal immune activation, autoimmune disease, and folate-related metabolic vulnerability within a shared inflammatory and.	2025	[84]
ASD / autism-related outcome	As in the visual-system critical-period paradigm and our prior folate/taurine framework [29,30], we propose that ASD risk from environmental pollutants is not simply a function of cumulative lifetime dose but of the coincidence between exposure and a narrow developmental window during which the affected process (corticogenesis, thyroid-hormone-dependent myelination, or synaptic pruning) is.	2011	[85]
Autism diagnosis & SRS traits	Higher (healthier) prenatal Healthy Eating Index score associated with LOWER (better) SRS autism-trait scores in adjusted models — diet as a protective factor.	2024	[86]
ASD / autism-related outcome	This view is consistent with broader syntheses estimating that environmental factors may account for as much as half of ASD risk variance across parental, prenatal, perinatal, and chemical-exposure domains [51], and with our own prior work situating genetic susceptibility, maternal immune activation, autoimmune disease, and folate-related metabolic vulnerability within a shared inflammatory and.	2024	[87]

Table S8b
Gut Microbiome (Pathway)

Effect / Outcome Category	Major Findings	Year	Ref
ASD diagnosis (biological pathway)	Mixed/inconclusive evidence that early infant gut microbiome composition predicts later ASD diagnosis — potential mediating pathway for chemical-exposure effects (e.g., microplastic-gut-brain axis).	2026	[88]
ASD / autism-related outcome	No significant change was detected in Lactobacillaceae, faecal short-chain fatty acids, or parent-reported behavioral measures, though the trial was substantially underpowered (23 of a planned 40 children completed paired sampling, with COVID-19-related recruitment disruption cited as a major contributor).	2026	[89]
Background / review / framework	The same study reported that microbially derived folate can modulate expression of host folate receptors, a finding with direct relevance to our own FRAα-autoantibody-mediated model of folate transport blockade in CFD: it raises the possibility that gut microbial composition, independent of autoantibody status, could itself shift folate receptor availability at the maternal-fetal or blood-brain.	2019	[90]
Background / review / framework	A comprehensive 2026 review of diet, the gut-brain axis, and micronutrients reports that in humans, lower dietary consumption of vitamins B9 and B12 is associated with reduced gut microbial richness and evenness and a specific decrease in Odoribacter, a short-chain-fatty-acid (SCFA)-producing genus	2026	[91]

Effect / Outcome Category	Major Findings	Year	Ref
	implicated in both anti-inflammatory signaling and epigenetic regulation; conversely,.		

Table S8c
Maternal Stress / Glucocorticoid Exposure (Non-Chemical)

Effect / Outcome Category	Major Findings	Year	Ref
ASD / autism-related outcome	Prenatal glucocorticoid exposure (for preterm-delivery risk or maternal autoimmune/inflammatory disease) vs unexposed offspring of mothers with the SAME underlying disease. Adjusted 15-year ASD risk higher in both exposed groups (6.6% vs 4.3%, RR 1.5, 95% CI 1.2-1.9; and 4.8% vs 3.8%, RR 1.3, 95% CI 1.1-1.5); also higher ADHD and mood/anxiety/stress-disorder risk. Findings held in a sibling-matched sensitivity analysis. NOT a chemical pollutant — an exogenous stress-hormone/HPA-axis exposure, included as a non-chemical parallel to the critical-period/fetal-programming framework (Section 2.8).	2025	[92]

References

1. Araújo, A.M.; Mota, C.; Ramos, H.; Faria, M.A.; Carvalho, M.; Ferreira, I.M.P.L.V.O. The Neurotoxic Threat of Micro- and Nanoplastics: Evidence from In Vitro and In Vivo Models. *Arch Toxicol* **2025**, *99*, 3505–3525, doi:10.1007/s00204-025-04091-3.
2. Baroni, A.; Moulton, C.; Cristina, M.; Sansone, L.; Belli, M.; Tasciotti, E. Nano- and Microplastics in the Brain: An Emerging Threat to Neural Health. *Nanomaterials (Basel)* **2025**, *15*, 1361, doi:10.3390/nano15171361.
3. Chen, J.; Yan, L.; Zhang, Y.; Liu, X.; Wei, Y.; Zhao, Y.; Li, K.; Shi, Y.; Liu, H.; Lai, W.; et al. Maternal Exposure to Nanopolystyrene Induces Neurotoxicity in Offspring through P53-Mediated Ferritinophagy and Ferroptosis in the Rat Hippocampus. *J Nanobiotechnology* **2024**, *22*, 651, doi:10.1186/s12951-024-02911-9.
4. Fowler, L.F.; Burry, T.N.; Maekawa, A.S.; Cahill, L.S. A Systematic Review and Experimental Study of Micro/Nanoplastic-Induced Endocrine Disruption in Rodents: Potential Links to Autism Spectrum Disorder. *Horm Behav* **2025**, *175*, 105818, doi:10.1016/j.yhbeh.2025.105818.
5. Harvey, N.E.; Mercer, G.V.; Stapleton, D.; Steeves, K.L.; Hanrahan, J.; Cui, M.; Aghaei, Z.; Spring, S.; Helm, P.A.; Simpson, A.J.; et al. Maternal Exposure to Polystyrene Nanoplastics Impacts Developmental Milestones and Brain Structure in Mouse Offspring. *Environmental Science: Advances* **2023**, *2*, 622–628, doi:10.1039/d2va00227b.
6. Hua, T.; Kiran, S.; Li, Y.; Sang, Q.-X.A. Microplastics Exposure Affects Neural Development of Human Pluripotent Stem Cell-Derived Cortical Spheroids. *J Hazard Mater* **2022**, *435*, 128884, doi:10.1016/j.jhazmat.2022.128884.
7. Jeong, B.; Baek, J.Y.; Koo, J.; Park, S.; Ryu, Y.-K.; Kim, K.-S.; Zhang, S.; Chung, C.; Dogan, R.; Choi, H.-S.; et al. Maternal Exposure to Polystyrene Nanoplastics Causes Brain Abnormalities in Progeny. *J Hazard Mater* **2022**, *426*, 127815, doi:10.1016/j.jhazmat.2021.127815.
8. Jung, B.-K.; Han, S.-W.; Park, S.-H.; Bae, J.-S.; Choi, J.; Ryu, K.-Y. Neurotoxic Potential of Polystyrene Nanoplastics in Primary Cells Originating from Mouse Brain. *Neurotoxicology* **2020**, *81*, 189–196, doi:10.1016/j.neuro.2020.10.008.

9. Marsit, C.J.; Bennett, D.H.; Birnbaum, L.S.; Brody, C.; Eskenazi, B.; Gartner, E.; Hertz-Picciotto, I.; Hirtz, D.; Miller, P.; Perera, F.; et al. Protecting the Developing Brains of Children from Plastics-Derived Chemicals and Microplastic Particles. *Environ Int* **2026**, *212*, 110284, doi:10.1016/j.envint.2026.110284.
10. McCanlies, E.C.; Gu, J.K.; Ma, C.C.; Sanderson, W.T.; Ludeña-Rodriguez, Y.J.; Hertz-Picciotto, I. The Effects of Parental Occupational Exposures on Autism Spectrum Disorder Severity and Skills in Cognitive and Adaptive Domains in Children with Autism Spectrum Disorder. *Int J Hyg Environ Health* **2025**, *268*, 114613, doi:10.1016/j.ijheh.2025.114613.
11. Schwenger, K.J.P.; Ghorbani, Y.; Bharatselvam, S.; Chen, L.; Chomiak, K.M.; Tyler, A.C.; Eddingsaas, N.C.; Fischer, S.E.; Jackson, T.D.; Okrainec, A.; et al. Links between Fecal Microplastics and Parameters Related to Metabolic Dysfunction-Associated Steatotic Liver Disease (MASLD) in Humans: An Exploratory Study. *Sci Total Environ* **2024**, *953*, 176153, doi:10.1016/j.scitotenv.2024.176153.
12. So, Y.H.; Shin, H.S.; Lee, S.H.; Moon, H.J.; Jang, H.J.; Lee, E.-H.; Jung, E.-M. Maternal Exposure to Polystyrene Microplastics Impairs Social Behavior in Mouse Offspring with a Potential Neurotoxicity. *Neurotoxicology* **2023**, *99*, 206–216, doi:10.1016/j.neuro.2023.10.013.
13. Tian, L.; Zhang, Y.; Chen, J.; Liu, X.; Nie, H.; Li, K.; Liu, H.; Lai, W.; Shi, Y.; Xi, Z.; et al. Effects of Nanoplastic Exposure during Pregnancy and Lactation on Neurodevelopment of Rat Offspring. *Journal of Hazardous Materials* **2024**, *474*, 134800, doi:10.1016/j.jhazmat.2024.134800.
14. Vignon, A.N.; Dudon, G.; Oliva, G.; Thirard, S.; Alenda, U.G.; Brugoux, A.; Cazevieille, C.; Imbert, J.; Bellières, C.; Lehmann, S.; et al. Lifelong Exposure to Polystyrene-Nanoplastics Induces an Attention-Deficit Hyperactivity Disorder-like Phenotype and Impairs Brain Aging in Mice. *J Hazard Mater* **2025**, *494*, 138640, doi:10.1016/j.jhazmat.2025.138640.
15. Zaheer, J.; Kim, H.; Ko, I.O.; Jo, E.-K.; Choi, E.-J.; Lee, H.-J.; Shim, I.; Woo, H.-J.; Choi, J.; Kim, G.-H.; et al. Pre/Post-Natal Exposure to Microplastic as a Potential Risk Factor for Autism Spectrum Disorder. *Environ Int* **2022**, *161*, 107121, doi:10.1016/j.envint.2022.107121.
16. Zou, L.; Xu, X.; Wang, Y.; Lin, F.; Zhang, C.; Liu, R.; Hou, X.; Wang, J.; Jiang, X.; Zhang, Q.; et al. Neonatal Exposure to Polystyrene Nanoplastics Impairs Microglia-Mediated Synaptic Pruning and Causes Social Behavioral Defects in Adulthood. *Environ Sci Technol* **2024**, *58*, 11945–11957, doi:10.1021/acs.est.4c03231.
17. Philippat, C.; Nakiwala, D.; Calafat, A.M.; Botton, J.; De Agostini, M.; Heude, B.; Slama, R.; EDEN Mother–Child Study Group Prenatal Exposure to Nonpersistent Endocrine Disruptors and Behavior in Boys at 3 and 5 Years. *Environ Health Perspect* **2017**, *125*, 097014, doi:10.1289/EHP1314.
18. Symeonides, C.; Vacy, K.; Thomson, S.; Tanner, S.; Chua, H.K.; Dixit, S.; Mansell, T.; O'Hely, M.; Novakovic, B.; Herbstman, J.B.; et al. Male Autism Spectrum Disorder Is Linked to Brain Aromatase Disruption by Prenatal BPA in Multimodal Investigations and 10HDA Ameliorates the Related Mouse Phenotype. *Nat Commun* **2024**, *15*, 6367, doi:10.1038/s41467-024-48897-8.
19. Radke, E.G.; Braun, J.M.; Nachman, R.M.; Cooper, G.S. Phthalate Exposure and Neurodevelopment: A Systematic Review and Meta-Analysis of Human Epidemiological Evidence. *Environ Int* **2020**, *137*, 105408, doi:10.1016/j.envint.2019.105408.
20. Choi, G.; Xun, X.; Bennett, D.H.; Meeker, J.D.; Morello-Frosch, R.; Sathyanarayana, S.; Schantz, S.L.; Trasande, L.; Watkins, D.; Pellizzari, E.D.; et al. Associations of Prenatal Urinary Melamine, Melamine Analogues, and Aromatic Amines with Gestational Duration and Fetal Growth in the ECHO Cohort. *Environ Int* **2025**, *195*, 109227, doi:10.1016/j.envint.2024.109227.
21. Ames, J.L.; Ferrara, A.; Feng, J.; Alexeeff, S.; Avalos, L.A.; Barrett, E.S.; Bastain, T.M.; Bennett, D.H.; Buckley, J.P.; Carignan, C.C.; et al. Exposure to Organophosphate Ester Flame Retardants and Plasticizers during Pregnancy and Autism-Related Outcomes in the ECHO Cohort. *Environ Health Perspect* **2025**, doi:10.1289/EHP16177.

22. Choi, J.W.; Oh, J.; Bennett, D.H.; Kannan, K.; Tancredi, D.J.; Miller, M.; Schmidt, R.J.; Shin, H.-M. Gestational Exposure to Organophosphate Esters and Autism Spectrum Disorder and Other Non-Typical Development in a Cohort with Elevated Familial Likelihood. *Environ Res* **2024**, *263*, 120141, doi:10.1016/j.envres.2024.120141.
23. Ghassabian, A.; Etzel, T.; Ames, J.L.; O'Connor, T.G.; Buckley, J.P.; Shahin, S.; Herbstman, J.B.; Barrett, E.S.; Liang, D.; Croen, L.A.; et al. Prenatal Exposure to Organophosphate Ester Flame Retardants and Child Cognition: Findings from the Environmental Influences on Child Health Outcomes Cohort. *Environ Pollut* **2026**, *390*, 127446, doi:10.1016/j.envpol.2025.127446.
24. Oh, J.; Quirós-Alcalá, L.; Li, X.; Kannan, K.; Lyu, W.; Avalos, L.A.; Barrett, E.S.; Bastain, T.M.; Buckley, J.P.; Cintora, P.; et al. Prenatal Exposure to Organophosphate Ester Flame Retardants and Behavioral Outcomes in Early Childhood in the Environmental Influences on Child Health Outcomes (ECHO) Cohort. *Environ Int* **2025**, *202*, 109649, doi:10.1016/j.envint.2025.109649.
25. Oh, J.; Van de Water, J.; O'Connor, T.G.; Scheible, K.; Starling, A.P.; Dabelea, D.M.; Fichorova, R.N.; Schmidt, R.J.; Hertz-Picciotto, I.; Bennett, D.H. Prenatal Exposure to Organophosphate Ester Flame Retardants and Plasticizers and Maternal Immune Responses in Three ECHO Cohorts. *Environ Res* **2026**, *305*, 124940, doi:10.1016/j.envres.2026.124940.
26. Peterson, A.K.; Alexeeff, S.E.; Ames, J.L.; Feng, J.; Yoshida, C.; Avalos, L.A.; Barrett, E.S.; Bastain, T.M.; Bennett, D.H.; Buckley, J.P.; et al. Gestational Exposure to Organophosphate Ester Flame Retardants and Risk of Childhood Obesity in the Environmental Influences on Child Health Outcomes Consortium. *Environ Int* **2024**, *193*, 109071, doi:10.1016/j.envint.2024.109071.
27. Berger, K.; Hyland, C.; Ames, J.L.; Mora, A.M.; Huen, K.; Eskenazi, B.; Holland, N.; Harley, K.G. Prenatal Exposure to Mixtures of Phthalates, Parabens, and Other Phenols and Obesity in Five-Year-Olds in the CHAMACOS Cohort. *Int J Environ Res Public Health* **2021**, *18*, 1796, doi:10.3390/ijerph18041796.
28. Day, D.B.; Collett, B.R.; Barrett, E.S.; Bush, N.R.; Swan, S.H.; Nguyen, R.H.N.; Szpiro, A.A.; Sathyanarayana, S. Phthalate Mixtures in Pregnancy, Autistic Traits, and Adverse Childhood Behavioral Outcomes. *Environ Int* **2021**, *147*, 106330, doi:10.1016/j.envint.2020.106330.
29. Choi, J.W.; Bennett, D.H.; Calafat, A.M.; Tancredi, D.J.; Miller, M.; Schmidt, R.J.; Shin, H.-M. Gestational Phthalate Exposure and Behavioral Problems in Preschool-Aged Children with Increased Likelihood of Autism Spectrum Disorder. *Int J Hyg Environ Health* **2025**, *263*, 114483, doi:10.1016/j.ijheh.2024.114483.
30. Choi, J.W.; Jang, H.; Kuiper, J.R.; Bennett, D.H.; Schmidt, R.J.; Shin, H.-M. Gestational Exposures to Mixtures of Multiple Chemical Classes and Autism Spectrum Disorder in the MARBLES Study. *Environ Res* **2025**, *278*, 121646, doi:10.1016/j.envres.2025.121646.
31. Duque-Cartagena, T.; Dalla, M.D.B.; Mundstock, E.; Neto, F.K.; Espinoza, S.A.R.; de Moura, S.K.; Zanirati, G.; Padoin, A.V.; Jimenez, J.G.P.; Stein, A.T.; et al. Environmental Pollutants as Risk Factors for Autism Spectrum Disorders: A Systematic Review and Meta-Analysis of Cohort Studies. *BMC Public Health* **2024**, *24*, 2388, doi:10.1186/s12889-024-19742-w.
32. Oh, J.; Buckley, J.P.; Upadhyaya, S.; Kannan, K.; Barrett, E.S.; Bastain, T.M.; Breton, C.V.; Eick, S.M.; Geiger, S.D.; Ghassabian, A.; et al. Prenatal Exposure to Phthalates and Alternative Plasticizers and Emotional and Behavioral Outcomes in Early Childhood in the Environmental Influences on Child Health Outcomes (ECHO) Cohort. *Environ Int* **2025**, *202*, 109647, doi:10.1016/j.envint.2025.109647.
33. Parenti, M.; Slupsky, C.M. Disrupted Prenatal Metabolism May Explain the Etiology of Suboptimal Neurodevelopment: A Focus on Phthalates and Micronutrients and Their Relationship to Autism Spectrum Disorder. *Adv Nutr* **2024**, *15*, 100279, doi:10.1016/j.advnut.2024.100279.
34. Sotelo-Orozco, J.; Calafat, A.M.; Cook Botelho, J.; Schmidt, R.J.; Hertz-Picciotto, I.; Bennett, D.H. Exposure to Endocrine Disrupting Chemicals Including Phthalates, Phenols, and Parabens in Infancy: Associations with Neurodevelopmental Outcomes in the MARBLES Study. *Int J Hyg Environ Health* **2024**, *261*, 114425, doi:10.1016/j.ijheh.2024.114425.

35. Yu, E.X.; Braun, J.M.; Lyall, K.; Hertz-Picciotto, I.; Fallin, M.D.; Croen, L.A.; Chen, A.; Xu, Y.; Yolton, K.; Newschaffer, C.J.; et al. A Mixture of Urinary Phthalate Metabolite Concentrations During Pregnancy and Offspring Social Responsiveness Scale Scores. *Epidemiology* **2024**, *35*, 84–93, doi:10.1097/EDE.0000000000001682.
36. Al-Saleh, I.; Aljerayed, Y.; Gheith, M.; Alobaid, N.; Alenazi, H.; Elkhatib, R.; Aldhalaan, H.; Alnemer, M.; Mohamed, G.; Shoukri, M. Longitudinal Analysis of Maternal Exposure to Phthalates and Bisphenol A and Their Impact on Infant Neurodevelopment and Autistic Behavior: The Potential Mediating Role of Thyroid Hormones. *Int J Hyg Environ Health* **2025**, *269*, 114647, doi:10.1016/j.ijheh.2025.114647.
37. Amnuaylojaroen, T.; Parasin, N.; Saokaew, S. Exploring the Association between Early-Life Air Pollution Exposure and Autism Spectrum Disorders in Children: A Systematic Review and Meta-Analysis. *Reprod Toxicol* **2024**, *125*, 108582, doi:10.1016/j.reprotox.2024.108582.
38. Ayoub, G. Prenatal Exposure to Ambient Air Pollution and Risk of Neurodevelopmental Disorders in Offspring: A Nationwide Population-Based Cohort Study - A Commentary on Oh and Song 2026. *J Child Psychol Psychiatry* **2026**, doi:10.1111/jcpp.70167.
39. Bae, H.J.; Mony, T.J.; Park, S.J. Association between Prenatal Particulate Matter Exposure and Neuropsychiatric Disorders Development. *Biomol Ther (Seoul)* **2025**, *33*, 557–571, doi:10.4062/biomolther.2025.031.
40. Cloutier, M.; Yu, C.; Talarico, R.; Hawken, S.; Chen, H.; Weichenthal, S.; Cakmak, S.; Hebbern, C.; Gunz, A.; van Donkelaar, A.; et al. Prenatal Exposure to Fine Particulate Matter Components and Autism Risk in Childhood. *JAMA Netw Open* **2025**, *8*, e2538882, doi:10.1001/jamanetworkopen.2025.38882.
41. Dou, J.; Zhang, K.; Xie, R.; Xu, H.; Pan, Q.; Xiao, X.; Luo, Y.; Xu, S.; Xiao, W.; Wu, D.; et al. Investigating the Effects of Long-Term Fine Particulate Matter Exposure on Autism Spectrum Disorder Severity: Evidence from Multiple Analytical Approaches. *Toxics* **2025**, *13*, 922, doi:10.3390/toxics13110922.
42. Flores-Pajot, M.-C.; Ofner, M.; Do, M.T.; Lavigne, E.; Villeneuve, P.J. Childhood Autism Spectrum Disorders and Exposure to Nitrogen Dioxide, and Particulate Matter Air Pollution: A Review and Meta-Analysis. *Environ Res* **2016**, *151*, 763–776, doi:10.1016/j.envres.2016.07.030.
43. Ghassabian, A.; Dickerson, A.S.; Wang, Y.; Braun, J.M.; Bennett, D.H.; Croen, L.A.; LeWinn, K.Z.; Burris, H.H.; Habre, R.; Lyall, K.; et al. Prenatal Air Pollution Exposure and Autism Spectrum Disorder in the ECHO Consortium. *Environ Health Perspect* **2026**, *134*, 324–334, doi:10.1021/EHP.6c00106.
44. Goodrich, A.J.; Bennett, D.H.; Kleeman, M.J.; Tancredi, D.J.; Ludeña, Y.J.; Hertz-Picciotto, I.; Schmidt, R.J. Fine and Ultrafine Particulate Matter Components and Autism Spectrum Disorder (ASD). *Environ Int* **2026**, *208*, 110068, doi:10.1016/j.envint.2026.110068.
45. Mamun, M.A.; Al-Mamun, F.; Roy, N.; Raquib, A.; Kaggwa, M.M.; ALmerab, M.M.; Gozal, D.; Hossain, M.S. Preconception and Gestational versus Postnatal Exposure to Air Pollutants and Risk of Autism Spectrum Disorder: A Systematic Review and Meta-Analysis. *Int Arch Occup Environ Health* **2025**, *98*, 33–57, doi:10.1007/s00420-024-02112-9.
46. Oh, J.; Ghassabian, A.; Aschner, J.; Calub, C.A.; Chiu, Y.-H.M.; Croen, L.A.; Dickerson, A.S.; Goodrich, A.J.; Herrera, T.; Hertz-Picciotto, I.; et al. Prenatal Exposure to Nitrogen Dioxide, Fine Particulates, and Ozone in Relation to Child Behavior: The Environmental Influences on Child Health Outcomes Cohort. *Environ. Sci. Technol.* **2026**, *60*, 14341–14354, doi:10.1021/acs.est.6c00031.
47. Oh, J.; Quirós-Alcalá, L.; Burjak, M.; Ross, A.J.; Croen, L.A.; Dickerson, A.S.; Ferrara, A.; Habre, R.; Hertz-Picciotto, I.; Karagas, M.R.; et al. Prenatal Exposure to Gas Stoves, Mold, and Water Damage: Associations with Gestational Duration and Fetal Growth in the ECHO Cohort. *Environmental Pollution* **2026**, *399*, 128219, doi:10.1016/j.envpol.2026.128219.

48. Oh, T.K.; Song, I.-A. Prenatal Exposure to Ambient Air Pollution and Risk of Neurodevelopmental Disorders in Offspring: A Nationwide Population-Based Cohort Study. *Journal of Child Psychology and Psychiatry n/a*, doi:10.1111/jcpp.70120.
49. Perera, F.; Miao, Y.; Ross, Z.; Rauh, V.; Margolis, A.; Hoepner, L.; Riley, K.W.; Herbstman, J.; Wang, S. Prenatal Exposure to Air Pollution during the Early and Middle Stages of Pregnancy Is Associated with Adverse Neurodevelopmental Outcomes at Ages 1 to 3 Years. *Environ Health* **2024**, *23*, 95, doi:10.1186/s12940-024-01132-9.
50. O'Sharkey, K.; Mitra, S.; Chow, T.; Goodrich, A.J.; Conlon, K.C.; Nuño, M.; Bennett, D.H.; Raffuse, S.; Astaneh, M.; Zhao, Y.; et al. Prenatal Exposure to Wildfire-Related PM2.5 and Autism Spectrum Disorder in Children Born in California between 2001-2019. *Environ Int* **2026**, *208*, 110131, doi:10.1016/j.envint.2026.110131.
51. Park, E.; Shen, Q.; Zhang, Z.; O'Brien, C.E.; Goodrich, A.J.; Angel, E.E.; Hertz-Picciotto, I.; Tancredi, D.J.; Raffuse, S.; Bennett, D.H.; et al. Gestational Exposure to Particulate Matter from Urban Wildfires Is Associated with Changes in Circulating Oxylipins but Not Flame Retardants 7 to 13 Months Post-Exposure. *Environ Int* **2025**, *200*, 109468, doi:10.1016/j.envint.2025.109468.
52. Sherris, A.R.; Dearborn, L.C.; Goin, D.E.; Loftus, C.T.; Szpiro, A.A.; Casey, J.A.; Ilango, S.D.; Angal, J.; Bennett, D.H.; Buxton, M.A.; et al. Wildfire-Specific Fine Particulate Matter and Preterm Birth: A US ECHO Cohort Analysis. *Lancet Planet Health* **2025**, *9*, 101324, doi:10.1016/j.lanplh.2025.101324.
53. Ayoub, G. Neurodevelopment of Autism: Critical Periods, Stress and Nutrition. *Cells* **2024**, *13*, 1968, doi:10.3390/cells13231968.
54. Greer, J.B.; Dalsky, E.M.; Lane, R.F.; Hansen, J.D. Tire-Derived Transformation Product 6PPD-Quinone Induces Mortality and Transcriptionally Disrupts Vascular Permeability Pathways in Developing Coho Salmon. *Environ Sci Technol* **2023**, *57*, 10940–10950, doi:10.1021/acs.est.3c01040.
55. Lee, J.G.; Lee, S.M.; Hyun, M.; Heo, J.D. Tire Rubber-Derived Contaminants 6PPD and 6PPD-Quinone Reduce Attachment and Outgrowth of Trophoblast Spheroids onto Endometrial Epithelial Cells. *Ecotoxicol Environ Saf* **2025**, *290*, 117744, doi:10.1016/j.ecoenv.2025.117744.
56. Mathew, A.S.; Harjith, A.; Guruvayoorappan, C. Assessing Microplastics as a Novel Threat to Maternal-Fetal Health: Placental Barrier Penetration and Fetal Developmental Consequences. *Reproductive Toxicology* **2025**, *137*, 109021, doi:10.1016/j.reprotox.2025.109021.
57. Nadarasan, S.; Phuna, Z.X.; Zaman, R.; Tan, C.K.; Bustami, N.A.; Bin Ho, Y.; Kosasih, S.J.; Tan, E.S.S. Microplastics and Child Health: A Scoping Review of Prenatal and Early-Life Exposure Routes and Potential Health Risks. *Toxicol Rep* **2025**, *15*, 102143, doi:10.1016/j.toxrep.2025.102143.
58. Yao, K.; Kang, Q.; Liu, W.; Chen, D.; Wang, L.; Li, S. Chronic Exposure to Tire Rubber-Derived Contaminant 6PPD-Quinone Impairs Sperm Quality and Induces the Damage of Reproductive Capacity in Male Mice. *J Hazard Mater* **2024**, *470*, 134165, doi:10.1016/j.jhazmat.2024.134165.
59. Zhang, H.; Ding, X.; Zheng, H.; Ma, Q.; Zhang, T. Health Risks of Prenatal and Early-Life Microplastics Exposure: A Comprehensive Review. *Environ. Health* **2026**, *4*, 567–584, doi:10.1021/envhealth.5c00388.
60. Zhang, M.; Yin, H.; Tan, J.; Wang, X.; Yang, Z.; Hao, L.; Du, T.; Niu, Z.; Ge, Y. A Comprehensive Review of Tyre Wear Particles: Formation, Measurements, Properties, and Influencing Factors. *Atmospheric Environment* **2023**, *297*, 119597, doi:10.1016/j.atmosenv.2023.119597.
61. Zhao, H.N.; Thomas, S.P.; Zylka, M.J.; Dorrestein, P.C.; Hu, W. Urine Excretion, Organ Distribution, and Placental Transfer of 6PPD and 6PPD-Quinone in Mice and Potential Developmental Toxicity through Nuclear Receptor Pathways. *Environ Sci Technol* **2023**, *57*, 13429–13438, doi:10.1021/acs.est.3c05026.
62. Goodrich, A.J.; Tancredi, D.J.; Ludeña, Y.J.; Bennett, D.H.; Hertz-Picciotto, I.; Schmidt, R.J. Professionally and Non-Professionally Applied Household Insecticides during Pregnancy and Early Life and Their Associations with Autism Spectrum Disorder and Developmental Delay in the CHARGE Case-Control Study. *Environ Res* **2025**, *267*, 120651, doi:10.1016/j.envres.2024.120651.

63. Goodrich, A.J.; Tancredi, D.J.; Ludeña, Y.J.; Roudneva, E.; Schmidt, R.J.; Hertz-Picciotto, I.; Bennett, D.H. Pet Flea and Tick Control Exposure During Pregnancy and Early Life Associated with Decreased Cognitive and Adaptive Behaviors in Children with Developmental Delay and Autism Spectrum Disorder. *Int J Environ Res Public Health* **2025**, *22*, 1149, doi:10.3390/ijerph22071149.
64. Grandjean, P.; Landrigan, P.J. Neurobehavioural Effects of Developmental Toxicity. *Lancet Neurol* **2014**, *13*, 330–338, doi:10.1016/S1474-4422(13)70278-3.
65. Grandjean, P.; Landrigan, P.J. Developmental Neurotoxicity of Industrial Chemicals. *The Lancet* **2006**, *368*, 2167–2178, doi:10.1016/S0140-6736(06)69665-7.
66. Kalkbrenner, A.E.; Schmidt, R.J.; Penlesky, A.C. Environmental Chemical Exposures and Autism Spectrum Disorders: A Review of the Epidemiological Evidence. *Curr Probl Pediatr Adolesc Health Care* **2014**, *44*, 277–318, doi:10.1016/j.cppeds.2014.06.001.
67. Lyall, K.; Ames, J.L.; Pearl, M.; Traglia, M.; Weiss, L.A.; Windham, G.C.; Kharrazi, M.; Yoshida, C.K.; Yolken, R.; Volk, H.E.; et al. A Profile and Review of Findings from the Early Markers for Autism Study: Unique Contributions from a Population-Based Case-Control Study in California. *Mol Autism* **2021**, *12*, 24, doi:10.1186/s13229-021-00429-7.
68. Midya, V.; Bello, G.A.; Gomez, L.A.; Marin, M.R.; Piyankarage, S.C.; Elhlou, S.; Chumber, J.; Jaramilo, J.; Dessalle, S.; Yitshak-Sade, M.; et al. Rule-out Test for Autism Using Machine-Learning Analysis of Molecular Temporal Dynamics in Hair - a Multicenter Study 2025.
69. Rossignol, D.A.; Genuis, S.J.; Frye, R.E. Environmental Toxicants and Autism Spectrum Disorders: A Systematic Review. *Transl Psychiatry* **2014**, *4*, e360–e360, doi:10.1038/tp.2014.4.
70. Stojsavljević, A.; Lakićević, N.; Pavlović, S. Mercury and Autism Spectrum Disorder: Exploring the Link through Comprehensive Review and Meta-Analysis. *Biomedicines* **2023**, *11*, 3344, doi:10.3390/biomedicines11123344.
71. Jang, H.; Bennett, D.H.; Hoofnagle, A.N.; Pearce, E.N.; Schmidt, R.J.; Shin, H.-M. Associations of Maternal Urinary Perchlorate, Thiocyanate, and Nitrate with Maternal Thyroid Function during Pregnancy: Modification by Iodine Status in the MARBLES Study. *Environ Res* **2026**, *306*, 125122, doi:10.1016/j.envres.2026.125122.
72. Petrick, L.M.; Dassanayake, P.S.; Gennings, C.; Guan, H.; Akintunde, B.; Martinez, M.; Regmi, P.; Hertz-Picciotto, I.; Arora, M.; Bennett, D.H. Fetal Exposure to Persistent Organic Pollutants and Childhood Autism Risk. *Environ Res* **2026**, *306*, 125179, doi:10.1016/j.envres.2026.125179.
73. Bragg, M.G.; Rando, J.; Carroll, K.N.; Eick, S.M.; Karagas, M.R.; Lin, P.-I.; Schmidt, R.J.; Lyall, K.; Smith, P.B.; Newby, L.K.; et al. The Association of Prenatal Dietary Factors with Child Autism Diagnosis and Autism-Related Traits Using a Mixtures Approach: Results from the Environmental Influences on Child Health Outcomes Cohort. *The Journal of Nutrition* **2025**, *155*, 1938–1951, doi:10.1016/j.tjnut.2025.02.025.
74. Buckley, J.P.; Pacyga, D.C.; Xun, X.; Barr, D.B.; Barrett, E.S.; Bastain, T.; Bennett, D.H.; Braun, J.M.; Breton, C.V.; Carignan, C.; et al. Gestational Exposure to 10 Classes of Priority Chemicals and Birth Outcomes in the ECHO Cohort. *JAMA Netw Open* **2026**, *9*, e2618883, doi:10.1001/jamanetworkopen.2026.18883.
75. Pacyga, D.C.; Xun, X.; Bennett, D.; Bloom, M.S.; Braun, J.M.; Carignan, C.C.; Howe, C.G.; Karagas, M.R.; Kannan, K.; Meeker, J.D.; et al. Dietary Guidelines Adherence and Pregnancy Exposure to 10 Classes of Priority Chemicals: An Observational Study in the Environmental Influences on Child Health Outcomes Cohort. *Am J Clin Nutr* **2026**, 101393, doi:10.1016/j.ajcnut.2026.101393.
76. Hall, K.D.; Ayuketah, A.; Brychta, R.; Cai, H.; Cassimatis, T.; Chen, K.Y.; Chung, S.T.; Costa, E.; Courville, A.; Darcey, V.; et al. Ultra-Processed Diets Cause Excess Calorie Intake and Weight Gain: An Inpatient Randomized Controlled Trial of Ad Libitum Food Intake. *Cell Metabolism* **2019**, *30*, 67-77.e3, doi:10.1016/j.cmet.2019.05.008.
77. Lane, M.M.; Lotfaliany, M.; Forbes, M.; Loughman, A.; Rocks, T.; O'Neil, A.; Machado, P.; Jacka, F.N.; Hodge, A.; Marx, W. Higher Ultra-Processed Food Consumption Is Associated with Greater High-

Sensitivity C-Reactive Protein Concentration in Adults: Cross-Sectional Results from the Melbourne Collaborative Cohort Study. *Nutrients* **2022**, *14*, 3309, doi:10.3390/nu14163309.

78. Edalati, S.; Bagherzadeh, F.; Jafarabadi, M.A.; Ebrahimi-Mamaghani, M. Higher Ultra-Processed Food Intake Is Associated with Higher DNA Damage in Healthy Adolescents. *British Journal of Nutrition* **2021**, *125*, 568–576, doi:10.1017/S0007114520001981.
79. Quetglas-Llabrés, M.M.; Monserrat-Mesquida, M.; Bouzas, C.; Mateos, D.; Lucía Ugarriza; Gómez, C.; Tur, J.A.; Sureda, A. Oxidative Stress and Inflammatory Biomarkers Are Related to High Intake of Ultra-Processed Food in Old Adults with Metabolic Syndrome. *Antioxidants* **2023**, *12*, 1532, doi:10.3390/antiox12081532.
80. Babalola, O.O.; Akinnusi, E.; Bridget, K.; Oyubu, G.; Ajiboye, S.A.; Waheed, S.A.; Collette, A.C.; Adebimpe, H.O.; Nwokafor, C.V.; Oni, E.A.; et al. The Impact of Ultra-Processed Foods on Cardiovascular Diseases and Cancer: Epidemiological and Mechanistic Insights. *Aspects of Molecular Medicine* **2025**, *5*, 100072, doi:10.1016/j.amolm.2025.100072.
81. Ultra-Processed Diet, Systemic Oxidative Stress, and Breach of Immunologic Tolerance. *Nutrition* **2021**, *91–92*, 111419, doi:10.1016/j.nut.2021.111419.
82. Anih, D.; Tatah, V.; Uhwuoke, K.; Boyi, R. Cellular Disruption by Ultra-Processed Foods: Mechanistic Pathways Linking Industrial Additives to Metabolic Disease. *Res J Microbiol 20*, 20–41.
83. Ayoub, G. Autism Spectrum Disorder as a Multifactorial Disorder: The Interplay of Genetic Factors and Inflammation. *International Journal of Molecular Sciences* **2025**, *26*, 6483, doi:10.3390/ijms26136483.
84. Ayoub, G. Neurodevelopmental Impact of Maternal Immune Activation and Autoimmune Disorders, Environmental Toxicants and Folate Metabolism on Autism Spectrum Disorder. *Current Issues in Molecular Biology* **2025**, *47*, 721, doi:10.3390/cimb47090721.
85. LeBlanc, J.J.; Fagiolini, M. Autism: A "Critical Period" Disorder? *Neural Plast* **2011**, *2011*, 921680, doi:10.1155/2011/921680.
86. Vecchione, R.; Westlake, M.; Bragg, M.G.; Rando, J.; Bennett, D.H.; Croen, L.A.; Dunlop, A.L.; Ferrara, A.; Hedderson, M.M.; Kerver, J.M.; et al. Maternal Dietary Patterns During Pregnancy and Child Autism-Related Traits in the Environmental Influences on Child Health Outcomes Consortium. *Nutrients* **2024**, *16*, 3802, doi:10.3390/nu16223802.
87. Yenkoyan, K.; Mkhitaryan, M.; Bjørklund, G. Environmental Risk Factors in Autism Spectrum Disorder: A Narrative Review. *Curr Med Chem* **2024**, *31*, 2345–2360, doi:10.2174/0109298673252471231121045529.
88. Sotelo-Orozco, J.; Taft, D.H.; Al-Oboudi, J.; Baikie, B.C.; Lake, C.; Miller, M.; Mills, D.A.; Tancredi, D.J.; Schmidt, R.J.; Hertz-Picciotto, I.; et al. Mixed Evidence for Impact of Early Infant Gut Microbiome and Later Development of Autism Spectrum Disorder in the MARBLES Prospective Cohort Study. *Autism Research* **2026**, *19*, e70207, doi:10.1002/aur.70207.
89. Agrawal, S.; Rao, S.; Whitehouse, A.; Alvares, G.A.; Kulkarni, A.; Taylor, J.A.; Conway, P.L.; Thomas, T.; Patole, S. Effect of Probiotic Supplementation on Gut Microbiota in Children with Autism: A Pilot Randomised Controlled Trial. *Nutrients* **2026**, *18*, 2079, doi:10.3390/nu18132079.
90. Engevik, M.A.; Morra, C.N.; Röth, D.; Engevik, K.; Spinler, J.K.; Devaraj, S.; Crawford, S.E.; Estes, M.K.; Kalkum, M.; Versalovic, J. Microbial Metabolic Capacity for Intestinal Folate Production and Modulation of Host Folate Receptors. *Front Microbiol* **2019**, *10*, 2305, doi:10.3389/fmicb.2019.02305.
91. Michel, A.; Pourié, G.; Kökten, T. Consequences of Western and Mediterranean Diets' Nutrients on the Microbiota-Gut-Brain Axis. *Nutrients* **2026**, *18*, 1258, doi:10.3390/nu18081258.
92. Laugesen, K.; Skajaa, N.; Petersen, I.; Andersen, M.S.; Feldt-Rasmussen, U.; Kejlberg Al-Mashhadi, S.; Stewart, P.; Jørgensen, J.O.L.; Sørensen, H.T. Mental Disorders Among Offspring Prenatally Exposed to Systemic Glucocorticoids. *JAMA Netw Open* **2025**, *8*, e2453245, doi:10.1001/jamanetworkopen.2024.53245.
